# Holocene bidirectional river system along the Kenya Rift and its influence on East African faunal exchange and diversity gradients

**DOI:** 10.1073/pnas.2121388119

**Published:** 2022-06-27

**Authors:** René Dommain, Simon Riedl, Lydia A. Olaka, Peter deMenocal, Alan L. Deino, R. Bernhart Owen, Veronica Muiruri, Johannes Müller, Richard Potts, Manfred R. Strecker

**Affiliations:** ^a^Institute of Geosciences, University of Potsdam, 14476 Potsdam-Golm, Germany;; ^b^Human Origins Program, National Museum of Natural History, Smithsonian Institution, Washington, DC 20013;; ^c^Asian School of the Environment, Nanyang Technological University, 639798, Singapore, Singapore;; ^d^Department of Earth and Climate Science, University of Nairobi, 00100, Nairobi, Kenya;; ^e^Institute for Climate Change and Adaptation, University of Nairobi, 00100, Nairobi, Kenya;; ^f^Woods Hole Oceanographic Institution, Woods Hole, MA 02543;; ^g^Division of Biology and Paleo Environment, Lamont-Doherty Earth Observatory, Palisades, NY 10964;; ^h^Berkeley Geochronology Center, Berkeley, CA 94709;; ^i^Department of Geography, Hong Kong Baptist University, Kowloon Tong, Hong Kong;; ^j^Department of Earth Sciences, National Museums of Kenya, Nairobi 00100, Kenya;; ^k^Museum für Naturkunde, Leibniz-Institut für Evolutions und Biodiversitätsforschung, 10115 Berlin, Germany

**Keywords:** East Africa, biogeography, biodiversity, hydrological connectivity, Holocene

## Abstract

East Africa is a global biodiversity hotspot and exhibits distinct longitudinal diversity gradients from west to east in freshwater fishes and forest mammals. The assembly of this exceptional biodiversity and the drivers behind diversity gradients remain poorly understood, with diversification often studied at local scales and less attention paid to biotic exchange between Afrotropical regions. Here, we reconstruct a river system that existed for several millennia along the now semiarid Kenya Rift Valley during the humid early Holocene and show how this river system influenced postglacial dispersal of fishes and mammals due to its dual role as a dispersal corridor and barrier. Using geomorphological, geochronological, isotopic, and fossil analyses and a synthesis of radiocarbon dates, we find that the overflow of Kenyan rift lakes between 12 and 8 ka before present formed a bidirectional river system consisting of a “Northern River” connected to the Nile Basin and a “Southern River,” a closed basin. The drainage divide between these rivers represented the only viable terrestrial dispersal corridor across the rift. The degree and duration of past hydrological connectivity between adjacent river basins determined spatial diversity gradients for East African fishes. Our reconstruction explains the isolated distribution of Nilotic fish species in modern Kenyan rift lakes, Guineo-Congolian mammal species in forests east of the Kenya Rift, and recent incipient vertebrate speciation and local endemism in this region. Climate-driven rearrangements of drainage networks unrelated to tectonic activity contributed significantly to the assembly of species diversity and modern faunas in the East African biodiversity hotspot.

## Significance

Although biodiversity in East Africa is overall extremely high, species richness is not geographically uniform for fishes and mammals. We investigated the biogeographic relevance of past river activity in the Kenya Rift. We show that during a humid period 12,000 to 8,000 years ago, a river system connected currently isolated rift lakes and was partly connected to the Nile. While this river system formed pathways for the dispersal of fishes between lakes, it also acted as a barrier to the range expansion of forest mammals. This fairly recent hydrological connectivity between lakes has been a key driver of modern biodiversity patterns in East Africa. Climate-driven changes in drainage networks on multimillennial timescales are an important hypothesis in biodiversity research.

**Fig. 1.**
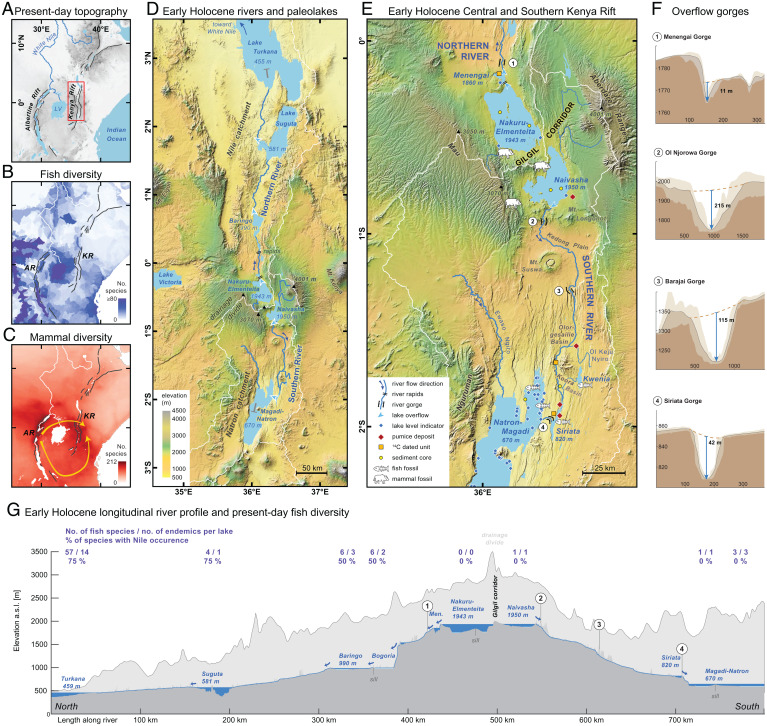
Study region and reconstruction of the early Holocene river system in the Kenya Rift Valley. (*A*) Study region with modern topography of the East African Rift System. (*B*) Present-day species richness of fishes (class Actinopterygii) in East Africa from the IUCN (94). (*C*) Present-day species richness of mammals in East Africa from the IUCN (94); yellow arrows indicate proposed dispersal pathways of Kingdon (22, 23). (*D*) Reconstruction of the early Holocene river system between overflowing lakes along the Kenya Rift Valley; elevations denote overflow levels, dark-blue arrows, river flow directions. (*E*) Study sites, fossil localities, and reconstruction of the early Holocene river system in the Central and South Kenya Rift. (*F*) Cross-sections of overflow gorges used by the Northern (1) and Southern (2–4) Rivers (locations marked in panels *E* and *G*). (*G*) Longitudinal river profiles of the Northern and Southern Rivers along the Kenya Rift Valley with data on present-day fish diversity (excluding introduced species) and biogeography of Kenyan rift lakes (*SI Appendix*, Table S1). Dark blue areas denote early Holocene lake levels and rivers, and light blue denotes present-day lake levels. Dark gray area shows the topography of the rift valley floor used by the rivers, and light gray shows the topography of the adjacent rift shoulders. Note the dome-shaped topography of the East African Plateau.

Equatorial East Africa is one of the most biodiverse regions on Earth and hosts the greatest vertebrate diversity in Africa (1, 2). The East African highlands are a significant reservoir of highly diverse and threatened terrestrial vascular plant flora and vertebrate fauna, including many rare mammals, and have been designated as the Eastern Afromontane Hotspot (3). The African Great Lakes such as Lake Victoria are outstanding centers of endemism and rapid speciation, containing some of the most diverse lacustrine fish faunas in the world (3–5). How was this exceptional biodiversity assembled? While local species diversity has been shown to result from in situ speciation in geographically isolated settings such as mountains or lakes (6, 7) and from adaptive radiation following colonization of newly available habitats (8), the influence of past faunal exchange between Afrotropical regions in assembling faunas is less understood. Biotic interchange is an important driver in the assembly of regional biotas and of tropical biodiversity in general (9) and may be of particular relevance for East Africa, where species richness exhibits marked spatial gradients (Fig. 1) and where disjunct distribution patterns suggest a history of dispersal across biomes and connectivity between distant regions. However, the drivers of gradients in species diversity as well as dispersal routes for presently isolated vertebrate populations remain poorly studied.

Although spatial diversity patterns in East African fishes are regionally complex (Fig. 1*B*), diversity generally declines from west to east. Consistent with this pattern, the lakes of the Albertine Rift to the west have a higher species richness than most lakes of the Kenya Rift to the east (5, 10) (*SI Appendix*, Table S1). In addition, along the Kenya Rift, fish diversity declines from north to south and, notably, some of the endorheic lakes in the northern Kenya Rift such as Lake Turkana host isolated populations of Nilotic species like Nile tilapia (*Oreochromis niloticus*) (10) (Fig. 1*G* and *SI Appendix*, Table S1). These isolated populations have been interpreted to reflect past hydrological connectivity with the Nile River (11, 12). The last connection between Lake Turkana and the Nile River was from ca. 11.5 to 8.5 ka (13) during the African Humid Period (AHP)—a significantly wetter climatic phase in East Africa (14). Elsewhere in Africa larger river systems existed during the AHP, which facilitated the dispersal of species (15). Yet in East Africa the details of past hydrological connectivity between lakes and rivers are only fragmentarily known (16) and are insufficient to explain the distribution of fish species (17).

Longitudinal diversity gradients across equatorial East Africa also exist for mammals (Fig. 1*C*). Species richness of forest-dependent mammals decreases linearly eastward from the Congo Basin and Albertine Rift highlands in the west to the forests of eastern Kenya (18). The Victoria Nile in Uganda marks the easternmost range limit for more than 20 forest-adapted mammal species (19), attesting to its role as a riverine barrier (20). Farther east, the Kenya Rift also affects mammalian species distribution, as the semiarid rift valley represents an impassable barrier for rainforest taxa due to the lack of forest cover (19, 21). Remarkably though, isolated Kenyan forests on the eastern side of the rift, including the montane forests of the Aberdare Range and Mount Kenya (Fig. 1*E*), harbor various mammal species of Guineo-Congolian distribution such as the bongo (*Tragelaphus eurycerus*) and black-fronted duiker (*Cephalophus nigrifrons*) that are separated from their main ranges by up to 800 km (21, 22) (*SI Appendix*, Fig. S2). Kingdon 1981 and 1971 (22, 23) postulated two dispersal pathways for these disjunct Guineo-Congolian forest species: a northern route north of Lake Victoria via Mount Elgon and extending across the Kenya Rift to the east, and a southern route south of Lake Victoria via the Eastern Arc Mountains (Tanzania) and Kilimanjaro to eastern Kenya (Fig. 1*C*). Forest expansion during Quaternary humid periods is presumed to have opened these proposed routes as corridors between the Congo Basin and Kenya (23, 24). However, during past humid periods, such as the AHP, larger rivers may have severed forested dispersal pathways. Here, we tested the viability of these dispersal routes with a reconstruction of the paleo-drainage network, explicitly recognizing that rivers represent not only important ecological corridors (25–27) but also significant barriers to the dispersal of nonvolant mammals (6, 20, 28).

In our study, we hypothesize that the peculiar biogeography of isolated fish and mammal occurrences along the Kenya Rift and the longitudinal gradients in species diversity across East Africa were influenced by past river activity during the early Holocene. To test this hypothesis, we reconstructed the past hydrological connectivity of lake basins in the Kenya Rift and investigated the dual role of (paleo)rivers as corridors and barriers to early Holocene vertebrate dispersal. The specific objectives of our investigations were 1) to reconstruct past rivers and connectivity between currently isolated lakes in the Kenya Rift for the AHP, 2) to determine the degree of faunal connectivity and exchange for fish faunas within and between East African paleo-river basins, 3) to assess Kingdon’s hypothesis of northern and southern dispersal routes for forest fauna (22, 23) with respect to our reconstructions of early Holocene landscape settings and river systems, and 4) to evaluate the relevance of riverine corridors and barriers to faunal exchange and longitudinal diversity gradients in East Africa during the Quaternary.

To address these issues, we use field mapping of fluvial deposits and lake overflow structures, digital elevation models combined with differential GPS measurements of absolute elevations, and isotope and paleo-ecological analyses and radiometric dating of relevant lacustrine and fluvial sediments, among other methods, to present geomorphological, geochronological, sedimentological, and fossil evidence for the hydrological connectivity of lake basins in the Kenya Rift during the AHP (see *Materials and Methods*). We determined the timing of lake connectivity by integrating 159 lake level–indicating radiocarbon dates from eight lake basins (including 22 new ^14^C dates, two new ^40^Ar/^39^Ar dates, and ^14^C-reservoir-corrected chronologies for four lake basins) and the permanence of river flow with a detailed oxygen isotope record from the southern Kenya Rift. Fossil fish discoveries, published fossil and taxonomic data, and our reconstructions of dispersal pathways and dated area cladograms were used to infer East African faunal exchange, geographic isolation, and the Holocene assembly of regional faunas and diversity.

## Results

### The Early Holocene River System.

**Fig. 2.**
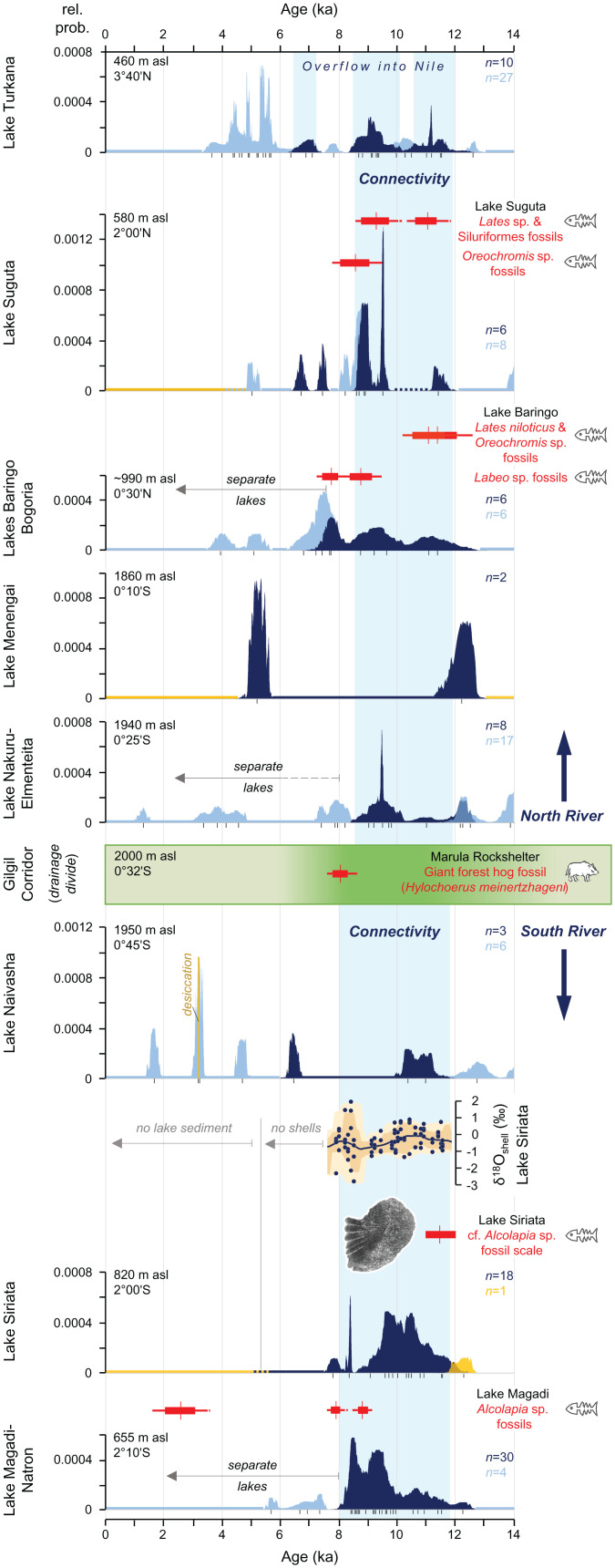
Chronology of river activity and lake overflow in the Kenya Rift Valley over the past 14 ka. Shown is the sequence of connected lakes and overflow dates for the Northern River (*Top*) and Southern River (*Bottom*) with the chronology based on CPDs of radiocarbon dates (*SI Appendix*, Table S6) for each connected lake basin (dark blue: overflow periods; light blue: closed lake basin conditions; yellow: dry basins; vertical axis is probability in annual bins; black tick marks underneath CPDs show median ages of individual calibrated ^14^C dates; *n* denotes number of radiocarbon dates/basin). Period of overflow along the entire Kenya Rift is indicated by the vertical light-blue band. The green horizontal band denotes the Gilgil Corridor between the two rivers; darker green color indicates forested period. Radiocarbon-dated fossil evidence for forest mammals and fishes is shown in red (calibrated ^14^C dates of fossils: red bars: 1σ range; horizontal red line: 2σ range; vertical red line: median age; ^14^C dates in *SI Appendix*, Table S2), oxygen isotope record for Siriata (dots denote single data points, line is median probability, envelopes are 68% [dark] and 95% [light] confidence intervals), and photo of *Oreochromis* cf. *Alcolapia* spp. fossil fish scale from Siriata (*SI Appendix*, Fig. S13).

We reconstructed a 670 km long, bidirectional river system along the entire Kenya Rift Valley for the early Holocene (12 to 8 ka), consisting of two major rivers running in opposite directions (Fig. 1*D*). Both rivers commenced in the highest area of the Central Kenya Rift at latitude ∼0.3° S and flowed down the > 1 km elevation gradient of the East African Plateau, one toward the north and the other toward the south (Fig. 1*G* and *SI Appendix*, Figs. S1 and S3). The Northern River was fed by the overspill from Lake Nakuru-Elmenteita and the Southern River by the overspill of Lake Naivasha. These two rivers connected all the major, presently isolated lake basins of the Kenya Rift and thus represented aquatic dispersal routes between the connected chain of lakes. The Northern River connected a chain of lakes consisting of Lakes Nakuru-Elmenteita, Menengai, Bogoria, Baringo, Suguta, and Turkana. Since Lake Turkana was also connected to the White Nile via the Pibor-Sobat River (16, 29), the Nile catchment effectively extended upstream into the central Kenyan highlands during this time. In contrast, the Southern River provided major inflow to the currently endorheic Lake Magadi–Natron Basin in the southern Kenya Rift and connected Lake Naivasha with Lake Siriata and the joint Lake Magadi–Natron (Fig. 1*E*). Our compilation of radiocarbon dates of overflow lake levels from all lake basins of the Kenya Rift reveals a virtually synchronous onset of long-lasting connectivity at the beginning of the Holocene, indicating permanent flow in the two rivers over more than three to four millennia (Fig. 2).

We measured river channel widths of between 20 and > 80 m at different field locations of both the Southern and Northern River (Fig. 1*E*). Our field observations showed that past flood levels occupied the entire width of the valley bottoms. The two rivers exhibited flat channel bottoms and at various sections polished and smooth rock surfaces, fluted bedrock surfaces, and plunge pools across the entire riverbeds (*SI Appendix*, Fig. S4). These geomorphic features indicate the effect of abrading rock fragments that were transported within these channels and represent vestiges of increased abrasion under past hydrological regimes with enhanced stream flow, more transported load, and bedrock incision (30, 31). Digital elevation model (DEM)–based swath profiles of presently dry river gorges provide additional support for wide riverbeds during periods of past river activity (Fig. 1*F*). We inferred that this large, perennial river system represented an important physical dispersal barrier for nonvolant terrestrial vertebrate species within the Kenya Rift.

The only permanent land bridge and hence the only terrestrial corridor across the Kenya Rift during the interval of river activity was the 5- to 22 km wide drainage divide at Gilgil (hereafter, Gilgil Corridor) between Lake Nakuru–Elmenteita and Lake Naivasha at an elevation estimated from present data at ∼2,050 m asl. Its function as a dispersal pathway for forest mammals is confirmed by fossil occurrences of the forest-dependent giant forest hog (*Hylochoerus meinertzhageni*) in the 8 ka old Marula Rock shelter (2,000 m asl) (32) and in Gamble’s Cave (1,934 m asl) (33), both located within the Gilgil Corridor (Figs. 1*E* and 2 and *SI Appendix*, Fig. S6).

### The Northern River.

The Northern River traversed more than 450 km before flowing into Lake Turkana (Fig. 1). Along its course, it dropped 1,486 m in elevation, with gradients ranging between 1.5 and 8.4 m/km (mean, 3.8 m/km). The river integrated seven lake catchments of the central and northern Kenya Rift Valley into a large ∼175,000 km^2^ subcatchment of the Nile River. Its source was Lake Nakuru–Elmenteita, which maintained an overflow highstand level at 1,943 m asl between 12.0 and 8.6 ka (*SI Appendix*, Table S2). To the north, this lake bordered directly on the Menengai Volcano and spilled via 80 m steep rapids into the volcano’s caldera, where a contemporaneous crater lake—referred to here as Lake Menengai—formed with a water level of 1,860 m asl (34, 35) (Fig. 2 and *SI Appendix*, Figs. S4 and S5). Lake Menengai drained northward (34), and our fieldwork revealed that the outflow carved a now-abandoned river gorge through the 36 ka old (36) Menengai pyroclastic deposits. This 4.5 km long gorge, which is up to 11 m deep and once hosted a 20 to 30 m wide river, represents the uppermost paleo-channel bed of the Northern River (Fig. 1 *E* and *F*). Thirty kilometers downstream of the gorge, the river descended 500 m over a course of only 10 km, which constituted a vertical drop over a 260 m steep escarpment, before emptying into Lake Baringo–Bogoria (Fig. 1*G*). The resulting rapids must have acted as a major barrier to the upstream dispersal of aquatic organisms.

Our TanDEM-X DEM-based river routing and ^14^C reservoir–corrected overflow chronologies confirmed earlier suggestions of a river connection between Lakes Baringo–Bogoria, Suguta, and Turkana (37, 38), forming a section of the Northern River. Lake Baringo maintained an overflow level at 990 m asl between 12 and ca. 7.8 ka (Fig. 2) and was connected via the Northern River with Lake Suguta. Lake Suguta spilled over between 11.7 and 8.6 ka and again between ∼7.6 and 6.5 ka and was connected with Lake Turkana immediately downstream (38). Finally, Lake Turkana maintained an overflow level at 459 m asl (13), from which it drained into the White Nile (16, 29) during three intervals—from 12.0 to 10.6 ka, from 10.1 to 8.5 ka, and from 7.2 to 6.5 ka—documenting ca. 3,700 y of connectivity with the Nile. Fossils of various fish taxa, including Nilotic species such as Nile perch (*Lates niloticus*) have been found in early Holocene deposits of Lake Suguta and Lake Baringo (39, 40) (Fig. 2 and *SI Appendix*, Fig. S6), demonstrating active upstream dispersal along the Northern River and a contemporaneous biogeographic connection with the Nile Basin.

### The Southern River.

The Southern River, an aquatic dispersal pathway between Lakes Naivasha and Magadi–Natron, dropped 1,221 m over its 175 km long course, with a river gradient of 4.8 to 10 m/km (mean, 7.0 m/km). This river integrated four presently isolated catchments into a single 31,950 km^2^ large endorheic drainage basin, the Southern River basin (*SI Appendix*, Figs. S1 and S3). The river originated with an outlet immediately south of Lake Naivasha in the Ol Njorowa Gorge, which is up to 215 m deep and 150 to 300 m wide (Fig. 1*F* and *SI Appendix*, Fig. S4) and has previously been proposed as an outlet of Lake Naivasha (29, 41). According to diatom records from Lake Naivasha, which indicate the existence of an open freshwater lake (41, 42), this gorge last served as an outlet between 11.4 and 6.2 ka (Fig. 2). Downstream of the outlet, the river flowed east of Suswa Volcano and followed the (maximally) 115 m deep and 50 to 200 m wide Barajai Gorge, part of the Kedong Valley, before flowing through the Olorgesailie Basin (Fig. 1 *E*–*G*). The Southern River aggraded an almost-level, > 1 km wide, and at least 13 m thick alluvial plain within this basin, which we dated to 7.0 ka at 5.8 m below surface and to 4.1 ka at 1.8 m below surface (*SI Appendix*, Fig. S4 and Table S3). The river then entered the Koora Basin and provided the main inflow to Lake Siriata. Several radiocarbon-dated, mollusk-bearing lacustrine deposits (one of which overlies a paleosol dated to the Younger Dryas at 12.3 ka) and the presence of a single isolated shoreline at 819.5 m asl document the existence of a 30 km^2^ and 55 m deep lake (Lake Siriata) between 12.0 and at least 7.6 ka, but probably until 5.5 ka (Fig. 2 and *SI Appendix*, Figs. S4 and S7). This highstand shoreline, dated by ^40^Ar/^39^Ar to 11.0 ± 1.0 ka on floated pumice clasts (*SI Appendix*, Figs. S8–S12 and Tables S4 and S5), has the same elevation as the outlet of this lake, which drained via an up to 60 to 80 m wide river into Lake Magadi–Natron through a now dry 4 km long and up to 42 m deep incised bedrock gorge (Fig. 1*F* and *SI Appendix*, Figs. S4 and S5). Dated stromatolites around modern Lakes Magadi and Natron confirm that these two basins formed a single, 55 m deep lake (43) (Fig. 1*B*) during the interval from ∼12.4 to 8.2 ka, according to our ^14^C reservoir correction. By approximately 8.0 ka, the lake level had shrunk until it formed two discrete waterbodies, yet Lake Magadi continued to receive inflow from Lake Siriata via the last section of the Southern River and maintained a higher water level until at least 7.7 ka, as indicated by dated fossils of the Magadi–Natron endemic soda tilapia (*Alcolapia* sp.) from several meters above the present lake level (29) (Fig. 2).

The persistence of flow of the Southern River was inferred from our 4,000 y δ^18^O record on mollusk shells (*n* = 90) from sediments deposited in Lake Siriata (Fig. 2). This record exhibited very subdued variability, averaging −0.37‰ (SD ± 0.78‰). If inflow into Lake Siriata had been episodic, then the water would have been isotopically enriched during dry periods due to higher evaporation, as observed in modern rift lakes with intermittent inflow (44), and the δ^18^O data should show fluctuations of several per mil (13, 45). The presence of a single isolated shoreline at the outlet elevation also indicates a stable lake level with constant inflow and outflow. The Southern River can thus be viewed as a perennial river that served as both a dispersal corridor for aquatic animals and a potential barrier for terrestrial organisms. The δ^18^O data showed greater variability and more enriched values (by up to 1.9‰) only between 8.35 and 8.15 ka, a period that also coincided with the cessation of overflow conditions along the Northern River (Fig. 2) and may suggest a regional hydrological response to the “8.2 ka event,” which, in East Africa, was a dry interval (46). Lake Siriata, however, is likely to have maintained a permanently open throughflow system between Lake Naivasha and Lake Magadi–Natron.

The inference of a 151 km long direct hydrological connection between Lakes Naivasha and Siriata is further supported by the presence of well-rounded pumice, which can be used as tracers for water flow due to their buoyancy. We found rounded pumice clasts (2 to 7 cm) on a lake–level highstand shoreline of Lake Naivasha at 2,005 m asl, similarly rounded pumice (∼2 cm) on the Southern River’s floodplain at Olorgesailie (972 m asl), and rounded pumice (2 to 15 cm) in beach and diatomite deposits of Lake Siriata (at 819.1 and 807 m asl, yielding a ^40^Ar/^39^Ar age of 11.0 ± 1.0 ka) (Fig. 1*E* and *SI Appendix*, Figs. S4, S8, and S12). These pumice clasts are likely to have originated from volcanic centers of the Central Kenya Rift located in the Southern River’s catchment area (47), such as Mount Longonot or Mt. Suswa (Fig. 1*E*). A direct connection between Lakes Siriata and Magadi–Natron was confirmed by fish fossils found in Lake Siriata sediments. One fish scale retrieved from sediments dated ∼11.4 ka was identified as a member of *Oreochromis* cf. subgenus *Alcolapia* (Cichlidae) (Fig. 2 and *SI Appendix*, Fig. S13), which is today restricted to Lakes Natron and Magadi (48). We also discovered unidentifiable fish remains dated to ∼16.9 ka in the adjacent Kwenia Basin to the east of Lake Siriata (*SI Appendix*, Figs. S4 and S6). These fossils provide evidence for aquatic faunal exchange between lakes in the southern Kenya Rift during the latest Pleistocene and early Holocene.

## Discussion

### Influence on Fish Dispersal, Distribution, and Diversity.

**Fig. 3.**
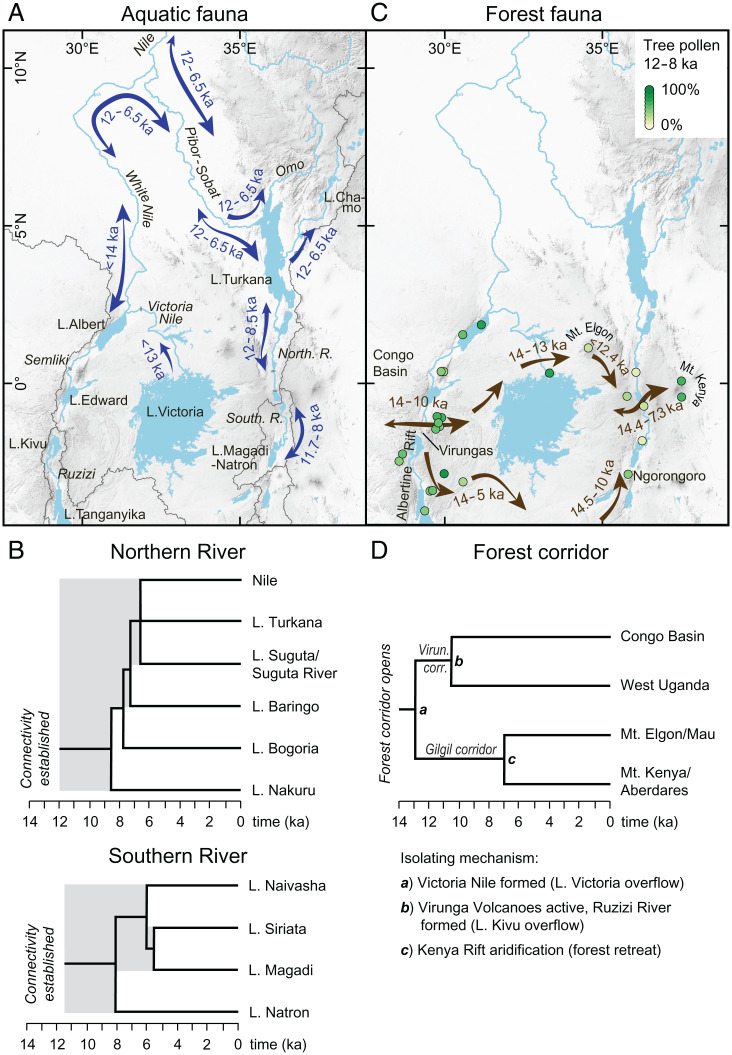
Reconstructed pathways for dispersal and faunal exchange during the early Holocene for aquatic and terrestrial-forest fauna, and geological area cladograms for river systems and forests. The cladograms provide a basis for deducing divergence dates of lacustrine fish and forest mammal populations. (*A*) Reconstructed early Holocene river networks in East Africa, with arrows denoting the directions and time of existing dispersal pathways for aquatic fauna (e.g., fishes). (*B*) Dated geological area cladograms showing the time of lake basin connectivity (gray background) and the sequence of lake separation (i.e., geographic isolation) for the Northern River lake cascade (*Top*) and Southern River lake cascade (*Bottom*). (*C*) Early Holocene forest corridors based on a compilation of tree pollen percentages from 25 pollen records (greenish dots; *SI Appendix*, Table S8). Viable dispersal pathways for forest fauna and their direction and time are denoted by brown arrows with a northern route (similar to Kingdon’s [23] northern route) and a southern route following the southern catchment boundary of Lake Victoria. (*D*) Dated geological area cladogram indicating the time of forest connection from the eastern Congo Basin to the east of the Kenya Rift Valley (i.e., Aberdares/Mount Kenya forests) and of their separation (causes of separation indicated at each node). Roots of trees in (*B*) and (*D*) indicate onset of connections, while their nodes indicate divergence times for remaining local populations. Timescales for (*A*–*D*) based on calibrated radiocarbon dates.

The opposing directions of the two rivers provide a unique framework for addressing the peculiar biogeography and patterns of species richness of freshwater fishes in the lakes of the Kenya Rift. After the prevailing aridity during the Last Glacial Maximum and early deglaciation (ca. 24 to 16 ka) when most lakes in East Africa dried out (5, 49) (*SI Appendix*, Fig. S1), the East African lakes existed as isolated basins for a few thousand years. During their early postglacial existence, the young lakes could have received colonizing species only from refugia that existed within their catchments, which would have contributed to low species richness. Lakes Turkana (Kenya Rift) and Edward (Albertine Rift) were the sole water bodies maintained throughout the dry glacial period (5)—probably a significant factor contributing to their presently high levels of species richness and endemicity (Fig. 1*G*). The establishment of a larger river network in East Africa that connected many of the lakes during the early Holocene (Fig. 3*A*) would have enhanced faunal exchange, immigration, and local species richness in most rift lakes. The size of the paleo-rivers, which were more than 20 to 40 m wide (Fig. 1*F*), would have supported effective dispersal, as the movement distance of fish is positively correlated with stream width (50). Large and deep early Holocene lakes provided more space, and hence probably more diverse habitats, while the general freshness of the lake waters (42) would have imposed few physiological constraints on immigrating species. These ecological conditions should theoretically have supported greater species richness of aquatic fauna in each lake (7) than before and after the AHP.

The entire northern lake cascade, beginning at Lake Nakuru–Elmenteita, was ultimately connected to the White Nile until ca. 8.6 ka (Fig. 3*A*), and consequently the entire Northern River basin became part of the Nilo–Sudan ichthyofaunal province (*sensu* 12) for at least 3,400 y. This connection potentially allowed for faunal exchange between the Northern River and the Nile and further with Lakes Albert, Edward, Kyoga, and Victoria, which were all connected to the White Nile by 14 or 13 ka (45, 49) (Fig. 3*A*). Fish species dispersal, however, was limited by physical barriers. The rapids of the Northern River upstream of Lake Bogoria and into the Menengai Caldera (Fig. 1 *D* and *G*) would have restricted postglacial colonization of Lake Nakuru–Elmenteita. This topographic isolation partly explains the complete absence of native fish species in the modern Lakes Nakuru and Elmenteita (Fig. 1*G* and *SI Appendix*, Table S1). Similarly, the Semliki River Rapids in the Albertine Rift restricted upstream dispersal toward Lake Edward, and the 42 m high Murchison Falls of the Victoria Nile precluded the colonization of Lakes Victoria and Kyoga by the White Nile (12, 51) and its tributary, the Northern River (Fig. 3*A*). In contrast, Lakes Victoria, Kyoga, Edward, and Albert were potential source areas for biota migrating into the lakes of the Northern River between approximately 12 and 8.6 to 8.2 ka (Fig. 3*A*). The high proportion of Nilotic species (50 to 75%) in the modern lakes of Turkana, Baringo, and Bogoria and in the Suguta River (Fig. 1*G*) strongly supports that the colonization of these lakes occurred from source areas in the Nile Basin. The long recognized faunal similarities between Lake Turkana and Lake Albert (11), which share 26 fish species (∼45% of their fauna; *SI Appendix*, Table S1), provide further corroboration of biotic interchange along the 2,500 km long water course between Lakes Turkana and Albert (Fig. 3*A*). Given a cumulative time of hydrological connectivity between these two lakes of approximately 3,700 y, a species would have required a minimum dispersal rate of 0.7 km/y to move successfully from one lake to the other. This rate is consistent with predicted dispersal rates for the mobile component of fish populations (50).

During the early Holocene, Lake Turkana was also connected via an overflow channel to Lakes Abaya, Chamo, and Chew Bahir in the Ethiopian Rift Valley until approximately 6.5 ka (52) (Fig. 3*A*). Nilotic species that were able to colonize these Ethiopian rift lakes via the contemporaneous Nile–Turkana connection included the elongate tigerfish (*Hydrocynus forskahlii*), for which closely related haplotypes demonstrate connectivity between Nilotic and Lake Chamo populations (53). These past dispersal events combined to produce the presently close faunal similarities between the lakes of the Northern River basin, the lakes in the southern Ethiopian Rift, Lakes Albert and Edward, and the White Nile.

Following the isolation of the lakes of the Northern River system from 8.6 ka onward (Fig. 3*B*), lowered lake levels led to increasing salinities and adverse habitat conditions. The surviving Nilotic faunal elements in the now isolated lakes began to diverge from their Nile Basin ancestors. This divergence is morphologically and genetically well exemplified in the Kenyan populations of the Nile tilapia (*Oreochromis niloticus*, Cichlidae), with distinct subspecies present in Lakes Turkana, Baringo, and Bogoria and in the Suguta River (10, 54) (*SI Appendix*, Table S1). For these populations, Fig. 3*B* suggests divergence dates of between 8.2 ka (Bogoria) and 6.5 ka (Suguta and Turkana), further suggesting that endemism and divergence developed as a result of vicariance in northern Kenyan rift lakes very recently, within just a few thousand years.

The Northern River was separated from the Southern River by the drainage divide at Gilgil, which acted as a major ichthyofaunal divide. This topographic barrier was responsible for the present divergent fish faunas between the northern and southern lakes of the Kenya Rift Valley (Fig. 1*G*), which do not have a single fish species in common (*SI Appendix*, Table S1). The Southern River with its chain of lakes remained an isolated hydrological and hence biogeographic unit due to the lack of any Holocene hydrological connectivity with adjacent drainage basins. As a result, the present-day Lakes Natron, Magadi, and Naivasha do not contain Nilotic elements but instead harbor only local endemic species (Fig. 1*G*). Together Lakes Natron and Magadi contain four species of soda tilapia (*Alcolapia* spp., Cichlidae), which are adapted to the extreme temperatures (20 to 42 °C) and very high alkalinity (pH > 10) that prevail in these shallow soda lakes (48). The four soda tilapias derive from a common freshwater ancestor (55), so the monophyly of the *Alcolapia* species flock is consistent with a merged Magadi–Natron freshwater lake between 12.4 and 8.2 ka. Species divergence and adaptation to sodic conditions must have evolved rapidly in response to mid-Holocene lake isolation, declining lake–water levels, and increasing alkalinity. Whereas these adverse habitat conditions may explain the low species richness in modern Lakes Magadi and Natron, the hydrological isolation of the Southern River drainage basin during the early Holocene had a major control on their present aquatic biodiversity by excluding colonization from distant source areas and refugia.

Apart from local adaptive radiation (7, 8, 55), modern diversity gradients in East African fish species can be attributed to the degree of past hydrological connectivity between adjacent river systems, in turn reflecting the regional topography and hydrological sensitivity to climate change. We have shown that 1) an isolated river system that had no connectivity with other river systems has the lowest species richness (i.e., the Southern River basin), 2) a river system with episodic connection to adjacent catchments in the past has medium species richness (i.e., the Northern River basin), and 3) continuous connectivity (i.e., the lakes of the Nile drainage system in the Albertine Rift) resulted in the highest species richness. Faunal exchange between adjacent drainage basins was a key process in assembling the modern fish faunas and generating diversity in East Africa while geographic isolation promoted local endemicity.

### Influence on Forest Mammal Dispersal, Distribution, and Diversity.

The early Holocene river network would have enhanced the dispersal of (semi)aquatic mammals such as the hippopotamus (*Hippopotamus amphibius*) and otters (e.g., *Aonyx capensis*, *Hydrictis maculicollis*) along the Kenya Rift and beyond, similar to that for fishes. Moreover, the permanent rivers and associated riparian habitats would have provided important ecological corridors for water-dependent terrestrial species (25). In contrast, the rivers and their terrestrial surroundings likely served as barriers for the dispersal of nonvolant forest-dependent mammal species. Today, rivers of various sizes provide the range limit for a number of larger African forest mammals such as apes (6, 56) and the giant forest hog (56). In addition, studies on monkeys across the West African Dahomey gap suggest that such limits are often the result of both large water bodies and different environments on each riverside (57). Here, we predict that the Northern and Southern Rivers, in conjunction with their relatively lower elevation (Fig. 1*G*), restricted true forest habitats in the Kenya Rift Valley to the Gilgil Corridor and, consequently, represented both a physical obstacle and an environmental barrier for eastward dispersal of terrestrial forest taxa. Our proposition is supported by the available fossil evidence (32, 58) (*SI Appendix*, Fig. S6), the size of the paleo-rivers, and the spatially restricted distribution of Guinea-Congolian mammals in East Africa today (*SI Appendix*, Fig. S2). Pollen data from the Kenya Rift Valley also favor this hypothesis, as the pollen record from Lake Naivasha (59), just south of the Gilgil Corridor, showed higher percentages of tree pollen than sites at lower elevations along the Northern (Lake Bogoria) (60) and Southern Rivers (Lake Magadi) (61) (Fig. 3*C*).

During the humid early Holocene, forests spread across East Africa (24) in concert with the expansion of the river network (Fig. 3*C*). Extensive forest cover may have facilitated the eastward dispersal of forest-dependent mammals from their Congo Basin source area. Kingdon (22, 23) postulated that presently isolated forest mammal populations in Kenya derive from past dispersal events via both a northern and southern route (Fig. 1*C*). An integration of 25 pollen records with our chronology of river flow establishes the time frame and routing of viable dispersal pathways as a test of Kingdon’s model for the late Quaternary (Fig. 3*C*).

Lowland forests expanded rapidly in the region north of Lake Victoria from 14 ka until a return to drier conditions during the Younger Dryas (∼13 to 12 ka) (49, 62). By 13 ka, Lake Victoria had reached its overflow level and the Victoria Nile started to flow (45), creating an effective dispersal barrier for terrestrial fauna (19, 20) and disrupting Kingdon’s northern route (Fig. 3*C*). Thus, an ∼1,000 y window of opportunity existed for the uninterrupted eastward dispersal of Guineo-Congolian mammals across a rainforest belt that extended from the eastern Congo Basin via the Albertine Rift north of Lake Kivu (63) to the northeastern Lake Victoria region (Fig. 3*C*). A minimum dispersal rate of 0.5 km yr^−1^ would have enabled the movement of species across this region before the Victoria Nile severed this corridor. Further eastward dispersal was possible via Mount Elgon (24), whose forests could have acted as a “stepping-stone” for the forest-dependent mammals (Fig. 3*C*). The Lake Naivasha pollen record (59) reveals the highest tree cover within the Kenya Rift in the region of the > 2,000 m high Gilgil Corridor, and nearby carbon isotope records indicate lowered montane forests at the western rift flank (64) during the early Holocene as a viable dispersal pathway, when elsewhere in the rift valley the Northern and Southern Rivers and adjacent open habitats (Fig. 3*C*) would have blocked further eastward range expansion of various forest mammal species. Although this most elevated part of the rift interior at Gilgil was an effective barrier for fish dispersal between the Northern and Southern Rivers, mammal fossils (32, 33, 58) support that it acted as a narrow gateway for the eastward dispersal of forest mammals across the Kenya Rift Valley until 7 ka, when forests retreated from this region (59) (Fig. 3*C*). This corridor can explain the presently isolated populations of several Guineo-Congolian forest mammals such as the bongo, black-fronted duiker, and giant forest hog east of the rift valley (*SI Appendix*, Fig. S2). Even though the giant forest hog is considered a good swimmer (65), extensive archaeological fieldwork along the early Holocene deposits of the Northern River close to Lake Bogoria recorded *Hylochoerus* fossils only on the western riverbank but not on the eastern side (66), which further supports the idea that the Northern River at least contributed to limiting the eastward dispersal of forest mammals. We note that it is difficult to unambiguously prove the barrier effect of past rivers, yet the available fossil evidence does not suggest that forest mammals dispersed across the Northern or Southern Rivers (67) (*SI Appendix*, Fig. S6), which thus favors the barrier hypothesis.

Butynski and de Jong (68) suggested that a 150 km long section of the Central Kenya Rift Valley provided a forest corridor for the rainforest primate potto (*Perodicticus ibeanus*), but this hypothesis is not compatible with the enlarged lakes that occupied the Central Rift and the active river system (Fig. 1*E*). However, the same authors (68) also proposed the Gilgil area as the most viable corridor due to its high elevation. As the only viable corridor, the narrowness of the Gilgil Corridor may have limited the number of mammal species that could cross the rift, making it a dispersal filter rather than a perfect corridor. We propose that this spatial constraint, together with the actively flowing rivers, contributed to the current longitudinal gradients in mammal species richness (Fig. 1*C*). Presently isolated populations of several Guineo-Congolian forest mammals known only west of the Kenya Rift—e.g., the red-tailed monkey (*Cercopithecus ascanius*), yellow-backed duiker (*Cephalophus silvicultor*), and tree pangolin (*Phataginus tricuspis*) (69)—support this idea as they likely failed to cross the rift.

While the continuity of Kingdon’s northern dispersal route (22, 23) was of relatively short duration, here we suggest an alternative southern route as a direct link between both rift arms that existed without separation by rivers (Fig. 3*C*). This route directly follows the southern catchment boundary of Lake Victoria (Fig. 3 *A* and *C*), extending from the Virunga Highlands southward along the eastern shoulder of the Albertine Rift, then eastward south of Lake Victoria toward the Ngorongoro Highlands and then northward along the western rift shoulder of the Kenya Rift to Gilgil. Pollen data indicate that this route was forested between at least 14 to 10 ka (70) (Fig. 3*C*). Several presently disjunct occurrences of forest mammal species overlap with parts of this potential dispersal route (*SI Appendix*, Fig. S2).

Accordingly, we propose that boundaries of paleo-catchments represented viable terrestrial dispersal routes because of their higher elevation and the lack of waterways on these boundaries. Consistent with this argument, the Gilgil and Virunga corridors are the most elevated sectors within the Kenya and Albertine Rifts, simultaneously representing drainage divides and past terrestrial dispersal pathways (63). Identifying paleo-catchment boundaries may therefore help in identifying Quaternary dispersal routes for terrestrial fauna in other parts of the world as well.

The eastward longitudinal decline in the species richness of forest mammals in East Africa (Fig. 1*C*) can be attributed to increasing distance from the Congo Basin source area (18, 71), competitive exclusion of immigrants by resident fauna (22), and, as shown here, the disruption of migration pathways by developing rivers during the humid early Holocene. The mid-Holocene reduction in rainfall across East Africa caused further fragmentation of forests (Fig. 3*D*), exacerbating the isolation of forest mammal populations, with possibly local extinction of small populations (*sensu* 71). While forest fragmentation may have reduced local species richness, it also likely enhanced the genetic divergence of surviving populations, as perhaps manifested in the Kenyan subspecies of potto (*P. ibeanus stockleyi*), bongo (*T. eurycerus* *isaaci*), and black-fronted duiker (*C. nigrifrons hooki*) (68, 69). Given the dramatic Holocene climate and vegetation history of East Africa, incipient speciation in these mammals may be as recent an evolutionary phenomenon as that of cichlids in the Kenyan rift lakes.

### Quaternary East African Perspectives.

The East African Plateau was uplifted between 17 and 13.5 Ma (72), while full-graben faulting of this domed plateau since at least the early Pliocene (73) formed the rift valley with its substantial north–south aligned elevational gradients (> 1,000 m/200 km; Fig. 1*G*). Given that orbital forcing (i.e., precessional cyclicity) and interglacial warming induced numerous wet episodes in East Africa during the Pleistocene (61, 74), the topographic and climatic conditions that combined to contribute to a bidirectional river flow along the rift valley existed many times throughout the Quaternary. Indeed, a drill core record of the past 1 million years from the Koora Basin (Fig. 1*E*) in the South Kenya Rift shows evidence for Pleistocene hydrological connectivity and multiple episodes of rapid lake infilling following dry intervals (47, 75), similar to the Younger Dryas–Holocene transition at Lake Siriata (*SI Appendix*, Fig. S14). Hydrological connectivity with upstream sources is suggested by dated layers of rounded pumices at ∼790 ka, 270 to 210 ka, 200 to 170 ka, and 120 to 100 ka (47), partly corresponding to periods of high orbital eccentricity and interglacial climates. Our Holocene reconstruction provides a general model for inferring the impact of river activity during such earlier humid periods and may help in understanding Pleistocene dispersal events and the faunal assemblages recorded in fossil sites of the rift.

The precondition for aquatic faunal exchange between biogeographic provinces was sufficiently wet climatic conditions that produced overflow across major drainage divides such as the boundary between the Nile and Turkana catchments. The Quaternary biogeographic history of East African fish and forest fauna can be understood as a cyclic history of immigration during recurring humid periods, followed by population isolation, genetic divergence, and possible extinction during dry periods, with subsequent recolonization. Connectivity with refugia was critical for the successful recolonization of East African lakes, as documented by molecular phylogenetic studies for the last postglacial period (76, 77). Rivers likely played a significant role in shaping the diversity and distribution of East African fauna during the Quaternary.

To test this model and to reveal past biogeographic patterns associated with river systems would require systematic spatial mapping and dating of Quaternary fluvial deposits within the Kenya Rift (78) and of their associated fossil fauna, including both aquatic (e.g., fishes) and terrestrial vertebrates (e.g., mammals with narrow ecological niches). Sufficiently long proxy records (e.g., pollen, carbon isotopes) are needed to reconstruct past ecological conditions, while both lake balance models and paleo-climate simulations with realistic representation of the East African topography could provide quantitative estimates of moisture and runoff histories. We note that even the Holocene fossil vertebrate record from the Kenya Rift is meager and that fossil surveys along the Northern and Southern River courses are vital for evaluating their significance as Holocene dispersal routes and barriers. Comparing the diversity of fossil assemblages from past arid and humid intervals should reveal whether species richness was enhanced by river activity.

## Conclusions

We have shown how the interplay of topography, climate, and hydrology governed the formation of an expanded early Holocene river system in the presently much drier Kenya Rift Valley, which in turn strongly influenced the postglacial range expansion of aquatic and terrestrial organisms. Our spatiotemporal reconstruction of East African paleo-drainage basins and associated dispersal and divergence events provide a testable a priori model for phylogeographic studies (79).

Whereas range disjunctions of freshwater fishes in coastal regions have been attributed to drainage basin connectivity during the last glacial sea-level lowstand (17, 80), we present a mechanism for drainage basin connectivity of inland regions that rests on overflow across drainage divides during wet (high-eccentricity, interglacial) periods. Moreover, while tectonism over million-year timescales has previously been invoked as a physical mechanism permitting fish dispersal between African river basins (81), we show that independent of tectonic activity, orbitally driven increases in rainfall on shorter, multimillennial timescales led to overflow of lake basins, merging of catchments, and episodic connectivity of isolated river basins. These rather short-lived hydrological connections during the early Holocene facilitated postglacial recolonization of Kenyan rift lakes and subsequent genetic and phenotypic divergence, as well as incipient speciation of the local fauna in a drier climate over the past few millennia. Climate-driven rearrangements of drainage basins thus provide an additional explanation for dispersal events and faunal exchange of African aquatic biota. African disjunctions of forest mammals also resulted from early Holocene dispersal followed by mid-Holocene vicariance. However, as barriers, rivers had an opposite influence on the dispersal success of mammals, which instead effectively moved along drainage divides.

The longitudinal gradients in vertebrate species richness across East Africa can be attributed to the differing extent and duration of past river flow and lake connectivity. Drainage basin connectivity due to lake overflow was a key mechanism for Holocene faunal exchange between Albertine and Kenyan rift lakes and for the assembly of biological diversity in the Kenya Rift. The species presently found in Kenyan rift lakes and fragmented forests, with their high degree of endemicity and adaptations, are a window into a recent past with substantial environmental fluctuations, yet their localized occurrence makes these species highly vulnerable to extinction and should encourage their conservation.

## Materials and Methods

### Spatial Analysis.

We used the TanDEM-X DEM (spatial resolution, 12 m) for visualization purposes and to model the early Holocene stream network, determine (paleo)catchments, and derive swath profiles for the entire Kenya Rift with Topo Toolbox 2 in MATLAB (82). With the ALOS World DEM (spatial resolution, 5 m), we identified locations of river gorges, quantified their dimensions (gorge widths reported represent bottom widths of gorges), visualized them for the South Kenya Rift, and also quantified the lake volume of Lake Siriata. In the Koora Basin and in the Menengai region, we used Structure-from-Motion (SfM)–based digital elevation and surface models (DSM) to detect erosive shoreline features and map outlet channels. For Menengai, we analyzed the 25 cm resolution DSM of reference (35). In the Koora Basin, we generated a 20 cm resolution DEM using aerial images of a unattended aerial system (UAS) survey (sensefly eBee classic, flown at 250 to 300 m above the ground). The SfM processing was done with Agisoft Photoscan (now Metashape), with a final filtering and vegetation removal step using LAStools (83). The ground elevation of the final point cloud was aligned to match our Differential Global Positioning System (DGPS) survey points and had an average point density of 28 pts/m^2^. All elevation data are reported with EGM96/WGS84 datums.

In the Koora, Olorgesailie, Kwenia, and Naivasha Basins, we determined absolute elevations of outcrop sections, riverbeds, and land surfaces and the width of riverbeds at high precision (decimeter-scale absolute vertical accuracy) by differential GPS measurements in February and July 2016. Point measurements at field sites were collected using a Leica Viva GS10 receiver with AS10 antenna (logging of GPS L1, L2, and L5 with carrier phase) or using a related Geomax receiver setup. At the same time, at fixed base locations, multiday global navigation satellite system (GNSS) data were logged respectively (equipment see above, maximum baseline distance 25 km). In postprocessing, our base station data were first corrected using positional data from the IGS (International GNSS service) station in Nairobi (baseline distance ~100 km, station-id "RCMN"). These processed base station data were then used for positional correction of the individual measurements from the moving receiver. All postprocessing was done with Leica Geo Office and included the application of global navigation broadcast files, satellite ephemerals, and National Oceanic and Atmospheric Administration final GPS orbits. The final point data were referenced to the EGM96/WGS84 datums. To precisely determine paleo-lake elevations, we used the dGPS elevations to align the ground altitudes of our SfM datasets to the same absolute reference frame.

### Overflow Chronology.

We collated 137 published ^14^C dates from Kenyan rift lakes from the literature and integrated them with our ^14^C chronology for Lake Siriata (*n* = 19). To constrain the timing of lake overflow, we only considered ^14^C dates from material that could be clearly related to overflow elevations (e.g., shorelines) or, alternatively, from elevations that indicated closed-basin conditions (no overflow; *SI Appendix* contains further details). Dated material obtained from sediment cores or exposed sediment profiles was utilized only when it unequivocally indicated open or closed lake conditions (e.g., diatom records; [42]). The Magadi–Natron and Baringo–Bogoria basins receive radiocarbon-depleted CO_2_ from the subsurface via hydrothermal springs (40, 84), which necessitated a reservoir correction for their chronologies. We applied a 2,050 ± 63 ^14^C-y subtraction to the Magadi–Natron ^14^C dates based on a U/Th date from Taieb et al. (85) and a 3,980 ^14^C-y subtraction to the Baringo–Bogoria ^14^C dates following De Cort et al. (86) (*SI Appendix* contains further details). For each lake basin, we calibrated all (reservoir-corrected) ^14^C dates to calendar years before present (present = AD 1950; reported as ka) with Calib 7.0.4 (87) using the IntCal13 calibration curve (88). For each lake basin, we calculated the cumulative probability distribution (CPD) from all used ^14^C dates to determine and visualize the most probable time of overflow, for which we used the 2-sigma ranges of the CPDs (*SI Appendix*, Table S6; in Fig. 2, color-coded CPDs: overflow level—dark blue, closed-basin level—light blue, and dry conditions—yellow).

### Sedimentology, Paleontology, and Geochronology.

In July 2015, February 2016, and July 2016, we mapped and sampled lacustrine and fluvial deposits in the Central and South Kenya Rift, including sites in the Olorgesailie, Siriata, Kwenia, and Naivasha basins and in the wider area of the Menengai volcanic complex. In the Siriata area, we collected bulk sediment samples from three key outcrops (designated as 1A, 3E, and 6B; *SI Appendix*, Fig. S4) at intervals of 10 to 25 cm. Samples were shipped to the Smithsonian Institution’s National Museum of Natural History (Washington, DC), where sediment was sieved and examined for charcoal, mollusk shells, and fish fossils. Fish fossils were further examined and photographed under a Leica MZ8 light microscope and with a JEOL JSM-6510 scanning electron microscope after coating with a gold–palladium alloy and identified using Tichy and Seegers (48). Shell samples of the mollusk *Corbicula* cf. *fluminalis* (*n* = 63) and the gastropod *Melanoides tuberculata* (*n* = 27) were cleaned with deionized water in an ultrasonic bath, and their δ^18^O isotopic composition was determined at the Lamont-Doherty Earth Observatory stable isotope laboratory using a Kiel IV carbonate device coupled to a Delta V isotope ratio mass spectrometer. The δ^18^O was measured on either four (*n* = 21) or three (*n* = 2) shell samples per sampling interval (SD, 0.08–1.83‰; *SI Appendix*, Table S7).

We obtained accelerator mass spectrometry (AMS) radiocarbon dates from mollusk shells, charcoal, fish bone, and sediment bulk samples (*n* = 22) collected at Siriata, Kwenia, and Olorgesailie. Samples were submitted to the Poznan Radiocarbon Laboratory for accelerator mass spectrometry, where shells were pretreated by selective acid leaching. Three sample pairs of shells and charcoal from Siriata showed significant but consistent offsets in the ^14^C ages by 3,930 ± 120 y, 3,980 ± 260 y, and 4,380 ± 120 y, indicating an average reservoir effect of 4,095 ± 180 ^14^C-y, which was subtracted from all individual shell ^14^C dates (*SI Appendix*, Table S3). We constructed Bayesian age models for two Lake Siriata outcrops with the rbacon package 2.3.8 (89; *SI Appendix*, Fig. S7). Using the age model output, we calculated medians and uncertainty envelopes with 68% and 95% confidence intervals for the Siriata δ^18^O record in MATLAB.

Sanidine phenocrysts extracted from pumice clasts sampled at two locations within the Siriata lake deposits were dated by the single-crystal incremental heating ^40^Ar/^39^Ar method (*SI Appendix* contains further details): sample OLOR16/SKG-1pB1 from the upper levels of the diatomite beds at 1.961118°S, 36.367585°E, and sample OLOR16/SKG-2p1 from 310 m to the southwest at 1.963817°S, 36.366806°E, from beach gravel where the diatomaceous deposits shoal against older trachyte (*SI Appendix*, Fig. S8). We conducted experiments on 11 and 18 phenocrysts from the two samples, respectively, and obtained ages of 12.3 ± 2.7 ka and 11.0 ± 1.0 ka (1σ uncertainty), using Bayesian eruptive-age modeling (90, 91).

### Biogeographic Analysis.

We generated geological area cladograms based on our overflow chronology (for lakes) and published radiocarbon-dated pollen records (for forests; *SI Appendix*, Table S8) to infer the sequence of Holocene connectivity and isolation of lakes and forests in East Africa. We developed maps that illustrated the pathways of aquatic and terrestrial faunal exchange for the early Holocene based on the reconstructed East African river network, past forest extent (inferred from dated pollen records), and vertebrate fossil records. Pollen data were obtained from the African Pollen Database (92), available at the Neotoma Paleoecology Database (93) (https://www.neotomadb.org, accessed 8 March 2022) or other published sources (*SI Appendix*, Table S8). To estimate early Holocene forest extent, for each pollen site we calculated the average percentage of total arboreal pollen from all pollen samples that fell in the time interval from 12 to 8 ka based on an upland pollen sum. Holocene fossil vertebrate occurrence data were obtained from various published sources (*SI Appendix*, Table S2 and Fig. S6) and our own finds. We also considered available evidence for past range connectivity from published taxonomic studies and modern range maps for freshwater fishes (class: Actinopterygii) and terrestrial mammals from the International Union for Conservation of Nature and Natural Resources (IUCN) (94) (https://www.iucnredlist.org/resources/spatial-data-download; *SI Appendix*, Table S1 and Fig. S2).

## Supplementary Material

Supplementary File

## Data Availability

All study data are included in the article and/or *SI Appendix*.

## References

[1] A. J. Plumptre , The biodiversity of the Albertine Rift. Biol. Conserv. 134, 178–194 (2007).

[2] C. N. Jenkins, S. L. Pimm, L. N. Joppa, Global patterns of terrestrial vertebrate diversity and conservation. Proc. Natl. Acad. Sci. U.S.A. 110, E2602–E2610 (2013). 23803854 10.1073/pnas.1302251110 PMC3710798

[3] R. A. Mittermeier , Hotspots Revisited: Earth’s Biologically Richest and Most Endangered Ecoregions (Cemex, 2004).

[4] E. Verheyen, W. Salzburger, J. Snoeks, A. Meyer, Origin of the superflock of cichlid fishes from Lake Victoria, East Africa. Science 300, 325–329 (2003). 12649486 10.1126/science.1080699

[5] W. Salzburger, B. Van Bocxlaer, A. Cohen, Ecology and evolution of the African Great Lakes and their faunas. Annu. Rev. Ecol. Evol. Syst. 45, 519–545 (2014).

[6] N. M. Anthony , The role of Pleistocene refugia and rivers in shaping gorilla genetic diversity in central Africa. Proc. Natl. Acad. Sci. U.S.A. 104, 20432–20436 (2007). 18077351 10.1073/pnas.0704816105 PMC2154448

[7] C. E. Wagner, L. J. Harmon, O. Seehausen, Cichlid species-area relationships are shaped by adaptive radiations that scale with area. Ecol. Lett. 17, 583–592 (2014). 24602171 10.1111/ele.12260

[8] O. Seehausen, African cichlid fish: A model system in adaptive radiation research. Proc. Biol. Sci. 273, 1987–1998 (2006). 16846905 10.1098/rspb.2006.3539 PMC1635482

[9] A. Antonelli , Amazonia is the primary source of neotropical biodiversity. Proc. Natl. Acad. Sci. U.S.A. 115, 6034–6039 (2018). 29760058 10.1073/pnas.1713819115 PMC6003360

[10] L. Seegers, L. De Vos, D. O. Okeyo, Annotated checklist of the freshwater fishes of Kenya (excluding the lacustrine haplochromines from Lake Victoria). J. East Afr. Nat. Hist. 92, 11–47 (2003).

[11] L. C. Beadle, The Inland Waters of Tropical Africa: An Introduction to Tropical Limnology (Longman, London, UK, 1974).

[12] T. R. Roberts, Geographical distribution of African freshwater fishes. Zool. J. Linn. Soc. 57, 249–319 (1975).

[13] Y. Garcin, D. Melnick, M. R. Strecker, D. Olago, J. J. Tiercelin, East African mid-Holocene wet–dry transition recorded in palaeo-shorelines of Lake Turkana, northern Kenya Rift. Earth Planet. Sci. Lett. 331, 322–334 (2012).

[14] P. deMenocal , Abrupt onset and termination of the African humid period: Rapid climate responses to gradual insolation forcing. Quat. Sci. Rev. 19, 347–361 (2000).

[15] N. A. Drake, R. M. Blench, S. J. Armitage, C. S. Bristow, K. H. White, Ancient watercourses and biogeography of the Sahara explain the peopling of the desert. Proc. Natl. Acad. Sci. U.S.A. 108, 458–462 (2011). 21187416 10.1073/pnas.1012231108 PMC3021035

[16] C. P. D. Harvey, A. T. Grove, A prehistoric source of the Nile. Geogr. J. 148, 327–336 (1982).

[17] J. Carvajal-Quintero , Drainage network position and historical connectivity explain global patterns in freshwater fishes’ range size. Proc. Natl. Acad. Sci. U.S.A. 116, 13434–13439 (2019). 31209040 10.1073/pnas.1902484116 PMC6613146

[18] W. A. Rodgers, C. F. Owen, K. M. Homewood, Biogeography of East African forest mammals. J. Biogeogr. 9, 41–54 (1982).

[19] P. Grubb, O. Sandrock, O. Kullmer, T. M. Kaiser, F. Schrenk, “Relationships between eastern and southern African mammal faunas” in African Biogeography, Climate Change, and Human Evolution, T. G. Bromage, F. Schrenk, Eds. (Oxford University Press, Oxford, UK, 1999), pp. 253–267.

[20] R. Heller, J. B. A. Okello, H. Siegismund, Can small wildlife conservancies maintain genetically stable populations of large mammals? Evidence for increased genetic drift in geographically restricted populations of Cape buffalo in East Africa. Mol. Ecol. 19, 1324–1334 (2010). 20298469 10.1111/j.1365-294X.2010.04589.x

[21] J. Kingdon , Eds., *Mammals of Africa.* *Volume I: Introductory Chapters and Afrotheria* (Bloomsbury Publishing, London, UK, 2013).

[22] J. Kingdon, Where have the colonists come from? A zoogeographical examination of some mammalian isolates in eastern Africa. Afr. J. Ecol. 19, 115–124 (1981).

[23] J. Kingdon, East African Mammals (Academic Press, London, UK, vol. I, 1971).

[24] A. C. Hamilton, Environmental History of East Africa: A Study of the Quaternary (Academic Press, London, UK, 1982).

[25] I. Rodriguez‐Iturbe, R. Muneepeerakul, E. Bertuzzo, S. A. Levin, A. Rinaldo, River networks as ecological corridors: A complex systems perspective for integrating hydrologic, geomorphologic, and ecologic dynamics. Water Resour. Res. 45, W01413 (2009).

[26] J. D. Tonkin , The role of dispersal in river network metacommunities: Patterns, processes, and pathways. Freshw. Biol. 63, 141–163 (2018).

[27] A. Rinaldo, M. Gatto, I. Rodriguez-Iturbe, River networks as ecological corridors: A coherent ecohydrological perspective. Adv. Water Resour. 112, 27–58 (2018). 29651194 10.1016/j.advwatres.2017.10.005 PMC5890385

[28] A. R. Wallace, On the monkeys of the Amazon. Ann. Mag. Nat. Hist. 14, 451–454 (1854).

[29] K. W. Butzer, G. L. Isaac, J. L. Richardson, C. Washbourn-Kamau, Radiocarbon dating of East African lake levels. Science 175, 1069–1076 (1972). 17797378 10.1126/science.175.4026.1069

[30] L. S. Sklar, W. E. Dietrich, Sediment and rock strength controls on river incision into bedrock. Geology 29, 1087–1090 (2001).

[31] L. S. Sklar, W. E. Dietrich, A mechanistic model for river incision into bedrock by saltating bed load. Water Resour. Res. 40, W06301 (2004).

[32] D. P. Gifford-Gonzalez, Faunal assemblages from Masai Gorge rockshelter and Marula rockshelter. Azania 20, 69–88 (1985).

[33] A. T. Hopwood, “Appendix C. Preliminary report on the fossil Mammalia” in The Stone Age Cultures of Kenya Colony, L. S. B. Leakey, Ed. (Cambridge University Press, London, UK, 1931), pp. 271–275.

[34] P. T. Leat, Geological evolution of the trachytic caldera volcano Menengai, Kenya Rift Valley. J. Geol. Soc. London 141, 1057–1069 (1984).

[35] S. Riedl, D. Melnick, G. K. Mibei, L. Njue, M. R. Strecker, Continental rifting at magmatic centres: Structural implications from the Late Quaternary Menengai Caldera, central Kenya Rift. J. Geol. Soc. London 177, 153–169 (2020).

[36] N. Blegen , The Menengai Tuff: A 36 ka widespread tephra and its chronological relevance to Late Pleistocene human evolution in East Africa. Quat. Sci. Rev. 152, 152–168 (2016).

[37] R. B. Owen, R. W. Renaut, The early Holocene palaeogeography of the northern Kenya Rift Valley. Rech. Géol. en Afr. 5, 130–133 (1980).

[38] Y. Garcin , Late Pleistocene–Holocene rise and collapse of Lake Suguta, northern Kenya Rift. Quat. Sci. Rev. 28, 911–925 (2009).

[39] P. H. Truckle, Geology and late Cainozoic lake sediments of the Suguta Trough, Kenya. Nature 263, 380–383 (1976).

[40] J. J. Tiercelin, A. Vincens, Le demi-graben de Baringo-Bogoria, Rift Gregory, Kenya. 30000 ans d’histoire hydrologique et sédimentaire. Bull. Cent. Rech. Explor. Prod. Elf-Aquitaine 11, 249–540 (1987).

[41] J. L. Richardson, A. E. Richardson, History of an African rift lake and its climatic implications. Ecol. Monogr. 42, 499–534 (1972).

[42] J. L. Richardson, R. A. Dussinger, Paleolimnology of mid-elevation lakes in the Kenya Rift Valley. Hydrobiologia 143, 167–174 (1986).

[43] C. Hillaire-Marcel, O. Carro, J. Casanova, ^14^C and Th/U dating of Pleistocene and Holocene stromatolites from East African paleolakes. Quat. Res. 25, 312–329 (1986).

[44] L. A. Olaka , Groundwater fluoride enrichment in an active rift setting: Central Kenya Rift case study. Sci. Total Environ. 545–546, 641–653 (2016). 10.1016/j.scitotenv.2015.11.161 26775113

[45] K. R. Beuning, K. Kelts, J. Russell, B. B. Wolfe, Reassessment of Lake Victoria–Upper Nile River paleohydrology from oxygen isotope records of lake-sediment cellulose. Geology 30, 559–562 (2002).

[46] L. G. Thompson , Kilimanjaro ice core records: Evidence of Holocene climate change in tropical Africa. Science 298, 589–593 (2002). 12386332 10.1126/science.1073198

[47] A. L. Deino , Chronostratigraphic model of a high-resolution drill core record of the past million years from the Koora Basin, south Kenya Rift: Overcoming the difficulties of variable sedimentation rate and hiatuses. Quat. Sci. Rev. 215, 213–231 (2019).

[48] H. Tichy, L. Seegers, The *Oreochromis alcalicus* flock (Teleostei Cichlidae) from lakes Natron and Magadi, Tanzania and Kenya: A model for the evolution of new species flocks in historical times. Ichthyol. Explor. Freshwat. 10, 147–174 (1999).

[49] K. R. Beuning, M. R. Talbot, K. Kelts, A revised 30,000-year paleoclimatic and paleohydrologic history of Lake Albert, East Africa. Palaeogeogr. Palaeoclimatol. Palaeoecol. 136, 259–279 (1997).

[50] J. Radinger, C. Wolter, Patterns and predictors of fish dispersal in rivers. Fish Fish. 15, 456–473 (2014).

[51] G. Fryer, T. D. Iles, The Cichlid Fishes of the Great Lakes of Africa: Their Biology and Evolution (Oliver & Boyd, Edinburgh, UK, 1972).

[52] A. T. Grove, F. A. Street, A. S. Goudie, Former lake levels and climatic change in the rift valley of Southern Ethiopia. Geogr. J. 141, 177–194 (1975).

[53] S. A. Goodier, F. P. Cotterill, C. O’Ryan, P. H. Skelton, M. J. de Wit, Cryptic diversity of African tigerfish (genus *Hydrocynus*) reveals palaeogeographic signatures of linked neogene geotectonic events. PLoS One 6, e28775 (2011). 22194910 10.1371/journal.pone.0028775 PMC3237550

[54] T. C. Ndiwa, D. W. Nyingi, J. F. Agnese, An important natural genetic resource of *Oreochromis niloticus* (Linnaeus, 1758) threatened by aquaculture activities in Loboi drainage, Kenya. PLoS One 9, e106972 (2014). 25222491 10.1371/journal.pone.0106972 PMC4164595

[55] A. G. Ford , High levels of interspecific gene flow in an endemic cichlid fish adaptive radiation from an extreme lake environment. Mol. Ecol. 24, 3421–3440 (2015). 25997156 10.1111/mec.13247 PMC4973668

[56] D. Livingstone, J. Kingdon, “The evolution of a continent: Geography and geology” in Mammals of Africa. Volume 1: Introductory Chapters and Afrotheria, J. Kingdon , Eds. (Bloomsbury Publishing, London, UK, 2013), pp. 27–42.

[57] A. H. Harcourt, M. A. Wood, Rivers as barriers to primate distributions in Africa. Int. J. Primatol. 33, 168–183 (2012).

[58] S. H. Ambrose, Excavations at Masai Gorge Rockshelter, Naivasha. Azania 20, 29–67 (1985).

[59] J. M. Maitima, Vegetation response to climatic change in Central Rift Valley, Kenya. Quat. Res. 35, 234–245 (1991).

[60] A. Vincens, Diagramme pollinique d’un sondage Pleistocene superieur—Holocene du Lac Bogoria (Kenya). Rev. Palaeobot. Palynol. 47, 169–192 (1986).

[61] R. B. Owen , Progressive aridification in East Africa over the last half million years and implications for human evolution. Proc. Natl. Acad. Sci. U.S.A. 115, 11174–11179 (2018). 30297412 10.1073/pnas.1801357115 PMC6217406

[62] R. L. Kendall, An ecological history of the Lake Victoria basin. Ecol. Monogr. 39, 121–176 (1969).

[63] M. W. Tocheri , The evolutionary origin and population history of the Grauer gorilla. Am. J. Phys. Anthropol. 159, S4–S18 (2016). 26808111 10.1002/ajpa.22900

[64] S. H. Ambrose, M. J. DeNiro, Climate and habitat reconstruction using stable carbon and nitrogen isotope ratios of collagen in prehistoric herbivore teeth from Kenya. Quat. Res. 31, 407–422 (1989).

[65] R. Reyna‐Hurtado, J. P. d’Huart, A. K. Turkalo, “Forest hog *Hylochoerus meinertzhageni* (Thomas 1904)” in Ecology, Conservation and Management of Wild Pigs and Peccaries, M. Melletti, E. Meijaard, Eds. (Cambridge University Press, Cambridge, UK, 2017), pp. 114–121.

[66] W. R. Farrand, R. R. Redding, M. H. Wolpoff, H. T. Wright III, An Archaeological Investigation on the Loboi Plain, Baringo District, Kenya. Research Reports in Archeology Contribution 1. Museum of Anthropology (University of Michigan, Ann Arbor, MI, 1976).

[67] H. Jousse, Atlas of Mammal Distribution Through Africa From the LGM (∼18 ka) to Modern Times (Archaeopress Publishing Ltd., Oxford, UK, 2017) 6.

[68] T. M. Butynski, Y. A. de Jong, Distribution of the potto *Perodicticus potto* (Primates: Lorisidae) in eastern Africa, with a description of a new subspecies from Mount Kenya. J. East Afr. Nat. Hist. 96, 113–147 (2007).

[69] J. Kingdon, The Kingdon Field Guide to African Mammals (Princeton University Press, Princeton, NJ, ed. 2, 2015).

[70] M. A. Ryner, R. Bonnefille, K. Holmgren, A. Muzuka, Vegetation changes in Empakaai Crater, northern Tanzania, at 14,800-9300 cal yr BP. Rev. Palaeobot. Palynol. 140, 163–174 (2006).

[71] R. H. MacArthur, E. O. Wilson, The Theory of Island Biogeography (Princeton University Press, Princeton, NJ, 1967).

[72] H. Wichura , A 17-my-old whale constrains onset of uplift and climate change in east Africa. Proc. Natl. Acad. Sci. U.S.A. 112, 3910–3915 (2015). 25775586 10.1073/pnas.1421502112 PMC4386349

[73] B. H. Baker, J. G. Mitchell, L. A. J. Williams, Stratigraphy, geochronology and volcano-tectonic evolution of the Kedong–Naivasha–Kinangop region, Gregory Rift Valley, Kenya. J. Geol. Soc. London 145, 107–116 (1988).

[74] J. E. Kutzbach , African climate response to orbital and glacial forcing in 140,000-y simulation with implications for early modern human environments. Proc. Natl. Acad. Sci. U.S.A. 117, 2255–2264 (2020). 31964850 10.1073/pnas.1917673117 PMC7007574

[75] R. Potts , Increased ecological resource variability during a critical transition in hominin evolution. Sci. Adv. 6, eabc8975 (2020). 33087353 10.1126/sciadv.abc8975 PMC7577727

[76] K. R. Elmer , Pleistocene desiccation in East Africa bottlenecked but did not extirpate the adaptive radiation of Lake Victoria haplochromine cichlid fishes. Proc. Natl. Acad. Sci. U.S.A. 106, 13404–13409 (2009). 19651614 10.1073/pnas.0902299106 PMC2726394

[77] O. Seehausen, Patterns in fish radiation are compatible with Pleistocene desiccation of Lake Victoria and 14,600 year history for its cichlid species flock. Proc. Biol. Sci. 269, 491–497 (2002). 11886641 10.1098/rspb.2001.1906 PMC1690916

[78] A. K. Behrensmeyer, R. Potts, A. Deino, The Oltulelei Formation of the southern Kenyan Rift Valley: A chronicle of rapid landscape transformation over the last 500 ky. Geol. Soc. Am. Bull. 130, 1474–1492 (2018).

[79] L. L. Knowles, W. P. Maddison, Statistical phylogeography. Mol. Ecol. 11, 2623–2635 (2002). 12453245 10.1046/j.1365-294x.2002.01637.x

[80] M. de Bruyn , Paleo-drainage basin connectivity predicts evolutionary relationships across three Southeast Asian biodiversity hotspots. Syst. Biol. 62, 398–410 (2013). 23391942 10.1093/sysbio/syt007

[81] J. Schwarzer , Repeated trans-watershed hybridization among haplochromine cichlids (Cichlidae) was triggered by Neogene landscape evolution. Proc. Biol. Sci. 279, 4389–4398 (2012). 22951733 10.1098/rspb.2012.1667 PMC3479809

[82] W. D. Schwanghart, D. Scherler, TopoToolbox 2–MATLAB-based software for topographic analysis and modeling in Earth surface sciences. Earth Surf. Dyn. 2, 1–7 (2014).

[83] M. Isenburg, rapidlasso LAStools (Version 160910, Gilching, Germany, 2016). https://rapidlasso.com/LAStools. Accessed 1 October 2016.

[84] H. Lee , Massive and prolonged deep carbon emissions associated with continental rifting. Nat. Geosci. 9, 145–149 (2016).

[85] M. Taieb, P. Barker, A. Vincens, D. Williamson, R. Bonnefille, Histoire paléohydrologique du lac Magadi (Kenya) au Pleistocène superieur. C. R. Acad. Sci. II 313, 339–346 (1991).

[86] G. De Cort , Late-Holocene and recent hydroclimatic variability in the central Kenya Rift Valley: The sediment record of hypersaline lakes Bogoria, Nakuru and Elementeita. Palaeogeogr. Palaeoclimatol. Palaeoecol. 388, 69–80 (2013).

[87] M. Stuiver, P. J. Reimer, Extended ^14^C data base and revised CALIB 3.0 ^14^C age calibration program. Radiocarbon 35, 215–230 (1993).

[88] P. J. Reimer , IntCal13 and Marine13 radiocarbon age calibration curves 0–50,000 years cal BP. Radiocarbon 55, 1869–1887 (2013).

[89] M. Blaauw, J. A. Christen, Flexible paleoclimate age-depth models using an autoregressive gamma process. Bayesian Anal. 6, 457–474 (2011).

[90] C. B. Keller, B. Schoen, K. M. Samperton, A stochastic sampling approach to zircon eruption age interpretation. Geochem. Perspect. Lett. 8, 31–35 (2018).

[91] A. L. Deino , Chronostratigraphy of the Baringo-Tugen Hills-Barsemoi (HSPDP-BTB13-1A) core—^40^Ar/^39^Ar dating, magnetostratigraphy, tephrostratigraphy, sequence stratigraphy and Bayesian age modeling. Palaeogeogr. Palaeoclimatol. Palaeoecol. 570, 109519 (2021).

[92] S. Ivory, A. M. Lézine, E. Grimm, J. W. Williams, Relaunching the African pollen database: Abrupt change in climate and ecosystems. PAGES Mag. 28, 26–35 (2020).

[93] J. W. Williams , The neotoma paleoecology database: A multi-proxy, international community-curated data resource. Quat. Res. 89, 156–177 (2018).

[94] International Union for Conservation of Nature and Natural Resources, The IUCN Red List of Threatened Species, version 2020-3. https://www.iucnredlist.org. Accessed 11 March 2020.

