# Supplementary material for: Holocene bidirectional river system along the Kenya Rift and its influence on East African faunal exchange and diversity gradients
Source: Proc Natl Acad Sci U S A. 2022 Jun 27;119(28):e2121388119. doi: 10.1073/pnas.2121388119 (PMC9282390; doi:10.1073/pnas.2121388119)
Supplement: Supplementary File [file pnas.2121388119.sapp.pdf]

PNAS
www.pnas.org

## Supplementary Information for

# Holocene bidirectional river system along the Kenya Rift and its influence on East African faunal exchange and diversity gradients

René Dommain[1,2,3], Simon Riedl[1], Lydia A. Olaka[4,5], Peter deMenocal[6,7], Alan Deino[8], R. Bernhart Owen[9], Veronica Muiruri[10], Johannes Müller[11], Richard Potts[2,10], Manfred R. Strecker[1]

[1] Institute of Geosciences, University of Potsdam, 14476 Potsdam-Golm, Germany
[2] Human Origins Program, National Museum of Natural History, Smithsonian Institution, Washington, DC 20013, USA
[3] Asian School of the Environment, Nanyang Technological University, 639798, Singapore, Singapore
[4] Department of Earth and Climate Science, University of Nairobi, P.O. Box 30197, 00100, Nairobi, Kenya
[5] Institute for Climate Change and Adaptation (ICCA), University of Nairobi, P.O. Box 30197, 00100, Nairobi, Kenya
[6] Woods Hole Oceanographic Institution, Woods Hole, MA 02543, USA
[7] Lamont-Doherty Earth Observatory, Division of Biology and Paleo Environment, Palisades, NY 10964, USA
[8] Berkeley Geochronology Center, Berkeley, CA 94709, USA
[9] Department of Geography, Hong Kong Baptist University, Kowloon Tong, Hong Kong
[10] Department of Earth Sciences, National Museums of Kenya, Nairobi 00100, Kenya
[11] Museum für Naturkunde, Leibniz-Institut für Evolutions- und Biodiversitätsforschung, 10115 Berlin, Germany

Corresponding author: René Dommain
Email: rene.dommain@ntu.edu.sg

**This PDF file includes:**

Supplementary text S1–S3
Figures S1 to S14
Tables S1 to S8
SI References

## S1. Detailed Methods

### S1.1. Radiocarbon reservoir correction

Previous studies have noted significant disagreement in the timing of highstands in East African lakes during the African Humid Period (e.g., refs. 1, 2, 3, 4). We note that it was only possible to establish the synchronicity of overflow lake levels and the connectivity between all major lake basins by applying $^{14}C$ reservoir corrections to the chronologies of Lakes Magadi-Natron, Siriata, Baringo-Bogoria and Suguta. Reservoir-affected $^{14}C$ dates have significantly older ages of the order of 2000 – 4000 years than ages derived from reservoir-free $^{14}C$ dates, U/Th dates, or $^{137}Cs$ dates, which now constrain the reservoir effects for these basins (e.g., refs. 5, 6). The source of depleted radiocarbon can be attributed to the continual release of mantle-derived $CO_2$ along faults and from hot springs in the tectonically active rift-floor area (7). Whether or not Nakuru-Elmenteita, Naivasha and Menengai sediments also contain this source of chronological error (which is apparently negligible in Lake Turkana; 8) remains to be investigated.

Lake Suguta

A reservoir effect of Lake Suguta was first investigated by Junginger et al. (9), who derived a mean reservoir factor of 1900 $^{14}C$ years based on age offsets of four pairs of charcoal and carbonate dates (1570, 1940, 1970 and 2240 $^{14}C$ years). Garcin et al. (6) showed that the mean reservoir effect has a large error (1σ= 275 $^{14}C$ years), indicating that the reservoir effect likely changed over time. These authors (6) therefore established a reservoir model and applied following reservoir factors: 1570±80 $^{14}C$ years for dates ≥10930±50 $^{14}C$ BP, 2240±65 $^{14}C$ years for dates from 10795±50 to 10205±45 $^{14}C$ BP, 1970±70 $^{14}C$ years for dates from 10025±45 to 7850±50 $^{14}C$ BP and 1940±65 $^{14}C$ years for ages ≤6345±40 $^{14}C$ BP. We followed this approach for correcting carbonate-derived radiocarbon dates from Lake Suguta.

Lakes Baringo Bogoria

Both Lake Baringo and Lake Bogoria receive groundwater from on- and offshore hydrothermal springs, partly from deep sources (10, 11, 12). $CO_2$ is the dominating free-phase gas released by many of these springs that mixes with the lake water of Baringo. The $CO_2$ from hot springs of the northern Kenya Rift (including Lake Baringo) has a mean $\delta^{13}C$-$CO_2$ value of -9.2‰ (-5.4‰ — -14.6‰; n= 7; ref. 10), which is very similar to the carbon isotope composition of $CO_2$ of the Magadi-Natron springs (mean -8.0‰; ref. 7) and strongly indicates to be derived from the upper mantle (13). Hence a substantial $^{14}C$ reservoir effect for Lakes Baringo and Bogoria can be assumed and was already suspected by (1), (2) and (14) based on conspicuously old radiocarbon ages. An indication of a reservoir effect is given by two published $^{14}C$ dates obtained from the same interval (128-131 cm) of core Bogoria I for which bulk organic matter is dated to 4790±280 $^{14}C$ yr BP but the carbonate fraction to 10400±470 $^{14}C$ yr BP (difference of 5610 $^{14}C$ years) (14). Because organic matter could also have been influenced by $^{14}C$ depleted carbon the age difference does not provide an unequivocal reservoir factor. De Cort et al. (5) estimated the modern reservoir effect for Lake Bogoria from a $^{14}C$ date of 4160±35 $^{14}C$ yr BP at 40.5 cm depth and the $^{137}Cs$ activity (1964 peak at 35 cm) in core BOG01-1P. The $^{137}Cs$-based extrapolated age at 40.5 cm is 1943 (or 182 $^{14}C$ yr BP) and the deduced $^{14}C$ reservoir effect is 3980 $^{14}C$ years (5), which we applied to all radiocarbon dates from Lakes Baringo and Bogoria (given the direct overflow of Lake Bogoria waters into Lake Baringo during the early Holocene).

Lake Siriata

We investigated the $^{14}C$ reservoir effect of Lake Siriata by comparing radiocarbon dates from three sample pairs consisting of charcoal and biogenic carbonates (i.e. *Corbicula* sp. shells); each pair collected from the same depth interval of lacustrine sediment deposits (Table S3). Two shell dates

(Poz-78499, Poz-78500) collected 20 cm apart from the same outcrop (KOO15-6B) are chronologically reversed (13600±70 $^{14}C$ BP vs. 13240±70 $^{14}C$ BP), while the corresponding charcoal dates (9220±100 $^{14}C$ BP vs. 9310±100 $^{14}C$ BP) are in chronological order resulting in significantly different reservoir effects (4380±120 $^{14}C$ years vs. 3930±120 $^{14}C$ years). These differences indicate that the reservoir effect was fluctuating on short (centennial) timescales, but the temporal overlap of the two charcoal dates precludes us from resolving the time when the reservoir effect had changed. A third reservoir factor has a comparable duration (3980±120 $^{14}C$ years) but was obtained from ca. 600-year younger sediments from a different outcrop (KOO15-3E). Given the consistent magnitudes of the age offsets we applied an average reservoir factor of 4095±180 $^{14}C$ years, which was subtracted from all carbonate $^{14}C$ dates (Table S3). We also applied this reservoir effect to a $^{14}C$ date of fish bones (Poz-89660) from adjacent Lake Kwenia in the absence of a local reservoir factor (Table S3).

Magadi-Natron

Both Lakes Magadi and Natron are fed by numerous hot springs, aligned along normal faults, from which mantle-derived $CO_2$ degasses (7). Given the deep and magmatic origin of this $CO_2$ it can be safely assumed to be depleted in $^{14}C$ ('dead carbon'). The constant recharge of fluids rich in mantle-derived $CO_2$ into Lakes Magadi and Natron necessitates the correction of radiocarbon dates by a local reservoir factor. However, for these lakes no parallel radiocarbon dates on lacustrine and terrestrial matter are available to infer a reservoir effect in the standard way. To establish a $^{14}C$ reservoir effect for the Magadi-Natron Basin we compared U/Th and $^{14}C$ dates from core NF1 from Lake Magadi published by Taieb et al. (15) and Roberts et al. (16). Two U/Th dates were obtained from the depth interval 148-168 cm of core NF1: 1) 10970±750 yr BP (range: 10220-11720 yr BP) on organo-phosphates and 2) 8500±2000 yr BP on sodium-silicates (15). We discarded the second (younger) U/Th date because of its very large error, which would result in a highly uncertain temporal duration of the reservoir effect (~±2000 years).

The mean value of the 10970±750 yr BP U/Th date (= 10970 calendar years BP) is equivalent to 9560±16 $^{14}C$ yr BP on the IntCal13 radiocarbon calibration curve (17). The lower bound U/Th date of 10220 BP equals 9031±14 $^{14}C$ yr BP and the upper bound U/Th date of 11720 BP equals 10102±20 $^{14}C$ yr BP on the IntCal13 curve. Incorporating all sigma ranges yields an equivalent radiocarbon age of 9560±550 $^{14}C$ yr BP for this U/Th date (for error propagation we calculated the square root of the sum of squares of both the $^{14}C$ error and the reservoir error).

Three radiocarbon dates were obtained by Taieb et al. (15) and Roberts et al. (16) on total organic matter from intervals adjacent to or partly overlapping with the U/Th sample of Magadi core NF1. The radiocarbon dates are 12200±357 $^{14}C$ yr BP from 168-178 cm (conventional date, sample no. NF173, lab no. LGQ 134; ref. 15), 11110±110 $^{14}C$ yr BP from 139-140 cm (AMS date, lab no. OxA-3284; ref. 16) and 11420±110 $^{14}C$ yr BP from 167-168 cm (AMS date, lab no. OxA 3285; ref. 16). Subtracting the $^{14}C$ age of 9560±550 BP from these three radiocarbon dates yields following $^{14}C$ reservoir factors:

a) 12200±357 $^{14}C$ yr BP - 9560±550 $^{14}C$ yr BP = 2640±655 $^{14}C$ years (0-10 cm below the U/Th date)

b) 11110±110 $^{14}C$ yr BP - 9560±550 $^{14}C$ yr BP = 1550±560 $^{14}C$ years (8-9 cm above U/Th date)

c) 11420±110 $^{14}C$ yr BP - 9560±550 $^{14}C$ yr BP = 1860±560 $^{14}C$ years (overlapping with the lowermost 1 cm of the U/Th date).

These three reservoir factors overlap from 1985 – 2110 $^{14}C$ years. The mean of this overlapping range is ~2050±63 $^{14}C$ years. We adopt this approximation of 2050±63 $^{14}C$ years for the reservoir correction of bulk and carbonate-based $^{14}C$ dates from Lake Magadi-Natron. We note that this reservoir factor is probably a conservative estimate as the second U/Th date from core NF1 would

yield an average reservoir factor almost twice as large (~3870 $^{14}C$ years), which is similar to the 4095±180 $^{14}C$ year reservoir effect of the adjacent Lake Siriata.

## S1.2. Selection of radiocarbon dates for deriving overflow ages

All published radiocarbon dates used in this study are listed in Table S2. In most cases, we reconstructed overflow conditions from radiocarbon samples that were deposited at overflow elevations. Our analysis therefore required good documentation of sample elevations of published radiocarbon dates. It is possible that nearly identical radiocarbon dates (overlapping sigma ranges) had been obtained from the same lake at both an overflow elevation (e.g. in a (near) shoreline setting) and an elevation below overflow level (indicating closed basin conditions). In such ambiguous case we gave priority to the higher-elevation sample as it is hard to explain how a sample could have been deposited above a paleo-lake surface, but conceivable that material could have concurrently been deposited below the lake surface (i.e. lake bottom) as well. We therefore attempted to restrict the selection of radiocarbon dated samples to those representing (near) shoreline conditions and wherever possible avoided samples collected from (thick) stratigraphic sections or sediment cores as these mostly stem from deposition under a deeper water column. However, if sediment proxies (e.g. diatoms) from such sections/cores clearly indicated open (overflow) conditions we considered them as well.

Lake Turkana
Garcin et al. (8) provided a comprehensive and rigorous synthesis of published and new radiocarbon dates from the Lake Turkana Basin and established a robust lake level reconstruction with 105 $^{14}C$ dates and their corresponding sample elevations. Here we used only those dates (n=37) listed in ref. (8) which were obtained from sample locations that were at or close to the former land-water-interface of Lake Turkana (i.e. near-shore, shoreline, and beach deposits), thus clearly indicating former lake levels. We excluded dated samples from benthic sediments and those with equivocal sample context (e.g. sands, sandy silt, lacustrine deposition, archeological excavation). The reported elevations for the selected radiocarbon dates were used to determine the timing of overflow above the outlet sill located at an elevation of 457-460 m. However, it should be noted that extensional tectonism and associated normal faulting resulted in subsidence of the central Turkana Basin floor and flexural uplift of the basin margins creating substantial relief differences during the Holocene and hence diverse elevations for the maximum highstand shorelines (MHS) across, at least, the southern Turkana Basin (8, 18). Garcin et al. (8) corrected their sample elevations for subsidence, which are used here. Further radiocarbon dated stratigraphic sections and sediment cores from Lake Turkana are published by (19, 20, 21, 22, 23). Yet these studies do not add definite evidence for additional overflow periods than those based on the Garcin et al. (8) reconstruction and several of the dated samples remain uncertain in terms of their absolute elevations at the time of deposition due to the deformation of the Turkana Basin.

Lake Suguta
Lake level fluctuations of Lake Suguta were reconstructed by Bishop (24), Truckle (25), Casanova et al. (26), Garcin et al. (4, 6) and Junginger et al. (9), together reporting 68 radiocarbon dates from the Suguta valley. The reconstruction of past lake levels in the Suguta valley is complicated by Holocene crustal deformation (subsidence due to extension, normal faulting, tilting of blocks and isostatic rebound) which resulted in different absolute elevations of the maximum highstand shoreline (MHS) across the Suguta valley and in lower elevations of all MHS (≤567 m) expressions than the present elevation of the outlet sill (581 m; ref. 18). For reconstructing overflow conditions, we selected only dates (n=14) from refs. (4) and (9) which indicated deposition close to the former land-water interface (shoreline). Various sample sites of the Suguta valley are steep-sided volcanic cones from which lake deposits could have been transported or eroded to lower elevations after deposition (especially with desiccation of the basin). We therefore followed a very conservative approach when selecting radiocarbon dated samples for this basin. We also did not include samples from (within) thick stratigraphic sections given the uncertainty of relating them to past lake surface elevations and avoided the use of published $^{14}C$ dates from older publications as these

often lacked sufficient information regarding sample elevations. Yet, several $^{14}C$ dates from this earlier work (24, 25, 26) from stratigraphic sections with embedded fish fossils (Oreochromis, Lates etc.) were considered for the documentation of past fish dispersal.

Lakes Baringo and Bogoria
Lake Bogoria is surrounded by a series of shorelines (including stromatolitic deposits) between 990.7 m and 999 m (2), which is 10 m above the modern lake level (989 m in 2003; ref. 5) and represents the overflow elevation of this lake (Loboi Sill: drainage divide between Bogoria and Baringo). Due to its narrow half-graben-shape Lake Bogoria can fluctuate by several meters over subdecadal timescales (5, 27). For example, it rose to up to 996 m around 1900, ~997 m in 1928, 994 m in 1979 (2, 27) and to over 992 m in 2012 (5). Therefore, stromatolithic paleo-shoreline indicators at these elevations could have been influenced by post-depositional alteration during the past few centuries. Stromatolites occur at 999 m (dated to 4140±60 $^{14}C$ BP) and at 995 m (dated to 3880±60 and 3750±180 $^{14}C$ BP; ref. 2, 28). Given the substantial reservoir effect in Lake Bogoria the stromatolites could potentially have formed more recently than their Mid-Late Holocene ages suggest or, alternatively, could have diagenetically incorporated younger carbon during subaerial exposure (27). Because of these chronological uncertainties we avoided the use of radiocarbon dated stromatolites from Lake Bogoria. Although sediment cores from Lakes Bogoria and Baringo indicate freshwater conditions for the early Holocene (2,14), they either contain multiple age reversals likely related to the reservoir effect, lack sufficient age control or do not cover the entire Holocene (2, 5, 14). We therefore also omitted dated sediment cores from these lakes in this study. Consequently, we restricted the reconstruction of overflow to radiocarbon dated mollusk shells deposited on paleo-shorelines from Baringo, Bogoria and the intervening Loboi Plain.

Lake Menengai
Lake sediments deposited up to an elevation of 1860 m and thus indicating overflow are present in the eastern part of the Menengai Caldera (29), but this outcrop remains to be dated. We therefore used the only available radiocarbon dates from other lacustrine deposits inside the Menengai Caldera (30).

Lake Nakuru-Elmenteita
We used radiocarbon dates from sediment cores of modern Lakes Nakuru and Elmenteita, for which detailed diatom records indicate the sequence of open (overflow conditions) and closed basin conditions (31, 32). We integrated the core dates with radiocarbon dates on former shorelines and dates from sediment strata from various archeological investigations in the basin that both constrain past lake level fluctuations given their elevations with respect to the overflow level.

Lake Naivasha
Shorelines of the Lake Naivasha Basin have not been dated yet and only one onshore date is available that directly indicates a past lake level (33). We therefore relied on two dated sediment cores that cover the Holocene (32, 34). Detailed diatom records are available from these core studies, from which closed and open (fresh) conditions were reconstructed by Richardson and Richardson (34) and Richardson and Dussinger (32).

Lake Siriata
We dated lake sediments of Lake Siriata that in one case overlay a dated paleosol, which indicates dry conditions prior to the onset of the Holocene. All sediment packages can be related to a single shoreline at the overflow elevation directly dated by $^{40}Ar/^{39}Ar$ of beach pumices to the early Holocene (see below). The lack of regressive shorelines indicates the rapid desiccation of the lake basin.

Lake Magadi-Natron
Absolute lake levels were reconstructed by Hillaire-Marcel et al. (35) from dated stromatolites surrounding the entire Magadi-Natron Basin, which was fully adopted here. We did not consider sediment core studies (e.g., ref. 15, 16) due to uncertainties in translating sediment proxy data into absolute lake levels for this endorheic basin. The local reservoir effect also necessitates a

reassessment of original core data interpretations such as the proxy-based lake level reconstruction by Roberts et al. (16). Applying the reservoir effect of 2050 years to their reconstruction would place the onset of maximum lake levels to the early Holocene instead of the originally proposed Late Glacial period (a similar temporal adjustment was made for the Suguta record of Garcin et al. (4) after a $^{14}C$ reservoir-adjustment by (6) and (9)). This reservoir effect-corrected lake level reconstruction would then be synchronous with the highstand timing based on the dated Magadi-Natron stromatolites. We also incorporated radiocarbon dated layers of fossil fish from outcrop sections of the High Magadi Beds (e.g., ref. 36) for the reconstruction of the Holocene fish fauna of this basin.

### S1.3. $^{40}Ar/^{39}Ar$ dating

Sanidine phenocrysts extracted from rhyolitic pumice clasts from two levels of the Siriata lake deposits were dated by the single-crystal incremental heating $^{40}Ar/^{39}Ar$ method. Sample OLOR16/SKG-1pB1 is a single large pumice clast from the uppermost levels of the diatomite deposits (1.961118°S, 36.367585°E), while sample OLOR16/SKG-2p1 (1.963817°S, 36.366806°E) is also a single large pumice clast from beach gravel deposits 310 m southwest of the previous location, where diatomaceous sediments shoal against the older trachyte rift flanks (Fig. S8). The stratigraphic relationship between the two samples is not known, but given the unusual occurrence of large floated pumice, and similar textural and compositional appearance of pumice from the two sites (white, satiny, stretched, crystal-poor rounded to sub-rounded lapilli to blocks), they may be derived from the same eruptive event.

The methods used here are similar to those described in Deino et al. (37, 38). Samples were processed at the Berkeley Geochronology Center in preparation for $^{40}Ar/^{39}Ar$ dating, using conventional separation techniques including disaggregation with a ceramic mortar and pestle, sieving, removal of magnetite with a hand magnet, distilled water rinses, magnetic separations with a Frantz Isodynamic Separator, heavy liquid separations, and rinses in dilute HF and distilled water. Finally, inclusion-free feldspar phenocrysts were hand-picked under a binocular microscope.

The final crystal concentrates were irradiated in the Cd-lined CLICIT position of the Oregon State University TRIGA reactor for five minutes. Sanidine phenocrysts from the Alder Creek Rhyolite of California (orbitally referenced age = 1.1848 ± 0.0006 Ma; ref. 39) was employed as the neutron fluence monitor mineral. Standards and unknowns were co-irradiated in a circular configuration in wells in an aluminum disk, with standards at the cardinal positions, with two unknowns situated between standards. The appropriate neutron fluence factors (the '*J*' parameter of $^{40}Ar/^{39}Ar$ dating calculations; ref. (40)) for the unknown positions were calculated from a planar fit of the standard calibrations, with 1σ errors derived by Monte Carlo simulation in the predicted *J* value ranging from 0.1–0.3%. Reactor-induced isotopic production ratios for these irradiations were: $(36Ar/37Ar)_{Ca}$ = 3.65 ± 0.02 × 10-4, $(^{38}Ar/^{37}Ar)_{Ca}$ = 1.96 ± 0.08 × 10-5, $(^{39}Ar/^{37}Ar)_{Ca}$ = 6.95 ± 0.09 × 10-4, $(^{37}Ar/^{39}Ar)_{K}$ = 3.24 ± 0.16 × 10-4, $(^{38}Ar/^{39}Ar)_{K}$ = 1.220 ± 0.003 × 10-2, $(^{40}Ar/^{39}Ar)_{K}$ = 3.5 ± 0.9 × 10-4. Atmospheric $^{40}Ar/^{36}Ar$ = 298.56 ± 0.31 (41) and decay constants follow (42).

After a period of several months to permit radiological 'cooling' after irradiation, the individual sanidine phenocrysts were analyzed using incremental heating. Here, heating levels (laser output levels) were raised progressively from low temperature to fusion in a series of sequential independently measured steps (4-7 steps), termed the single-crystal incremental heating ('SCIH') approach. All argon measurements were carried out using an automated extraction line inlet to a Nu Instruments 5-collector Noblesse mass spectrometer, using ion-counting electronics.

Completed incremental heating analyses were checked for apparent age plateaus, using a modified approach to that of Fleck et al. (43). Here, we search for a set of contiguous steps encompassing the greatest percent of $^{39}Ar$ release that exhibit an acceptable MSWD ('mean square of weighted deviates,' with a threshold probability >95% that the observed scatter is caused by analytical error alone and that geological scatter is not demonstrated). A plateau must comprise at least 50% of the total $^{39}Ar$ release and consist of at least three consecutive steps. We add here an additional threshold for inclusion of a plateau in further data analysis, in that the overall experiment must consist of more than three steps.

The incremental heating plateau age populations for each grain were then examined using 'inverse isochron' regressions ($^{36}Ar/^{40}Ar$ vs. $^{39}Ar/^{40}Ar$), and finally the age-probability distribution of the isochron ages was examined for each of the samples. We present two approaches to calculating the central tendency and error of the sample age populations. The first calculates a conventional weighted mean, with the error as a modified standard error ('mse,' the standard error multiplied by root MSWD where MSWD > 1). The second uses a Bayesian parameter estimation approach (38, 44) that accommodates a tailing toward older ages commonly observed in East African feldspar phenocryst $^{40}Ar/^{39}Ar$ single-crystal data sets from tuffs (38).

## S2. Supplementary Results

### S2.1. $^{40}Ar/^{39}Ar$ dating

We analyzed a total of 559 incremental heating steps on 11 grains of feldspar from sample OLOR16/SKG-1pB1, and 18 grains from OLOR16/SKG-2p1 (Table S4; Fig. S9). All but two grains (both from OLOR16/SKG-1p) yielded SCIH age plateaus (Table S5). Isotope correlation diagrams (isochrons) are shown for the plateau steps from each grain in Figure S10, and results tabulated in Table S5. None of the experiments demonstrated a 'trapped' $^{40}Ar/^{36}Ar$ significantly above atmospheric composition, suggesting that an 'excess argon' component is not present in these samples.
The age-population density spectra of the isochron ages are shown in Figures S11 and S12. While sample OLOR16/SKG-2p1 displays a simple unimodal population with one marginally older result, sample OLOR16/SKG-1p exhibits a tail of older ages comprised of three experiments out of nine. Using a robust outlier detection method based on deviations from the median ('normalized median absolute deviation' >= 2; ref. 37, 38), two grains in the older tail of OLOR16/SKG-1p are omitted, and none from OLOR16/SKG-2p1. The weighted-mean ages of the retained populations are 16.2 ± 1.2 ka (1σ mse, $n$ = 7, MSWD = 1.3) for OLOR16/SKG-1p, and 11.9 ± 0.6 ka ($n$ = 18, MSWD = 1.0) for OLOR16/SKG-2p1. However, given the tailing of ages present in OLOR16/SKG-1p, we prefer an alternative method to identifying the age of these samples, using Bayesian eruptive-age modeling (38, 44). This approach, which allows use of the entire isochron data set without deletions, yields a markedly younger result for OLOR16/SKG-1p at 12.3 ± 2.7 ka, and a slightly younger result of 11.0 ± 1.0 ka for the more symmetrical sample OLOR16/SKG-2p1 (Fig. S12). The Bayesian-modeled ages are the preferred results for these dating experiments. With the above weighted-mean approach, there is a significant difference between the two samples at the 95% confidence level, whereas with the Bayesian approach the age difference between the samples does not meet this confidence level criterion.

## S3. Supporting Discussion

### S3.1. Present and past rainfall in Kenya

The topography of East Africa strongly modulates the amount and distribution of rainfall (Fig. S1*C*–S1*D*). Orographic precipitation is today prevalent across the central Kenya highlands (45), which receive mean annual rainfall of more than 2000 mm and have a positive moisture balance, in marked contrast to the adjacent semi-arid lowlands and the moisture-deficient rift valley (46) (Fig. S1*D*). The high rainfall at the 3000 and 4000 m high rift shoulders of the Mau Escarpment and the Aberdare Range generates runoff that today feeds into Lakes Naivasha, Nakuru, and Natron (47). During the early Holocene a stronger monsoonal circulation produced about 20-30% more rainfall in East Africa than today in response to higher Northern Hemisphere summer insolation and increased greenhouse gas forcing (48, 49, 50). Orographic forcing by the central Kenyan highlands would therefore have generated even more precipitation and runoff over mountainous areas at this time. Moisture supply to the Aberdare Range was indeed higher during the early Holocene than at present (51). The abrupt increase in rainfall with the onset of the Holocene (52, 53) would have

rapidly filled the headwater lake basins to their overflow levels, possibly within as little as 200 years according to lake-balance modeling (54). Enhanced dry season rainfall during the early Holocene (53) would have maintained river flow, in contrast to the present situation where flow in many Kenyan rivers is reduced or ceases during the biannual dry seasons (55, 56). Earlier cessation of overflow from Lake Nakuru-Elmenteita (by about 8.6 ka) than that of Lake Naivasha can be explained by the small catchment area of this lake basin as compared to the latter (46) (Fig. S1*C*).

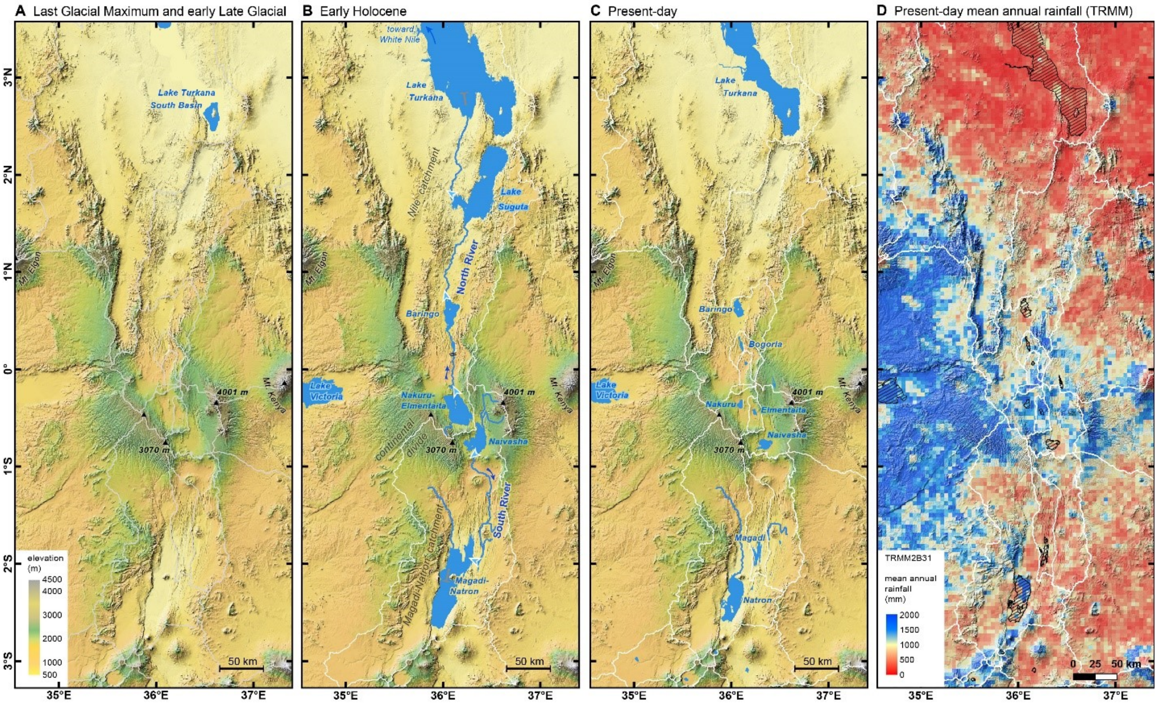


**Fig. S1.** Late Quaternary changes in river and lake extent and modern rainfall pattern**s** along the Kenya Rift. A) lake occurrence and extent during the Last Glacial Maximum and early Late Glacial (24-16 ka), b) occurrence and extent of lakes and rivers during the early Holocene (12-8 ka), C) present-day occurrence and extent of lakes and rivers, and D) estimates of mean annual rainfall (mm/yr) for 1998-2010 from the Tropical Rainfall Measuring Mission (57) satellite **o**peration at a resolution of 5x5 km (based on 2B31 TRMM product; annual mean compiled by B. Bookhagen).

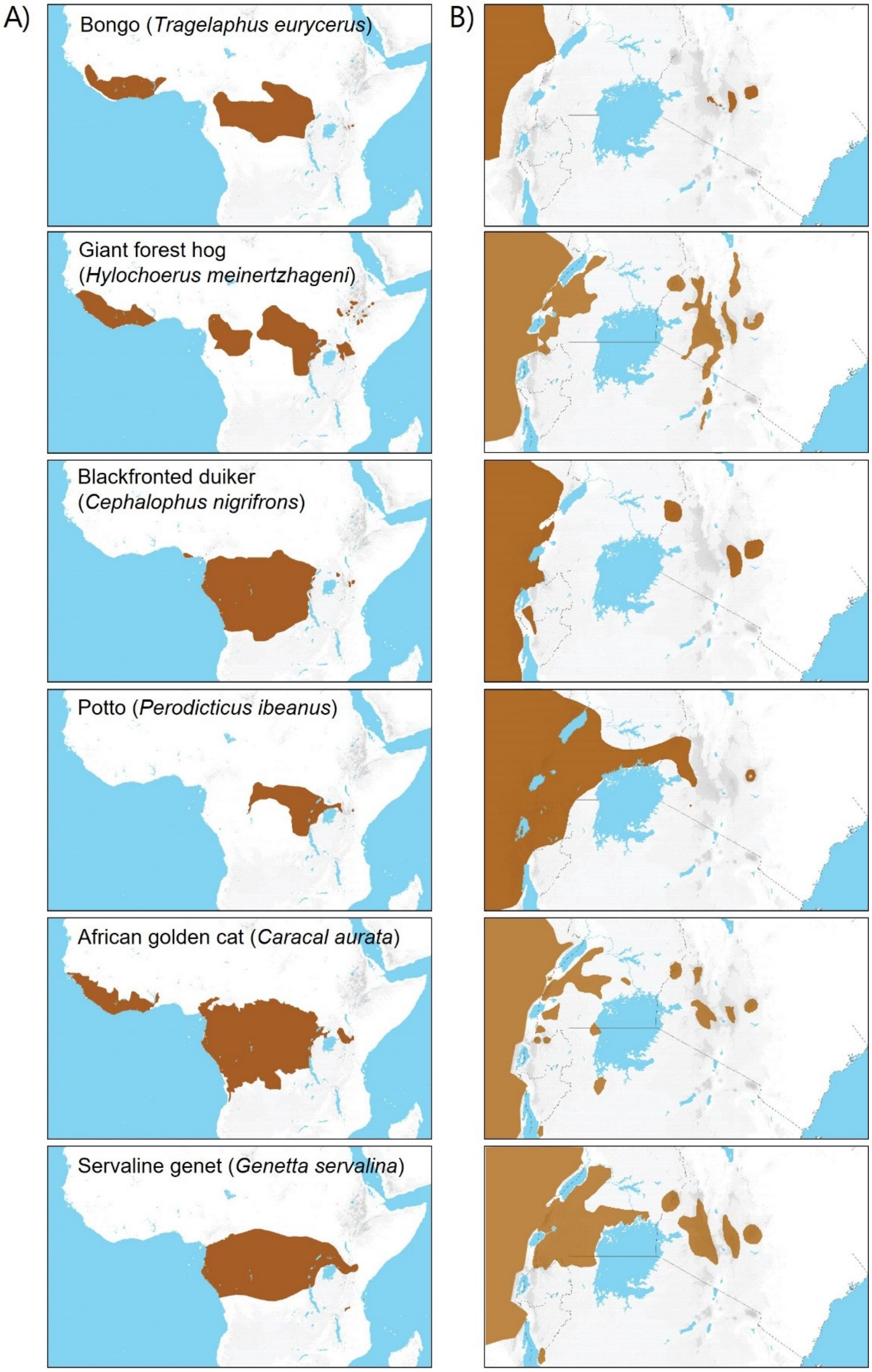


**Fig. S2.** Range maps of forest-dependent mammals with Guineo-Congolian distribution. A) Global distribution (Africa). B) detailed distribution of the same species in East Africa. Maps in A) based on ref. (58) and in B) on ref. (58, 59, 60, 61, 62).

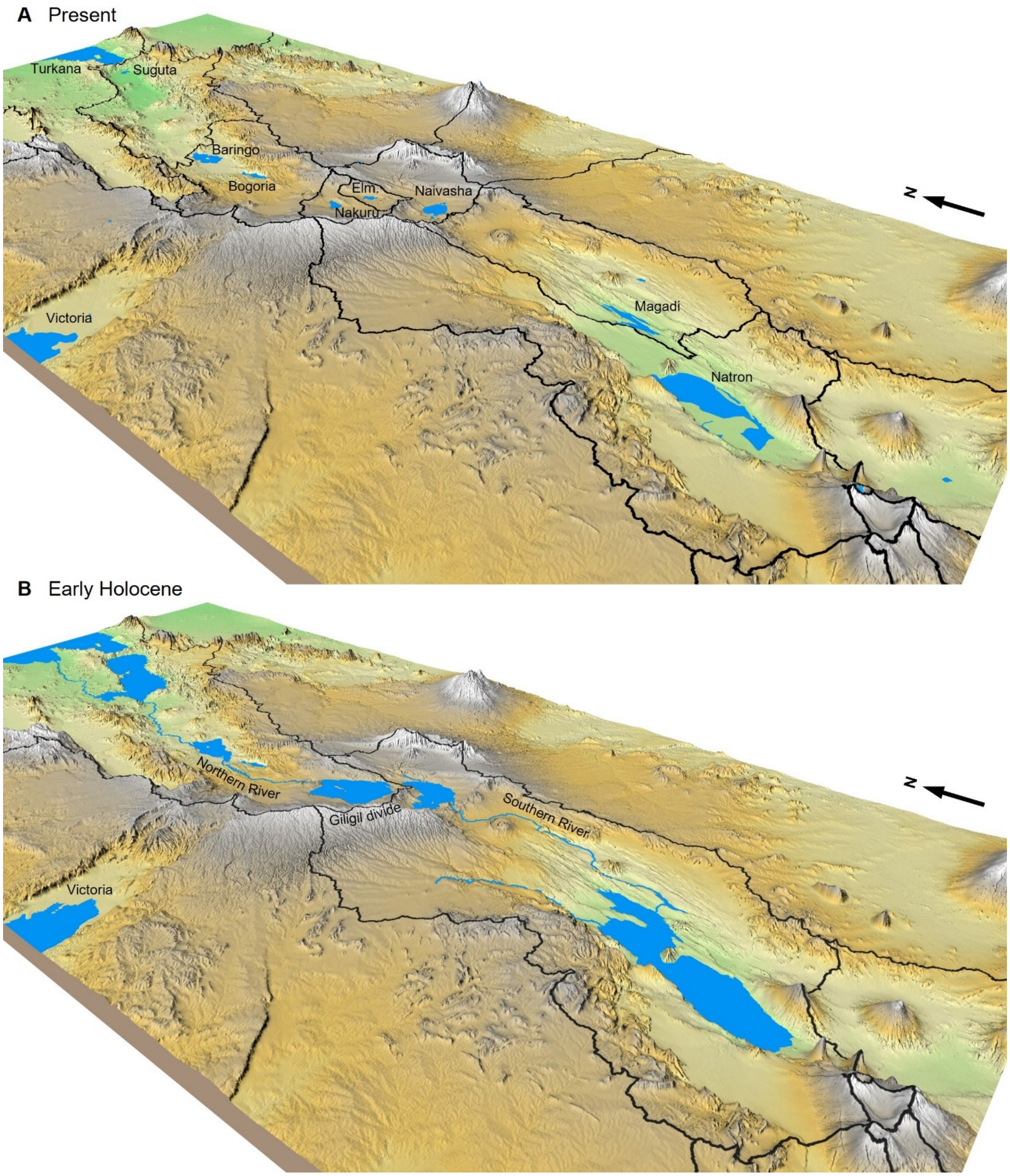


**Fig. S3.** Oblique views onto the Kenya Rift valley with its lakes, river systems and catchments. A) present-day setting. B) early Holocene setting. Catchment boundaries are outlined in black. Note the Gilgil divide as the highest area in the inner rift.

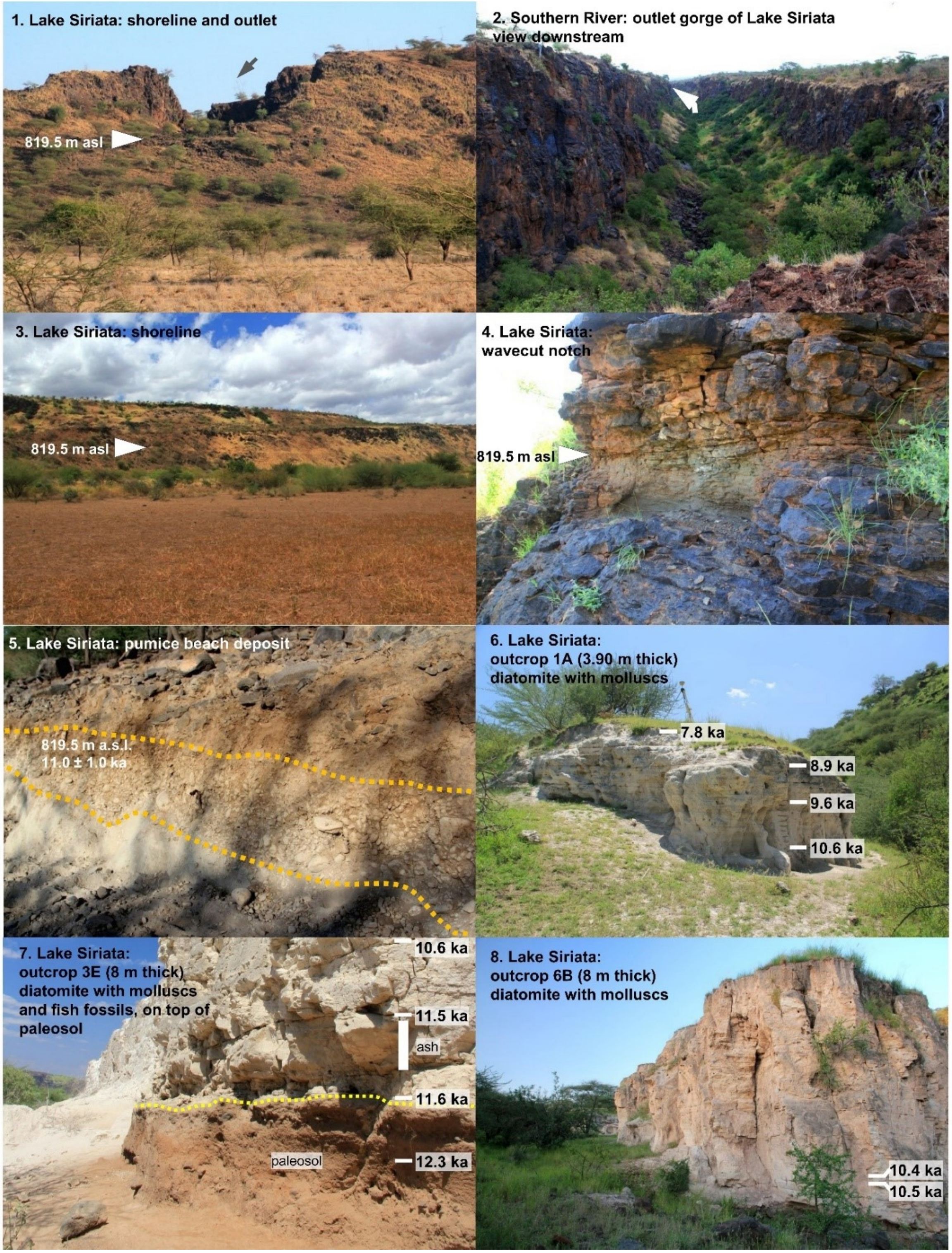
1. Lake Siriata: shoreline and outlet
819.5 m asl
2. Southern River: outlet gorge of Lake Siriata view downstream
3. Lake Siriata: shoreline
819.5 m asl
4. Lake Siriata: wavecut notch
819.5 m asl
5. Lake Siriata: pumice beach deposit
819.5 m a.s.l.
11.0 ± 1.0 ka
6. Lake Siriata: outcrop 1A (3.90 m thick) diatomite with molluscs
7.8 ka
8.9 ka
9.6 ka
10.6 ka
7. Lake Siriata: outcrop 3E (8 m thick) diatomite with molluscs and fish fossils, on top of paleosol
10.6 ka
11.5 ka
ash
11.6 ka
paleosol
12.3 ka
8. Lake Siriata: outcrop 6B (8 m thick) diatomite with molluscs
10.4 ka
10.5 ka

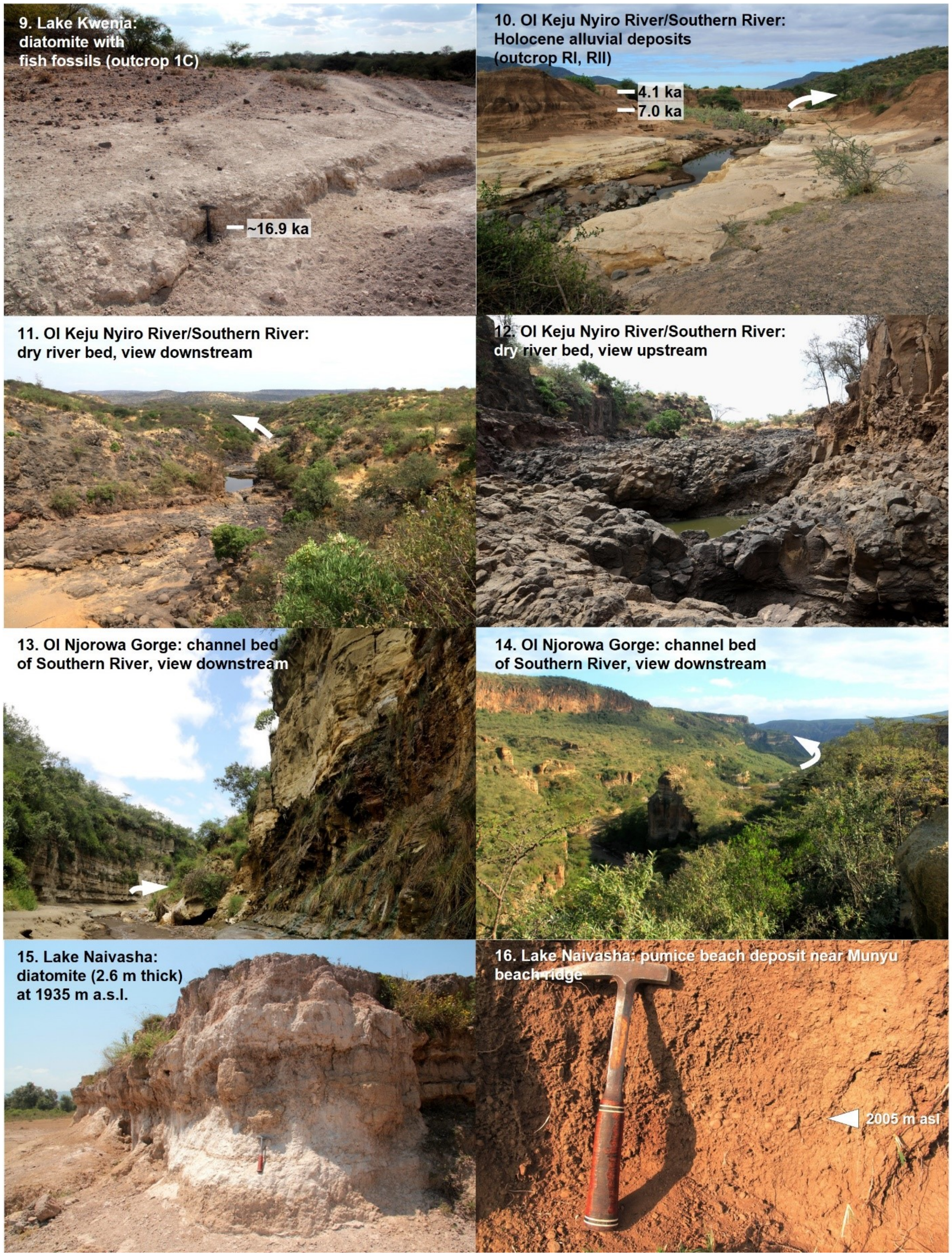
9. Lake Kwenia:
diatomite with
fish fossils (outcrop 1C)
~16.9 ka
10. Ol Keju Nyiro River/Southern River:
Holocene alluvial deposits
(outcrop RI, RII)
4.1 ka
7.0 ka
11. Ol Keju Nyiro River/Southern River:
dry river bed, view downstream
12. Ol Keju Nyiro River/Southern River:
dry river bed, view upstream
13. Ol Njorowa Gorge: channel bed
of Southern River, view downstream
14. Ol Njorowa Gorge: channel bed
of Southern River, view downstream
15. Lake Naivasha:
diatomite (2.6 m thick)
at 1935 m a.s.l.
16. Lake Naivasha: pumice beach deposit near Munyu
beach ridge
2005 m asl

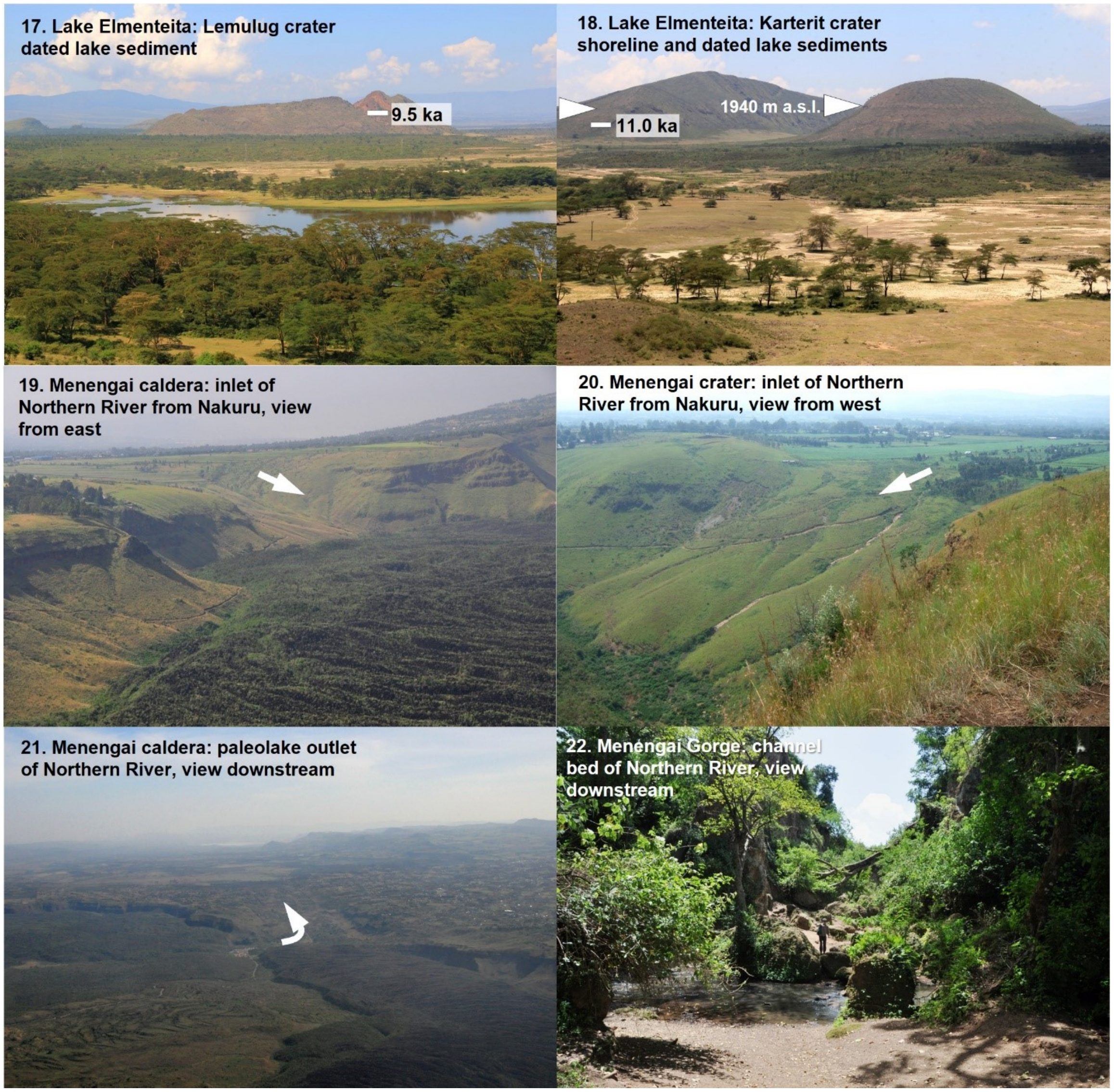


**Fig. S4.** Photos of study sites in the South and Central Kenya Rift showing vestiges of past river activity and lacustrine deposition at overflow lake levels. White arrows indicate river flow directions, white triangles shorelines and white bars dated unites (with median ages). Photos 1-18 by R. Dommain, photos 19-22 by S. Riedl.

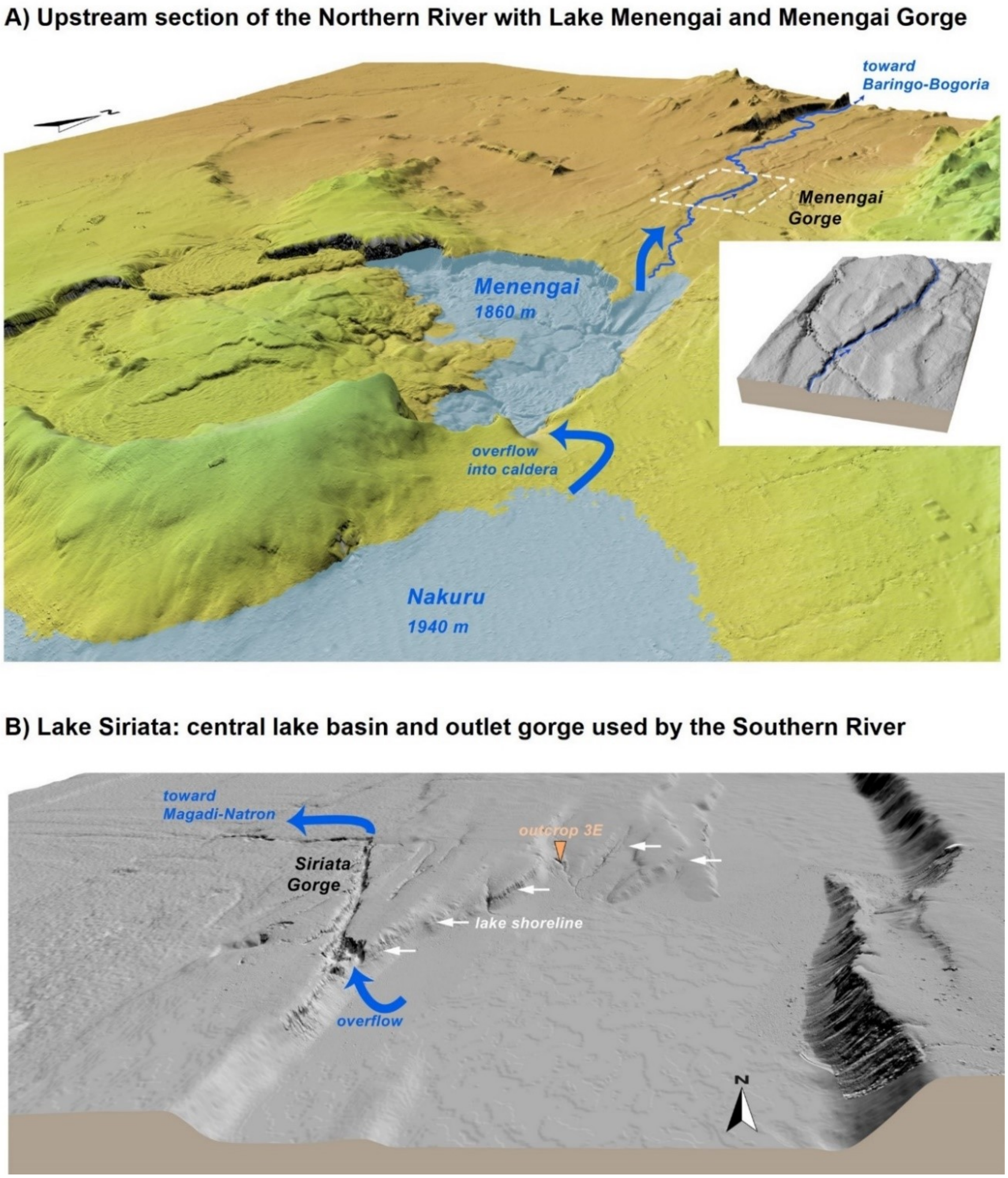


**Fig. S5.** Oblique views onto digital surface models (DSM) generated from Structure-from-Motion data for two key study sites with their early Holocene lake levels and active river systems. DSM in A) of the Menengai caldera lake region (DSM at 25 cm spatial resolution; see ref. 30), inset shows the Menengai Gorge, and in B) of the Lake Siriata outlet region (DSM at 20 cm spatial resolution merged with the ALOS World DEM); blue arrows indicate overflow directions through outlets and white arrows the Siriata highstand shoreline.

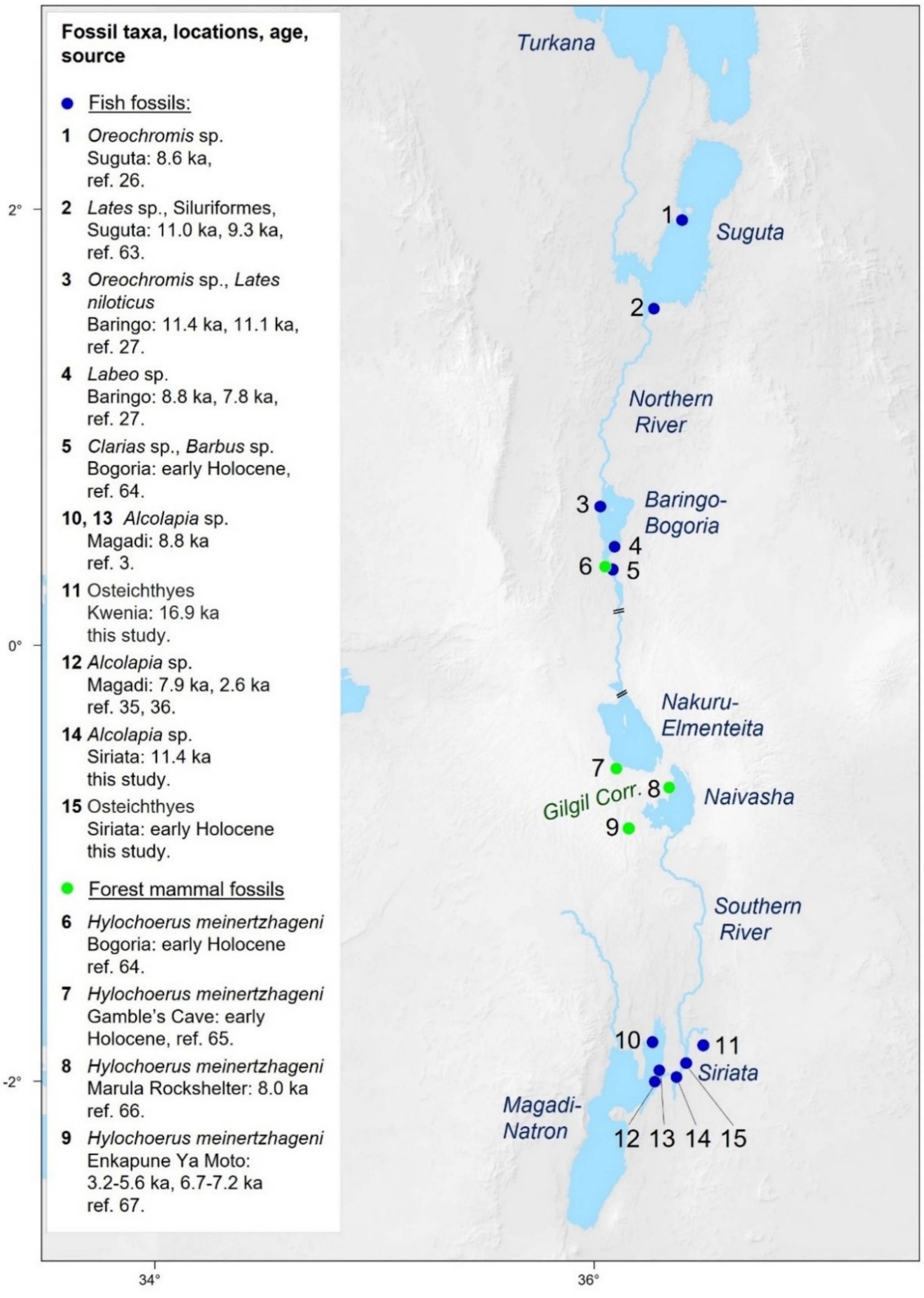


**Fig. S6.** Holocene fossil localities of freshwater fishes and forest mammals in the Kenya Rift valley. Blue dots mark sites with fish fossils and green dots sites with mammal fossils. Lake extent and river system of the early Holocene shown for context. Legend lists taxa, locations, dates, and references for the sites.

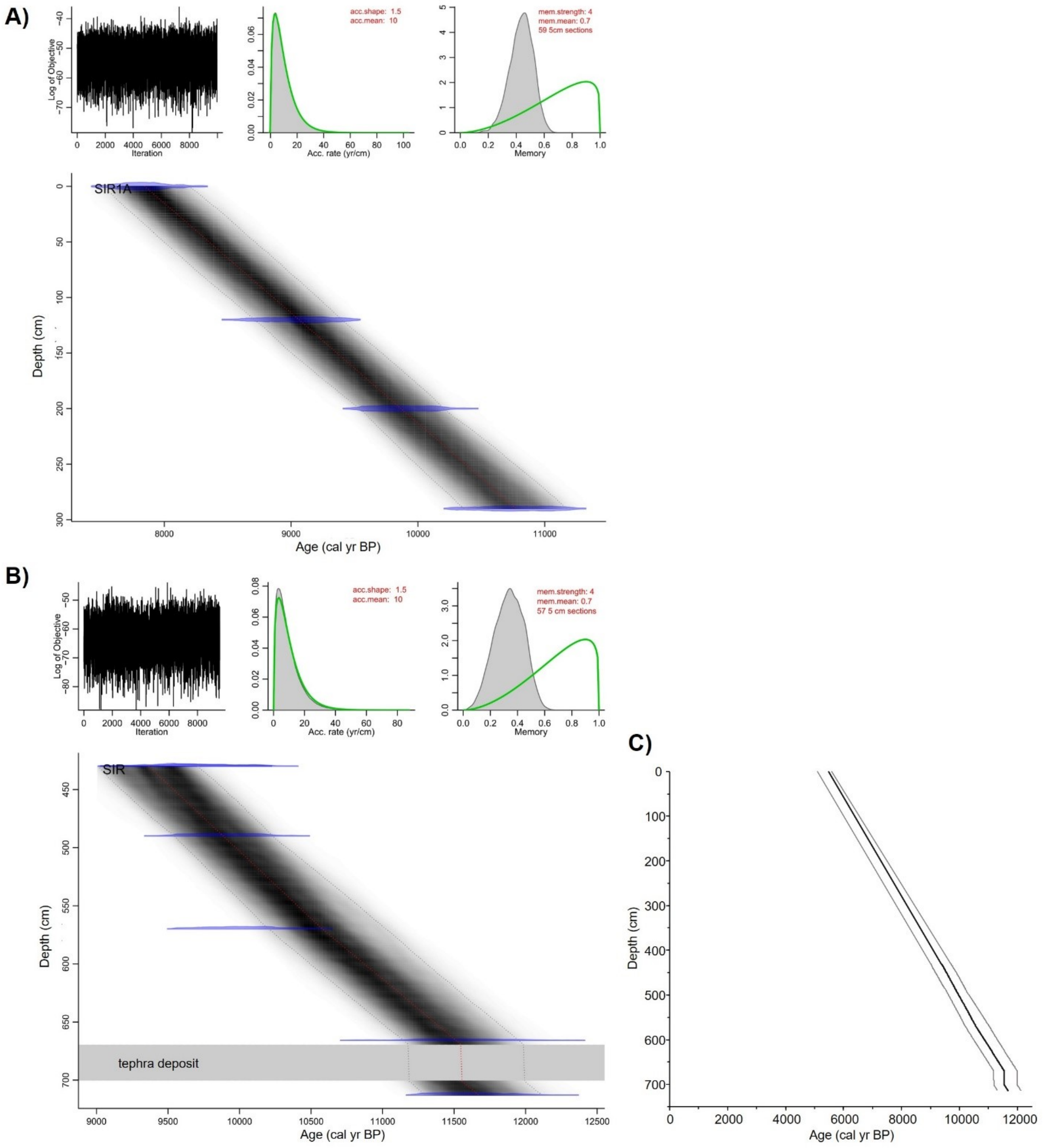


**Fig. S7.** Bayesian age models for Lake Siriata sediment outcrops generated with the rbacon package 2.3.8. A) age model for 290-0 cm of outcrop 1A. B) age model for the sediment package from 713-430 cm below surface of outcrop 3E, including an instantaneous deposit (tephra: 700-670 cm). C) age model for outcrop 3E extrapolated from 430 cm to 0 cm with same sedimentation rate as in between the uppermost two radiocarbon dates of B) and the same age uncertainties as in B), with the black line denoting the median age.

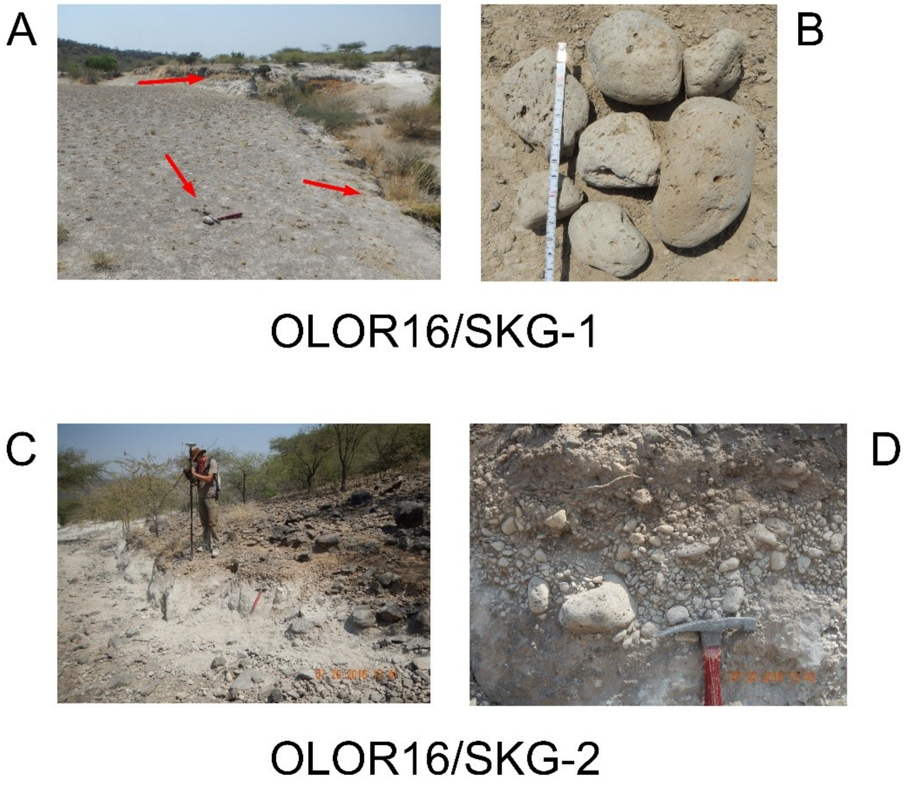


**Fig. S8.** Sample locations of pumice clasts in the Siriata basin. Sample OLOR16/SKG-1 (shown in A and B) is from the upper levels of diatomite beds at 1.961118°S, 36.367585°E (scale in B in cm). Sample OLOR16/SKG-2 is from a beach gravel at 1.963817°S, 36.366806°E.

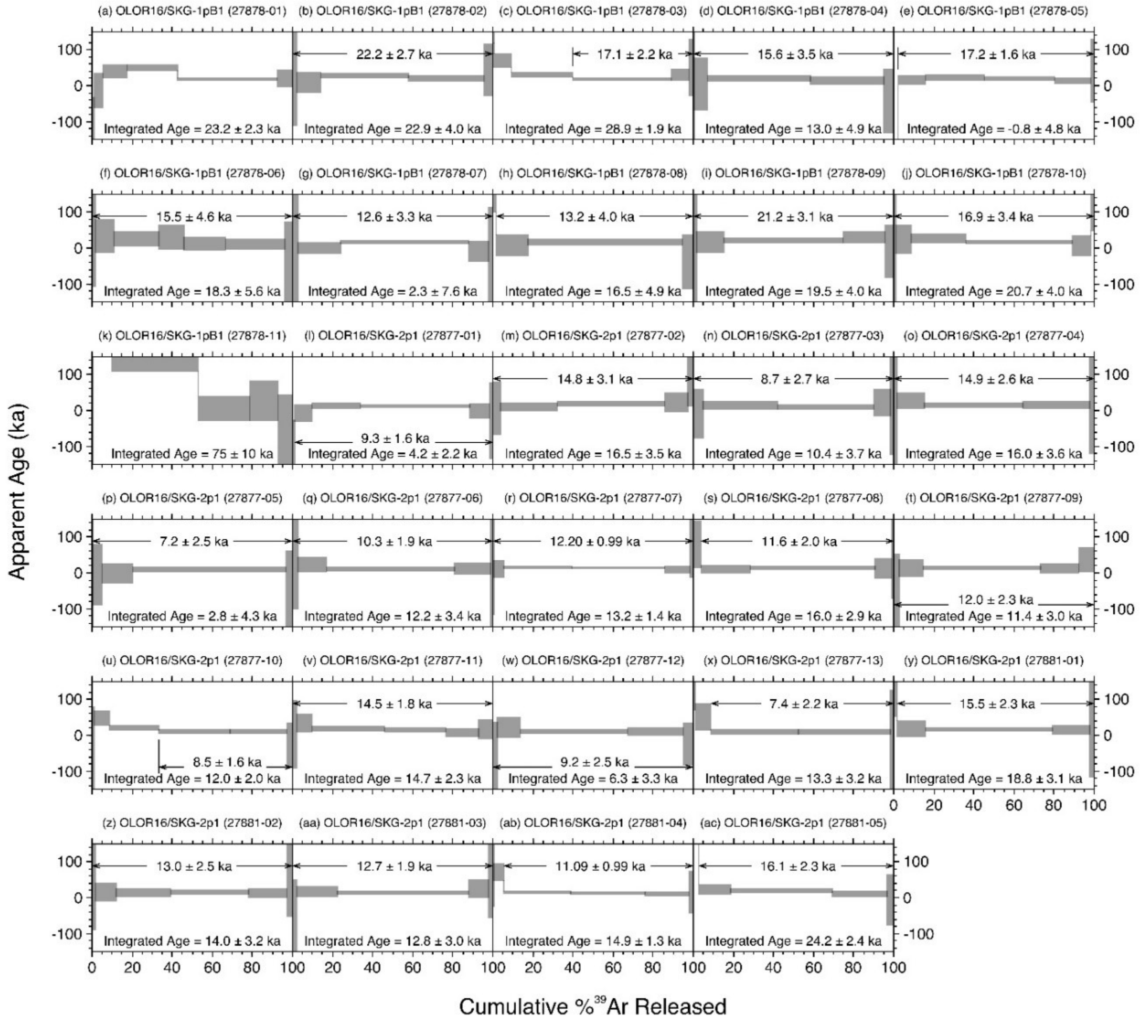


**Fig. S9.** Sanidine SCIH $^{39}Ar$ release spectra. 'Integrated Age' at the bottom of each panel page represents a computational re-combination of all gas steps. Apparent-age plateaus are shown by the double-barbed arrows. All uncertainties are 1s analytical error, and do not include the error in *J*, the neutron-fluence parameter in the $^{40}Ar/^{39}Ar$ age equation.

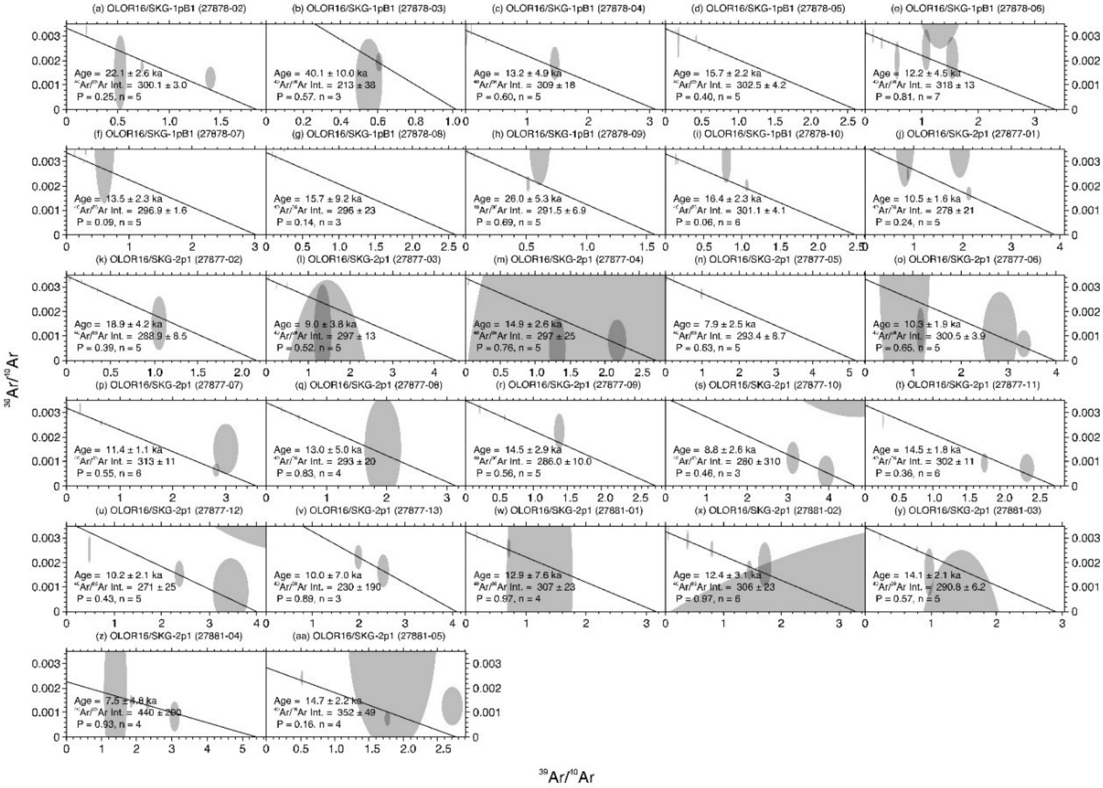


**Fig. S10.** 'Inverse' isochron plots ($^{36}Ar/^{40}Ar$ vs. $^{39}Ar/^{40}Ar$ isotope correlation diagrams) of the SCIH experiments. The isochron age is the x-axis intercept and is shown at 1s. '$^{40}Ar/^{39}Ar$ Int.' refers to 'trapped' non-radiogenic $^{40}Ar/^{36}Ar$ ratio derived from y-axis intercept of the isochron. 'MSWD' refers to 'mean square of weighted deviates,' a measure of the observed scatter about the fit line, compared to the expected scatter. 'P' refers to the probability that the observed scatter can be explained by analytical errors alone. 'n' is the number of analyses.

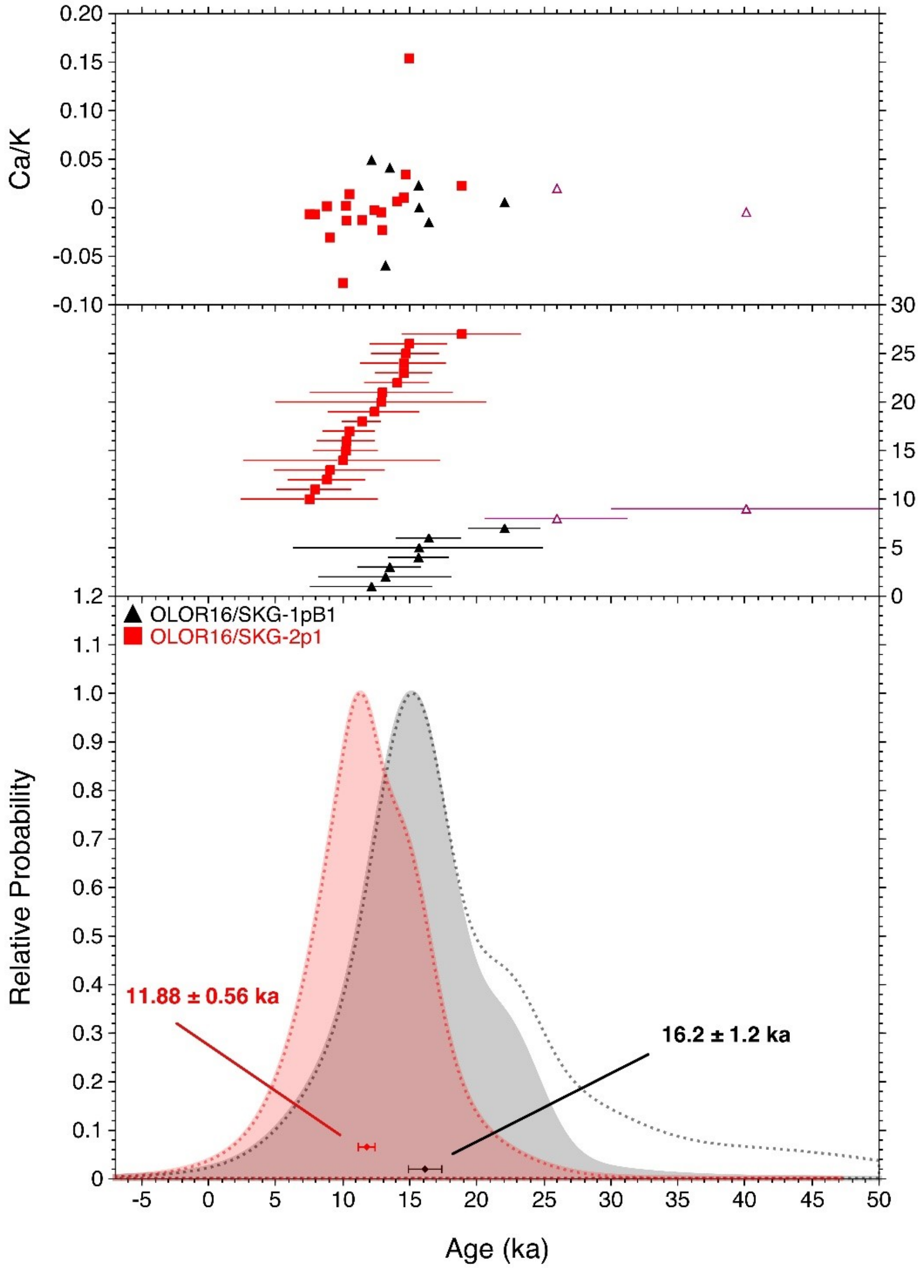


**Fig. S11.** Age-probability spectra of isochron ages, with weighted means. Sample legend is provided in lower-left. Top) Mean Ca/K atomic ratio of each aliquot derived as a by-product of the $^{40}Ar/^{39}Ar$ analysis. Middle) Rank order plot of the individual aliquots grouped by sample. Uncertainties in age are given at 1s standard error. Open symbols represent isochron ages omitted from the sample population based on the median outlier-detection criterion described in Methods. Bottom) Age-probability density spectra of each sample, with weighted-mean age and 1s error (including error in *J*, the neutron fluence parameter in $^{40}Ar/^{39}Ar$ dating). Dashed curves depict the probability density of the entire age population set, while the solid line represents the population after outlier deletion.

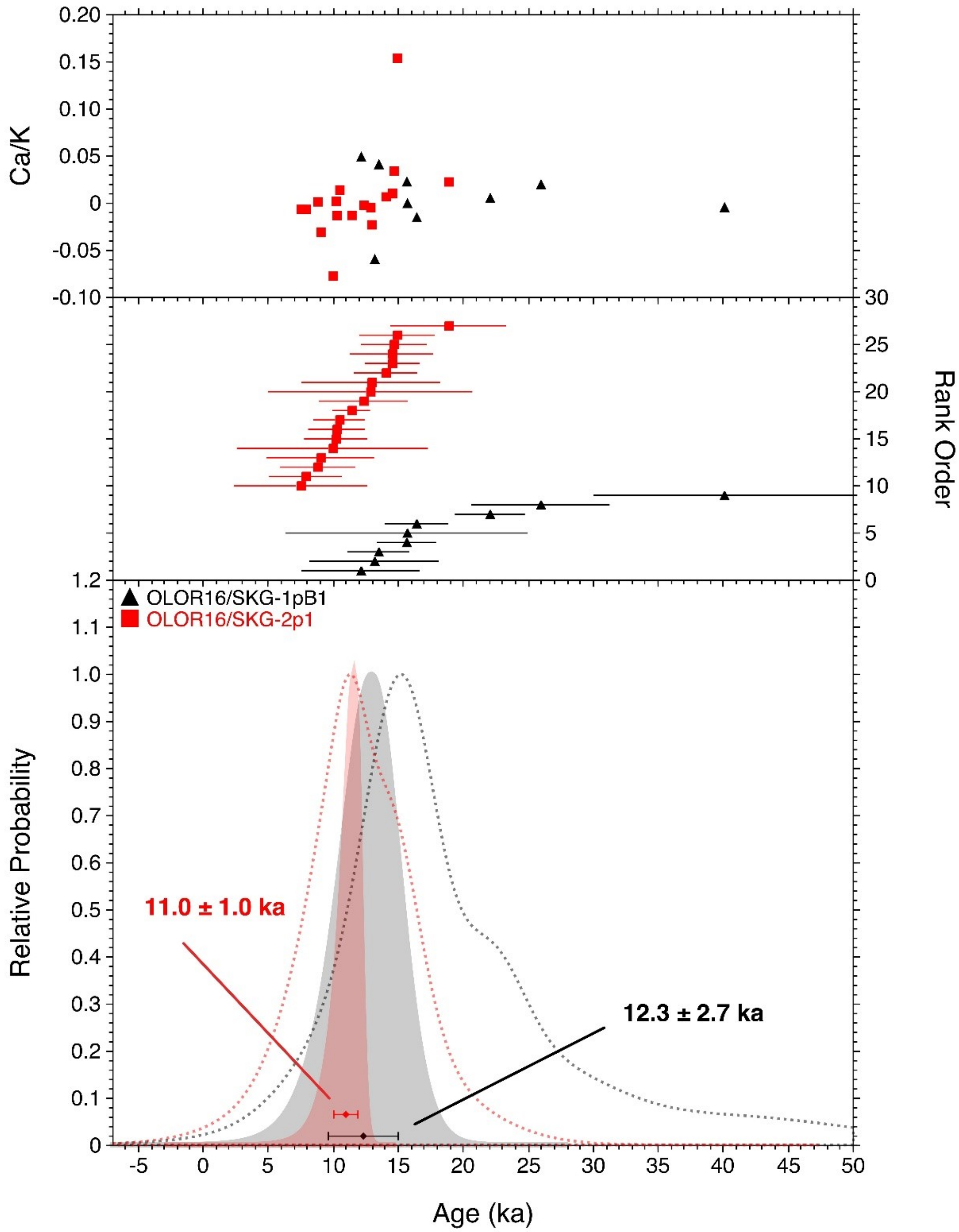


**Fig. S12.** Age-probability spectra of sanidine plateau ages, with Bayesian model distributions. The Bayesian model distributions are shown in solid lines with appropriate fill color (bottom). Remainder as in Fig. S10.

A)

B)

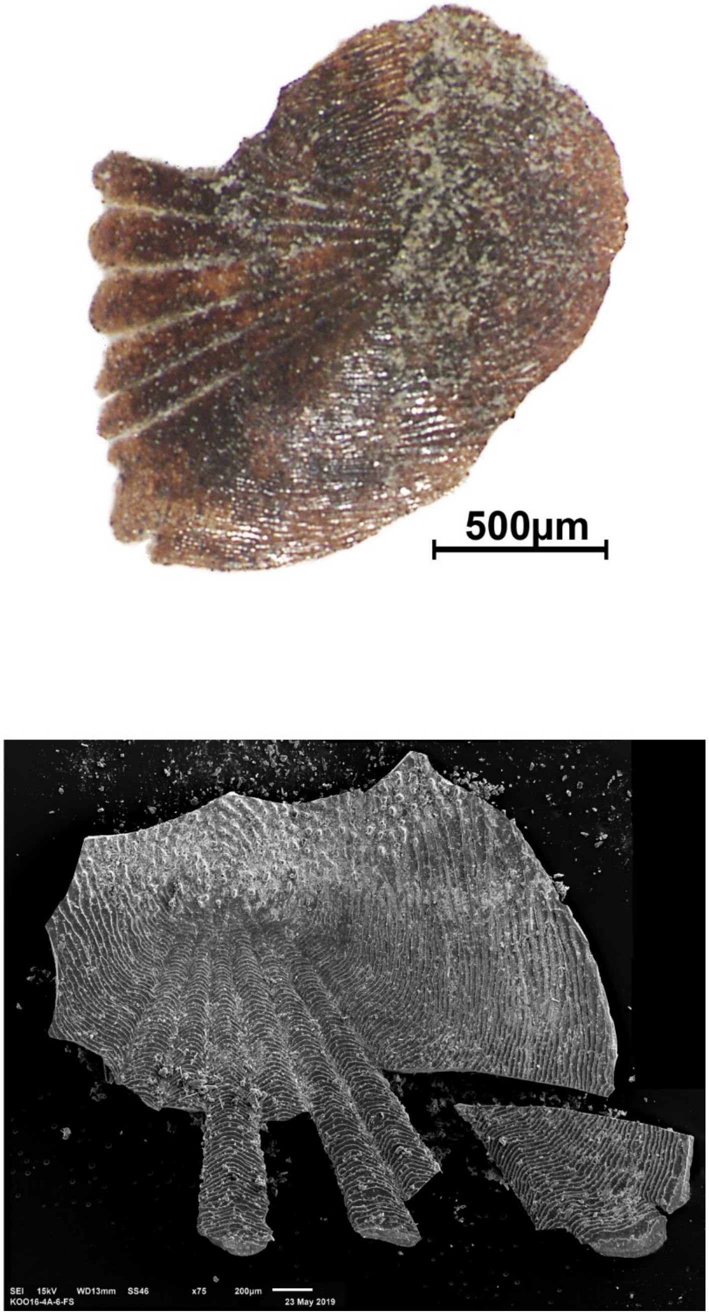


**Fig. S13.** Fish scale of *Oreochromis* cf. *Alcolapia* sp. from Lake Siriata early Holocene sediments. A) Color photo, B) SEM photo of the same scale with broken edges. The scale was retrieved from a bulk sediment sample from 650-670 cm below the top of outcrop 3E and dates to between 11.0 and 12.0 ka with a median age of 11.4 ka according to the BACON age model in Fig. S7.B.

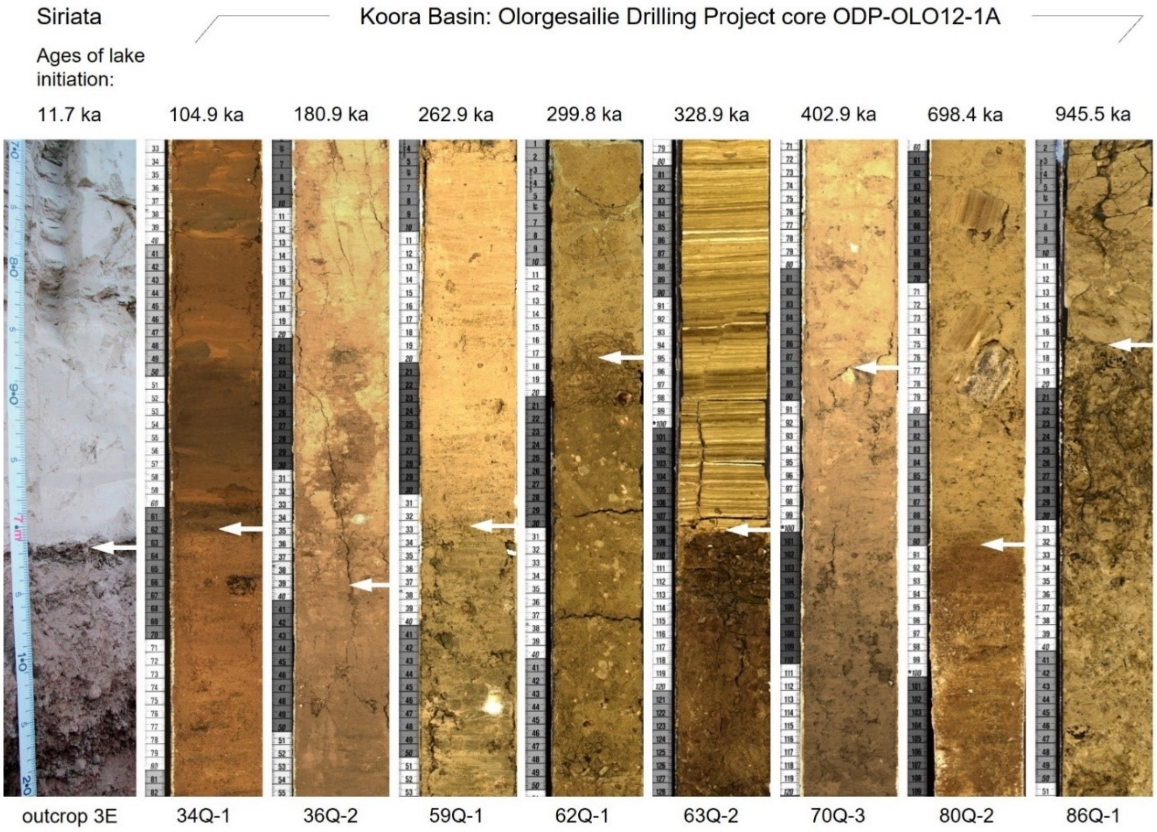


**Fig. S14.** Comparison of transitions from paleosols to lake sediments between Siriata outcrop 3E (left side) and the Koora Basin drill core OLO12-1A record. White arrows show approximate locations of lithological change from paleosol to overlying lake sediment, which indicate shifts from dry land to lake conditions, possible related to flooding from upstream sources. Ages at the top refer to these transitions (ages from ref. 37). Numbers at the bottom are core section IDs.

**Table S1.** List of fish species present in East African rift lakes.

| Species | Family | Distri-bution[1] | Natron | Magadi | Naivasha | Nak. | Elm. | Bogoria | Baringo | Suguta | Turka-na | Albert | Edward [2] | George [2] | References |
|---|---|---|---|---|---|---|---|---|---|---|---|---|---|---|---|
| *Alestes baremoze* | Alestidae | NS | | | | | | | | | 1 | 1 | | | 68, 69 |
| *Alestes dentex* | Alestidae | NS | | | | | | | | | 1 | 1 | “ | | 68, 69 |
| *Brycinus ferox* | Alestidae | e | | | | | | | | | 1 | | | | 68 |
| *Brycinus macrolepidotus* | Alestidae | NS, CB | | | | | | | | | 1 | 1 | | | 69, 70 |
| *Brycinus minutus* | Alestidae | e | | | | | | | | | 1 | | | | 68 |
| *Brycinus nurse* | Alestidae | NS | | | | | | | | | | 1 | | | 69 |
| *Brycinus nurse nana* | Alestidae | e / NS | | | | | | | | | 1 | | | | 68 |
| *Hydrocynus forskahlii* | Alestidae | NS | | | | | | | | | 1 | 1 | | | 68, 69 |
| *Hydrocynus vittatus* | Alestidae | NS, CB, Z | | | | | | | | | 1 | 1 | | | 68, 69 |
| *Micralestes* aff. *elongatus* | Alestidae | NS | | | | | | | | | 1 | | | | 68 |
| *Ctenopoma muriei* | Anabantidae | NS? | | | | | | | | | | 1 | 1 | 1 | 71, 72, 73 |
| *Bagrus bajad* | Bagridae | NS | | | | | | | | | 1 | 1 | | | 68, 69 |
| *Bagrus docmak* | Bagridae | NS | | | | | | | | | 1 | 1 | 1 | 1 | 68, 69, 71 |
| *Alcolapia alcalicus* | Cichlidae | e | 1 | | | | | | | | | | | | 68, 74 |
| *Alcolapia grahami* | Cichlidae | e | | 1 | | | | | | | | | | | 68, 74 |
| *Alcolapia latilabris* | Cichlidae | e | 1 | | | | | | | | | | | | 74 |
| *Alcolapia ndalalani* | Cichlidae | e | 1 | | | | | | | | | | | | 74 |
| *Astatoreochromis alluaudi* | Cichlidae | LVB | | | | | | | | | | | 1 | 1 | 75 |
| *Haplochromis aeneocolor* | Cichlidae | e | | | | | | | | | | | | 1 | 75 |
| *Haplochromis akika* | Cichlidae | e | | | | | | | | | | | | 1 | 72, 76 |
| *Haplochromis* (*Thoracochromis*) *albertianus* | Cichlidae | e | | | | | | | | | | 1 | | | 69 |
| *Haplochromis angustifrons* | Cichlidae | e | | | | | | | | | | | 1 | 1 | 71, 75 |
| *Haplochromis* (*Thoracochromis*) *avium* | Cichlidae | e | | | | | | | | | | 1 | | | 69 |
| *Haplochromis* (*Thoracochromis*) *bullatus* | Cichlidae | e | | | | | | | | | | 1 | | | 69 |
| *Haplochromis concilians* | Cichlidae | e | | | | | | | | | | | 1 | | 77 |
| *Haplochromis eduardianus* | Cichlidae | e | | | | | | | | | | | 1 | 1 | 75 |
| *Haplochromis eduardii* | Cichlidae | e | | | | | | | | | | | 1 | | 75, 78 |
| *Haplochromis elegans* | Cichlidae | e | | | | | | | | | | | 1 | 1 | 75 |
| *Haplochromis engystoma* | Cichlidae | e | | | | | | | | | | | 1 | | 75 |
| *Haplochromis erutus* | Cichlidae | e | | | | | | | | | | | 1 | | 77 |
| *Haplochromis fuscus* | Cichlidae | e | | | | | | | | | | | 1 | | 72, 79 |
| *Haplochromis gracilifur* | Cichlidae | e | | | | | | | | | | | 1 | | 80 |
| *Haplochromis labiatus* | Cichlidae | e | | | | | | | | | | | 1 | 1 | 75 |
| *Haplochromis limax* | Cichlidae | e | | | | | | | | | | | 1 | 1 | 75 |
| *Haplochromis* (*Thoracochromis*) *loati* | Cichlidae | N, LA | | | | | | | | | | 1 | | | 69 |
| *Haplochromis lobatus* | Cichlidae | e | | | | | | | | | | | 1 | | 81 |
| *Haplochromis* (*Thoracochromis*) *macconneli* | Cichlidae | e | | | | | | | | | 1 | | | | 68 |
| *Haplochromis macropsoides* | Cichlidae | e | | | | | | | | | | | 1 | 1 | 71, 75 |
| *Haplochromis* (*Thoracochromis*) *mahagiensis* | Cichlidae | e | | | | | | | | | | 1 | | | 69 |
| *Haplochromis mentatus* | Cichlidae | e | | | | | | | | | | | 1 | | 72 |
| *Haplochromis molossus* | Cichlidae | e | | | | | | | | | | | 1 | 1 | 80 |
| *Haplochromis mylodon* | Cichlidae | e | | | | | | | | | | | 1 | 1 | 75 |
| *Haplochromis nigripinnis* | Cichlidae | e | | | | | | | | | | | 1 | 1 | 71, 75 |
| *Haplochromis nubilus* | Cichlidae | LVB | | | | | | | | | | | 1 | 1 | 71, 75 |
| *Haplochromis oregosoma* | Cichlidae | e | | | | | | | | | | | | 1 | 75 |
| *Haplochromis pappenheimi* | Cichlidae | e | | | | | | | | | | | 1 | 1 | 75 |
| *Haplochromis paradoxus* | Cichlidae | e | | | | | | | | | | | 1 | | 80 |
| *Haplochromis pharyngalis* (syn. *H. petronius*) | Cichlidae | e | | | | | | | | | | | 1 | 1 | 75, 79 |
| *Haplochromis planus* | Cichlidae | e | | | | | | | | | | | 1 | | 77 |
| *Haplochromis relictidens* | Cichlidae | e | | | | | | | | | | | 1 | | 80 |
| *Haplochromis* (*Thoracochromis*) *rudolfianus* | Cichlidae | e | | | | | | | | | 1 | | | | 68 |
| *Haplochromis schubotzi* | Cichlidae | e | | | | | | | | | | | 1 | 1 | 75 |
| *Haplochromis schubotziellus* | Cichlidae | e | | | | | | | | | | | 1 | 1 | 75 |
| *Haplochromis serridens* | Cichlidae | e | | | | | | | | | | | 1 | | 72 |
| *Haplochromis squamipinnis* | Cichlidae | e | | | | | | | | | | | 1 | 1 | 75 |
| *Haplochromis taurinus* | Cichlidae | e | | | | | | | | | | | 1 | 1 | 71, 80 |
| *Haplochromis* (*Thoracochromis*) *turkanae* | Cichlidae | e | | | | | | | | | 1 | | | | 68 |
| *Haplochromis vicarius* | Cichlidae | e | | | | | | | | | | | 1 | 1 | 72 |
| *Haplochromis* (*Thoracochromis*) | Cichlidae | AN, LA | | | | | | | | | | 1 | | | 75 |

| | | | | | | | | | | | | | | | |
|---|---|---|---|---|---|---|---|---|---|---|---|---|---|---|---|
| *wingatii* | | | | | | | | | | | | | | | |
| *Hemichromis exsul* | Cichlidae | e | | | | | | | | | 1 | | | | 68 |
| *Neochromis "nigricans"* | Cichlidae | ? | | | | | | | | | | 1 | | | 69 |
| *Neochromis "zebra"* | Cichlidae | ? | | | | | | | | | | 1 | | | 69 |
| *Oreochromis leucostictus* | Cichlidae | AR | | | | | | | | | | 1 | 1 | 1 | 69, 71 |
| *Oreochromis niloticus baringoensis* | Cichlidae | e | | | | | | | 1 | | | | | | 68 |
| *Oreochromis niloticus eduardianus* | Cichlidae | AR | | | | | | | | | | 1 | 1 | 1 | 69, 71 |
| *Oreochromis niloticus* ssp "Bogoria?" | Cichlidae | e | | | | | | 1 | | | | | | | 82, 83 |
| *Oreochromis niloticus sugutae* | Cichlidae | e | | | | | | | | 1 | | | | | 68 |
| *Oreochromis niloticus vulcani* | Cichlidae | e | | | | | | | | | 1 | | | | 68 |
| *Pseudocrenilabrus multicolor* | Cichlidae | LVB, N | | | | | | | | | | 1 | | 1 | 72, 75 |
| *Sarotherodon galilaeus* | Cichlidae | NS, CB | | | | | | | | | 1 | 1 | | | 68 |
| *Tilapia* (*Coptodon*) *zillii* | Cichlidae | NS, CB | | | | | | | | | 1 | 1 | | | 68, 71 |
| *Citharinus citharus* | Citharinidae | NS | | | | | | | | | 1 | 1 | | 1 | 68, 69, 73 |
| *Citharinus latus* | Citharinidae | NS | | | | | | | | | | 1 | | | 69 |
| *Clarias alluaudi* | Clariidae | LVB, AR | | | | | | | | | | 1 | 1 | | 69, 72 |
| *Clarias gariepinus* | Clariidae | pan-Africa | | | | | | 1 | 1 | 1 | 1 | 1 | 1 | 1 | 68, 69, 71 |
| *Clarias hilli* | Clariidae | CB | | | | | | | | | | 1 | | | 84 |
| *Clarias liocephalus* | Clariidae | LVB, AR, CB | | | | | | | | | | 1 | 1 | 1 | 69, 72 |
| *Heterobranchus longifilis* | Clariidae | NS, CB, Z | | | | | | | | | 1 | | | | 68 |
| *Auchenoglanis biscutatus* | Claroteidae | NS | | | | | | | | | 1 | | | | 85 |
| *Auchenoglanis occidentalis* | Claroteidae | NS, CB | | | | | | | | | 1 | 1 | | | 68, 69 |
| *Chrysichthys turkana* | Claroteidae | e | | | | | | | | | 1 | | | | 86 |
| *Barbus* spec. "Baringo" | Cyprinidae | e | | | | | | 1 | 1 | | | | | | 68 |
| *Barbus turkanae* | Cyprinidae | e | | | | | | | | | 1 | | | | 68 |
| *Barilius niloticus* (syn. *Leptocypris niloticus*) | Cyprinidae | NS | | | | | | | | | | 1 | | | 69 |
| *Chelaethiops bibie* | Cyprinidae | NS | | | | | | | | | 1 | | | | 68 |
| *Enteromius* (*Barbus*) *neglectus* | Cyprinidae | N | | | | | | | | | | 1 | | | 69 |
| *Enteromius* (*Barbus*) *neumayeri* | Cyprinidae | LVB, LT | | | | | | 1 | | | 1 | 1 | | | 68, 87 |
| *Enteromius* (*Barbus*) *perince* | Cyprinidae | NS | | | | | | | | | | 1 | | | 69 |
| *Enteromius* (*Barbus*) *stigmatopygus* | Cyprinidae | NS | | | | | | | | | 1 | | | | 68 |
| *Enteromius* cf. *mimus* | Cyprinidae | EC | | | | | | | | | | | 1 | 1 | 88 |
| *Enteromius kerstenii* | Cyprinidae | Z, LVB, EC | | | | | | | | | | | 1 | 1 | 71 |
| *Enteromius alberti* | Cyprinidae | LVB | | | | | | | | | | | 1 | 1 | 88 |
| *Enteromius apleurogram ma* | Cyprinidae | LVB | | | | | | | | | | | 1 | 1 | 89 |
| *Labeo coubie* | Cyprinidae | NS | | | | | | | | | 1 | 1 | | | 69, 85 |
| *Labeo cylindricus* | Cyprinidae | Z, EC, CB | | | | | | 1 | 1 | 1 | 1 | | | | 68 |
| *Labeo forskalii* | Cyprinidae | N | | | | | | | | | | | 1 | | 72 |
| *Labeo horie* | Cyprinidae | N | | | | | | | | | 1 | 1 | | | 68, 69 |
| *Labeo niloticus* | Cyprinidae | NS | | | | | | | | | 1 | | | | 68 |
| *Labeobarbus altianalis* | Cyprinidae | LVB, AR | | | | | | | | | | | 1 | 1 | 71 |
| *Labeobarbus bynni* | Cyprinidae | NS | | | | | | | | | 1 | 1 | | | 68, 69 |
| *Labeobarbus intermedius* | Cyprinidae | NS | | | | | | 1 | 1 | 1 | 1 | | | | 68 |
| *Leptocypris niloticus* | Cyprinidae | N | | | | | | | | | 1 | | | | 68 |
| *Neobola* (*Engraulicypris*) *bredoi* | Cyprinidae | e | | | | | | | | | | 1 | | | 69 |
| *Neobola stellae* | Cyprinidae | e | | | | | | | | | 1 | | | | 68 |
| *Raiamas senegalensis* | Cyprinidae | NS | | | | | | | | | 1 | | | | 68 |
| *Distichodus nefasch* (syn. *D. niloticus*) | Distichodontida e | N | | | | | | | | | 1 | 1 | | | 68, 69 |
| *Distichodus rostratus* | Distichodontida e | NS | | | | | | | | | | 1 | | | 69 |
| *Gymnarchus niloticus* | Gymnarchidae | NS | | | | | | | | | 1 | | | | 68 |
| *Lates longispinis* | Latidae | e | | | | | | | | | 1 | | | | 68 |
| *Lates macrophthalmus* | Latidae | e | | | | | | | | | | 1 | | | 69 |
| *Lates niloticus* | Latidae | NS | | | | | | | | | 1 | 1 | | | 68, 69 |
| *Malapterurus electricus* | Malapteruridae | NS | | | | | | | | | 1 | 1 | | | 68, 69 |
| *Mochokus niloticus* | Mochokidae | NS | | | | | | | | | 1 | | | | 68 |
| *Synodontis filamentosus* | Mochokidae | NS | | | | | | | | | 1 | | | | 85 |
| *Synodontis frontosus* | Mochokidae | N | | | | | | | | | 1 | 1 | | | 68, 69 |
| *Synodontis nigrita* | Mochokidae | NS | | | | | | | | | | 1 | | | 69 |
| *Synodontis schall* | Mochokidae | NS | | | | | | | | | 1 | 1 | | | 68, 69 |
| *Gnathonemus cyprinoides* | Mormyridae | NS | | | | | | | | | | 1 | | | 69 |
| *Hyperopisus bebe* | Mormyridae | NS | | | | | | | | | 1 | 1 | | | 68, 69 |
| *Marcusenius petherici* | Mormyridae | N | | | | | | | | | | 1 | | | 69 |
| *Mormyrops anguilloides* | Mormyridae | NS, CB, Z | | | | | | | | | | 1 | | | 69 |
| *Mormyrus caschive* | Mormyridae | N | | | | | | | | | 1 | 1 | | | 69, 85 |
| *Mormyrus kannume* | Mormyridae | N, LVB | | | | | | | | | 1 | 1 | 1 | 1 | 71, 85 |
| *Mormyrus macrocephalus* | Mormyridae | VN, LK | | | | | | | | | | 1 | | | 69 |
| *Mormyrus niloticus* | Mormyridae | N | | | | | | | | | | 1 | | | 69 |
| *Pollimyrus* (*Marcusenius*) *nigricans* | Mormyridae | LVB, AR | | | | | | | | | | 1 | | 1 | 69, 73 |

| | | | | | | | | | | | | | | | |
|---|---|---|---|---|---|---|---|---|---|---|---|---|---|---|---|
| *Nothobranchius albertinensis* | Notobranchiidae | e | | | | | | | | | | 1 | | | 90 |
| *Nothobranchius ugandensis* | Notobranchiidae | LVB, LA | | | | | | | | | | 1 | | | 91 |
| *Heterotis niloticus* | Osteoglossidae | NS, CB | | | | | | | | | 1 | | | | 68 |
| *Polypterus bichir* | Polypteridae | NS | | | | | | | | | 1 | | | | 68 |
| *Polypterus senegalus* | Polypteridae | NS | | | | | | | | | 1 | 1 | | | 68, 69 |
| *Aplocheilichthys jeanneli* (syn. *Lacustricola jeanneli*) | Procatopodidae | e | | | | | | | | | 1 | | | | 68 |
| *Aplocheilichthys mahagieinsis* | Procatopodidae | ? | | | | | | | | | | 1 | | | 69 |
| *Aplocheilichthys rudolfianus* | Procatopodidae | e | | | | | | | | | 1 | | | | 68 |
| *Aplocheilichthys* spec. "Baringo" | Procatopodidae | possibly e | | | | | | 1 | | | | | | | 68 |
| *Aplocheilichthys* spec. "Naivasha" | Procatopodidae | e | | | 1 | | | | | | | | | | 68 |
| *Laciris pelagica* | Procatopodidae | e | | | | | | | | | | | 1 | | 71 |
| *Lacustricola bukobanus* | Procatopodidae | AR, LV | | | | | | | | | | | 1 | 1 | 71 |
| *Lacustricola kassenjiensis* | Procatopodidae | AR | | | | | | | | | | 1 | | | 69 |
| *Lacustricola vitschumbaensis* | Procatopodidae | AR, LV | | | | | | | | | | | 1 | 1 | 71 |
| *Protopterus aethiopicus* | Protopteridae | N, CB | | | | | | | | | 1 | 1 | 1 | 1 | 68, 69, 71 |
| *Schilbe intermedius* | Schilbeidae | NS, CB, Z, EC | | | | | | | | | | 1 | | | 69 |
| *Schilbe mystus* (syn. *Eutropius niloticus*) | Schilbeidae | NS | | | | | | | | | | 1 | | | 69 |
| *Schilbe uranoscopus* | Schilbeidae | NS | | | | | | | | | 1 | | | | 68 |
| *Tetraodon lineatus* | Tetraodontidae | NS | | | | | | | | | 1 | | | | 68 |
| | | **Sum species** | **3** | **1** | **1** | **0** | **0** | **6** | **6** | **4** | **57** | **62** | **48** | **39** | |
| | | **Endemic spp.** | 3 | 1 | 1 | 0 | 0 | 2 | 3 | 1 | 14 | 7 | 29 | 19 | |

[1] species distribution based on listed references and information retrieved from www.fishbase.se (ref. 72, accessed May 5 2021). Abbreviations: AN = Albert Nile, AR = Albertine Rift, CB = Congo Basin, e = endemic, EC = East Coast, LA = Lake Albert, LK = Lake Kyoga, LT = Lake Tanganyika, LV = Lake Victoria, LVB = Lake Victoria Basin, N = Nile, NS = Nilo-Sudan, VN = Victoria Nile, Z = Zambezi.
[2] total number of fish species for Lakes Edward and George remains unknown, because many endemic cichlid species have not been formally described (71).
Nak. = Nakuru, Elm. = Elmenteita

**Table S2.** Annotated list of published radiocarbon dates used to reconstruct lake overflow and closed lake conditions.

| # | Lab ID | $^{14}$C date (BP) | Reservoir corrected $^{14}$C date (BP) | Calibr. median age (cal. yr BP) | Dated material | Sample location | Pub-lished eleva-tion (m) | Additional published information | Interpretation | Geogr. coordi-nates (°)[2] | Reference |
|---|---|---|---|---|---|---|---|---|---|---|---|
| ***Turkana*** | | | | | | | | | | | |
| 1 | Gx-4642-I-A | 3405±130 | | 3667 | Bone apatite | Koobi Fora | 416 | Beach sand | Closed basin | 3.94°N?, 36.2°E? | 8, 92 |
| 2 | L-1203-H | 3650±150 | | 3987 | mixed shell | Kibish | 443 | Beach ridge | Closed basin | 5.3167°N, 35.926°E | 8, 93, 94 |
| 3 | SUA-637 | 3945±135 | | 4392 | Charcoal | Koobi Fora | 416 | Beach sand | Closed basin | 3.94°N?, 36.2°E? | 8, 92 |
| 4 | P-2610 | 3960±60 | | 4418 | Charcoal | Koobi Fora | 416 | Beach sand | Closed basin | 3.94°N?, 36.2°E? | 8, 92 |
| 5 | P-2609 | 3970±60 | | 4434 | Charcoal | Koobi Fora | 412 | Beach sand | Closed basin | 3.92°N?, 36.225°E? | 8, 92 |
| 6 | SUA-637-b | 4100±125 | | 4616 | Humic acid | Koobi Fora | 416 | Beach sand | Closed basin | 3.94°N?, 36.2°E? | 8, 92 |
| 7 | SUA-634 | 4160±110 | | 4679 | Charcoal | Koobi Fora | 412 | Beach sand | Closed basin | 3.92°N?, 36.225°E? | 8, 92 |
| 8 | KIA36859 | 4330±30 | | 4891 | *Melanoides tuberculata* | Lake Turkana South Island | 427 | Beach ridge | Closed basin | 2.6261°N, 36.5712°E | 8 |
| 9 | KIA36861 | 4370±35 | | 4931 | *Melanoides tuberculata* | Lake Turkana South Island | 410 | Beach ridge | Closed basin | 2.6247°N, 36.5715°E | 8 |
| 10 | Gx-5475-A | 4560±185 | | 5212 | Bone apatite | Koobi Fora | 417 | Beach sand | Closed basin | 3.95°N?, 36.25°E? | 8, 92 |
| 11 | Gx-4642-II-A | 4580±170 | | 5238 | Bone apatite | Koobi Fora | 416 | Beach sand | Closed basin | 3.94°N?, 36.2°E? | 8, 92 |
| 12 | KIA36858 | 4645±35 | | 5406 | *Melanoides tuberculata* | Lake Turkana South Island | 438 | Beach ridge | Closed basin | 2.6272°N, 36.572°E | 8 |
| 13 | KIA36860 | 4680±35 | | 5400 | *Melanoides tuberculata* | Lake Turkana South Island | 418 | Beach ridge | Closed basin | 2.6255°N, 36.5716°E | 8 |
| 14 | KIA 36865 | 4695±35 | | 5403 | *Melanoides tuberculata* | Lake Turkana South Island | 393 | Beach ridge | Closed basin | 2.6192°N, 36.5711°E | 8 |
| 15 | n.a. | 4800±100 | | 5520 | *Etheria* | Eliye Springs (Turkana) | 439 | Beach ridge | Closed basin | 3.12°N, 35.98°E | 8, 95, 96 |

| | | | | | | | | | | |
|---|---|---|---|---|---|---|---|---|---|---|
| 16 | Y-1575 | 4880±100 | 5624 | *Etheria elliptica* | Eliye Springs (Turkana) | 427 | Beach ridge | Closed basin | 3°N, 36°E | 8, 97 |
| 17 | KIA 36862 | 4910±35 | 5635 | Bivalve Shell | Lake Turkana South Island | 405 | Beach ridge | Closed basin | 2.6237°N, 36.5713°E | 8 |
| 18 | Birm-540 | 4940±230 | 5682 | *Etheria elliptica* | Koobi Fora | 429 | Beach ridge | Closed basin | 3.9667°N?, 36.2°E | 8, 63, 92 |
| 19 | L-1303-A | 5550±350 | 6353 | mixed shell | Kibish | 440 | Beach ridge | Closed basin | 5.1667°N, 35.5863°E | 8, 94 |
| 20 | N-1101 | 6010±160 | 6869 | Shell | Lothagam | 463 | Shell beds | Overflow | 2.8°N?, 36°E? | 8, 95 |
| 21 | N-812 | 6200±130 | 7089 | Charcoal | Lothagam | 430 | Beach sands | Closed basin | 2.8°N?, 36°E? | 8, 94, 98 |
| 22 | L-1303-D | 7000±150 | 7831 | mixed shell | Kibish | 366 | Transgressive sandstone | Closed basin | 4.4667°N, 36.9403°E | 8, 94 |
| 23 | Gx-5476-A | 7855±160 | 8713 | Bone apatite | Koobi Fora | 436 | Sand bar | Closed basin | 3.956°N, 36.375°E | 8, 92 |
| 24 | N-813 | 7960±140 | 8823 | Shell | Lothagam | 463 | Shell beds | Overflow | 2.839°N?, 35.9641°E? | 8, 95 |
| 25 | KIA 36856 | 8180±45 | 9123 | *Melanoides tuberculata* | Lake Turkana South Island | 454 | Abrasion platform, interpreted as end of overflow | Overflow | 2.6611°N, 36.5879°E | 8 |
| 26 | N-1102 | 8230±180 | 9180 | Shell | Lothagam | 456 | Dark organic sediment | Overflow | 2.8421°N?, 36.0206°E? | 8, 95 |
| 27 | Gx-5480-A | 8355±235 | 9306 | Bone apatite | Koobi Fora | 457 | Sand spits | Overflow | 4.077°N?, 36.347°E? | 8, 92 |
| 28 | Gx-5481-A | 8395±270 | 9356 | Bone apatite | Koobi Fora | 457 | Sand spits | Overflow | 4.077°N?, 36.347°E? | 8, 92 |
| 29 | N-1100 | 8420±170 | 9386 | Shell | Lothagam | 463 | Sands | Overflow | 2.8°N?, 36°E? | 8, 98 |
| 30 | Hel-1276 | 8920±130 | 9995 | Shell | Koobi Fora | 436 | Sand bar | Interruption of Overflow | 3.956°N?, 36.375°E? | 8, 92 |
| 31 | Hel-1277 | 9110±130 | 10290 | *Etheria* | Koobi Fora | 436 | Sand bar | Interruption of Overflow | 3.956°N?, 36.375°E? | 8, 92 |
| 32 | SUA-635 | 9315±140 | 10527 | Shell | Koobi Fora | 412 | Beach sand | Interruption of Overflow | 3.92°N?, 36.225°E? | 8, 92 |
| 33 | Gx-5479 | 9660±235 | 11005 | Shell | Koobi Fora | 457 | Sand spits | Overflow | 4.077°N?, 36.347°E? | 8, 92 |
| 34 | KIA36857 | 9740±50 | 11182 | *Etheria elliptica* | Lake Turkana South Island | 459 | Abrasion platform | Overflow | 2.6611°N, 36.5879°E | 8 |
| 35 | RI-954 | 9940±260 | 11502 | Shell | Koobi Fora | 457 | Sand spits | Overflow | 4.077°N?, 36.347°E? | 8, 92 |

| | | | | | | | | | | | |
|---|---|---|---|---|---|---|---|---|---|---|---|
| 36 | KIA 36864 | 10025±55 | | 11524 | *Melanoides tuberculata* | South Island | 414 | Beach ridge | Lowstand, but nearly identical onset of Overflow | 2.6221°N, 36.5708°E | 8 |
| 37 | SUA-638 | 10720±150 | | 12630 | Shell | Koobi Fora | 416 | Beach sand | Closed basin | 3.94°N?, 36.2°E? | 8, 92 |
| ***Suguta*** | | | | | | | | | | | |
| 38 | KIA35809 | 6345±40 | 4405±75 | 5018 | ostracods | SE of Namarunu, central Suguta valley | 312 | 240 m below overflow level. | Closed basin, lowstand | 1.9128 N, 36.4651 E | 4, 6 |
| 39 | KIA36873 | 7850±50 | 5880±85 | 6700 | *Melanoides tuberculata* | N flank of Emuruangogolak volcanic center, S edge of Suguta valley | 565 | Maximum highstand shoreline (overflow level). | Overflow | 1.5608 N, 36.3342 E | 4, 6 |
| 40 | KIA36866 | 8510±60 | 6540±95 | 7449 | *Melanoides tuberculata* | Baragoi River mouth, S edge of Suguta valley | 557 | Maximum highstand shoreline (overflow level). | Overflow | 1.61890 N, 36.4609 E | 4, 6 |
| 41 | KIA33903 | 9365±50 | 7395±85 | 8222 | *Melanoides tuberculata* | "SANC" cinder cone, N edge of Suguta valley | 533 | Several shorelines from 492-560 m (560 m = maximum highstand). Carbonate-rich layer with snails and reworked material from 520-536 m. | Closed basin | 2.2725 N, 36.5782 E | 4, 6 |
| 42 | KIA33909 | 9775±45 | 7805±85 | 8601 | *Melanoides tuberculata* | Losetum cinder cone, S edge of Suguta valley | 519 | Wave-cut notch at 561 m represents maximum highstand shoreline and discontinuous carbonate-rich layers from 500-550 m. | Closed basin? | 1.5755 N, 36.3728 E | 4, 6 |
| 43 | KIA33901 | 9840±45 | 7870±85 | 8709 | *Melanoides tuberculata* | "SANC" cinder cone, N edge of Suguta valley | 481 | Several shorelines from 492-560 m (560 m = maximum highstand). Date from a carbonate-rich layer with snails and reworked material at the bottom of the cinder cone. | Closed basin? | 2.2724 N, 36.5781 E | 4, 6 |
| 44 | KIA33916 | 9970±45 | 8000±85 | 8854 | *Melanoides tuberculata* | Nakitoekirion, W edge of Suguta valley | 507 | Site shows a series of terraces from 490 m to maximum highstand level at reached at 570 m. Isolated lacustrine near-shore deposits probably contemporenous to maximum highstand shoreline. | Contemporenous to overflow | 1.9498 N, 36.3805 E | 4, 6 |

| | | | | | | | | | | | |
|---|---|---|---|---|---|---|---|---|---|---|---|
| 45 | KIA36872 | 9980±55 | 8010±90 | 8865 | *Melanoides tuberculata* | S of Tirr Tirr Plateau, E edge of Suguta valley | 559 | Maximum highstand shoreline (overflow level), expressed as terrace reaching 566 m. | Overflow | 1.7897 N, 36.5153 E | 4, 6 |
| 46 | KIA33917 | 10025±45 | 8055±85 | 8928 | *Melanoides tuberculata* | S of Namarunu volcanic center, W edge of Suguta valley | 564 | Maximum highstand shoreline (overflow level). | Overflow | 1.9555 N, 36.4156 E | 4, 6 |
| 47 | KIA33918 | 10750±50 | 8510±80 | 9503 | *Melanoides tuberculata* | S of Namarunu volcanic center, W edge of Suguta valley | 564 | Maximum highstand shoreline (overflow level). | Overflow | 1.9555 N, 36.4156 E | 4, 6 |
| 48 | KIA33913 | 10760±50 | 8520±80 | 9509 | *Melanoides tuberculata* | Namurinyang cinder cone, N edge of Suguta valley | 556 | Shallow lacustrine deposits (partly reworked) below maximum highstand shoreline expressed as a wavecut notch at 570 m on a steep-sided cinder cone. | Contemporenous to Overflow | 2.2479 N, 36.6137 E | 4, 6 |
| 49 | KIA37060 | 11495±60 | 9925±100 | 11409 | *Etheria elliptica* | Namruy area, SW edge of Suguta valley | 561 | Shell found on top of Namruy (LN08) section, likely reworked from maximum highstand shoreline elevation. | Overflow | 1.5378 N, 36.2251 E | 6, 9 |
| 50 | KIA33910 | 13725±60 | 12155±100 | 14031 | *Melanoides tuberculata* | Namruy area, SW edge of Suguta valley | 532 | Up to 60-m-thick deltaic deposits indicative of near- to offshore environments. Upper units at 564 m (maximum highstand), lower unites at 500 m. Fossil snails and fish throughout the deposit. | Closed basin | 1.5373 N, 36.2256 E | 4, 6 |
| 51 | KIA33907 | 13900±60 | 12330±100 | 14378 | *Etheria elliptica* | Namruy area, SW edge of Suguta valley | 530 | Up to 60-m-thick deltaic deposits indicative of near- to offshore environments. Upper units at 564 m (maximum highstand), lower unites at 500 m. Fossil snails and fish throughout the deposit. | Closed basin | 1.5373 N, 36.2256 E | 4, 6 |
| 52 | UQ1017 | 9300±400 | 7680±405 | 8558 | *Melanoides tuberculata* | Rupa, W-central Suguta Valley | 484 | $^{14}C$ date normalized with -25‰ to 9650±400 yr BP. Diatomites rich in fossil *Tilapia* sp. (=*Oreochromis* sp.). | Approximate age of fish fossils | 1.937 N, 36.395 E | 26 |

| | | | | | | | | | | | |
|---|---|---|---|---|---|---|---|---|---|---|---|
| 53 | Birm-585 a | 11250±220 | 9680±235 | 11030 | *Etheria elliptica* | Naserkalia, close to Suguta River, south of Suguta Valley | n.a. | Coarse gravel of beach deposit rich in catfish and perch overlying diatomaceous sediments. (catfish = Siluriformes; perch = *Lates* sp.). | Age of fish fossils | 1.5333 N, 36.2666 E | 24, 63 |
| 54 | Birm-585 b | 10570±300 | 8330±305 | 9272 | *Etheria elliptica* | Naserkalia, close to Suguta River, south of Suguta Valley | n.a. | Coarse gravel of beach deposit rich in catfish and perch overlying diatomaceous sediments. (catfish = Siluriformes; perch = *Lates* sp.). | Age of fish fossils | 1.5333 N, 36.2666 E | 24, 63 |
| ***Baringo-Bogoria*** | | | | | | | | | | | |
| 55 | Birm-883 a | 10560±170 | 6580±170 | 7470 | *Melanoides tuberculata* inner shell | NW shore of Lake Bogoria | 992.6 | Sample 2.95-3.10 m above 1976 Bogoria lake level. | No overflow (Lake Bogoria Closed/isolated) | 0.300 N, 36.078 E | 1 |
| 56 | Birm-883 b | 10320±150 | 6340±150 | 7245 | *Melanoides tuberculata* middle shell | NW shore of Lake Bogoria | 992.6 | Sample 2.95-3.10 m above 1976 Bogoria lake level. | No overflow (Lake Bogoria Closed/isolated) | 0.300 N, 36.078 E | 1 |
| 57 | Birm-542 a | 13850±430 | 9870±430 | 11397 | *Melanoides tuberculata* middle shell | W shore Lake Baringo, Kobwob Murren | 987 | Beach/near-shore deposit 15.85 m above 1969/1973 Baringo lake level, maximum shoreline. Sediments contain fossil *Tilapia* (*Oreochromis*) sp. and *Lates niloticus*. | Overflow of Baringo (connected Lake Baringo Bogoria), Age of fish fossils | 0.633 N, 36.016 E | 1, 27, 63 |
| 58 | Birm-542 b | 13670±320 | 9690±320 | 11087 | *Melanoides tuberculata* inner shell | W shore Lake Baringo, Kobwob Murren | 987 | Beach/near-shore deposit 15.85 m above 1969/1973 Baringo lake level, maximum shoreline. Sediments contain fossil *Tilapia* (*Oreochromis*) sp. and *Lates niloticus*. | Overflow of Baringo (connected Lake Baringo Bogoria), Age of fish fossils | 0.633 N, 36.016 E | 1, 27, 63 |
| 59 | Birm-541 a | 11870±310 | 7890±310 | 8783 | *Melanoides tuberculata* inner shell | Logumukum (Loboi Plain), halfway between Lakes Bogoria and Baringo | 987 | Emerged beach 15.85 m above 1969/1973 Baringo lake level, maximum shoreline. Sediments contain fossil *Labeo* sp. | Overflow of Bogoria and Baringo (connected Lake Baringo-Bogoria), Age of fish fossils | 0.450 N, 36.083 E | 1, 27, 63 |

| | | | | | | | | | | | |
|---|---|---|---|---|---|---|---|---|---|---|---|
| 60 | Birm-541 b | 10860±280 | 6880±280 | 7742 | *Melanoides tuberculata* middle shell | Logumukum (Loboi Plain), halfway between Lakes Bogoria and Baringo | 987 | Emerged beach 15.85 m above 1969/1973 Baringo lake level, maximum shoreline. Sediments contain fossil *Labeo* sp. | Overflow of Bogoria and Baringo (connected Lake Baringo-Bogoria), Age of fish fossils | 0.450 N, 36.083 E | 1, 27, 63 |
| 61 | Birm-544 a | 12260±280 | 8280±280 | 9213 | *Melanoides tuberculata* inner shell | W of Lake Baringo | ca. 986.5 | Silt 15.54 m above 1969/1973 Baringo lake level. | Overflow of Baringo (connected Lake Baringo Bogoria) | 0.583 N, 36.000 E | 1, 63 |
| 62 | Birm-544 b | 12600±280 | 8620±280 | 9676 | *Melanoides tuberculata* middle shell | W of Lake Baringo | ca. 986.5 | Silt 15.54 m above 1969/1973 Baringo lake level. | Overflow of Baringo (connected Lake Baringo Bogoria) | 0.583 N, 36.000 E | 1, 63 |
| 63 | Birm-545 a | 9940±250 | 5960±250 | 6813 | *Melanoides tuberculata* inner shell | W of Lake Baringo | ca. 981 | Intermediate shoreline 10.06 m above 1969/1973 Baringo lake level. | Overflow of Baringo | 0.592 N, 36.000 E | 1, 63 |
| 64 | Birm-545 b | 10810±270 | 6830±270 | 7696 | *Melanoides tuberculata* middle shell | W of Lake Baringo | ca. 981 | Intermediate shoreline 10.06 m above 1969/1973 Baringo lake level. | Overflow of Baringo | 0.592 N, 36.000 E | 1, 63 |
| 65 | Birm-543 a | 7620±180 | 3640±180 | 3978 | *Unio* sp. & *Viviparus*? sp. inner shells | W of Lake Baringo | ca. 975 | Beach/near-shore deposit 3.66 m above 1969/1973 Baringo lake level. | No overflow (Lake Baringo Closed/isolated) | 0.603 N, 36.014 E | 63 |
| 66 | Birm-543 b | 8460±180 | 4480±180 | 5127 | *Unio* sp. & *Viviparus*? sp. middle shells | W of Lake Baringo | ca. 975 | Beach/near-shore deposit 3.66 m above 1969/1973 Baringo lake level. | No overflow (Lake Baringo Closed/isolated) | 0.603 N, 36.014 E | 63 |
| ***Menengai*** | | | | | | | | | | | |
| 67 | KIA28136 | 4555±146 | | 5207 | Charcoal in diatomite | Menengai caldera | 1807 | Sediments partly laminated, indicating deep lake. | Minimum age of overflow | 0.1631 S, 36.0960 E | 30 |
| 68 | Poz-59047 | 10440±210 | | 12249 | Plant remains in basal lake sediments (diatomite) | Menengai caldera | 1742 | Caldera lake coeval with phreatomagmatic "Ruplax tuff", indicating water-filled caldera. | Possible onset of overflow | 0.1816 S, 36.0735 E | 29, 30 |

***Nakuru-Elmenteita***

| | | | | | | | | | |
|---|---|---|---|---|---|---|---|---|---|
| 69 | GX-4320 | 1370±140 | 1282 | n.a. | Nderit Drift, Fm. 4 | n.a. (~1830-1850) | Incision and filling of Nderit River Gorge into L. Nakuru basin floor (alluvial canyon fillings); post high lake stage. | Closed basin(s), likely separate lakes | 0.5191 S, 36.1038 E | 99 |
| 70 | GX-4419 | 3135±155 | 3332 | n.a. | Nderit Drift, Fm. 4 | n.a. (~1830-1850) | Incision and filling of Nderit River Gorge into L. Nakuru basin floor (alluvial canyon fillings); post high lake stage. | Closed basin(s), likely separate lakes | 0.5191 S, 36.1038 E | 99 |
| 71 | N-821 | 3540±120 | 3832 | charcoal | Nderit Drift, Fm. 4 | n.a. (~1830-1850) | Brown, alluvial silts, upper member of formation. Lake Nakuru below +50 m lake level. | Closed basin(s), likely separate lakes | 0.5191 S, 36.1038 E | 36, 94 |
| 72 | n.a. | 3735±255 | 4113 | bulk sediment | Lake Nakuru core | n.a. | Lake Nakuru moderately fresh and probably confined to its own basin (same with L. Elmenteita). Mostly a dry lake during this time. | Closed basin(s), likely separate lakes | 0.3701 S, 36.0895 E | 31 |
| 73 | GX-4470 | 4040±140 | 4535 | n.a. | Nderit Drift, Fm. 4 | n.a. (~1830-1850) | Incision and filling of Nderit River Gorge into L. Nakuru basin floor (alluvial canyon fillings); post high lake stage. | Closed basin(s), likely separate lakes | 0.5191 S, 36.1038 E | 99, 100 |
| 74 | I-5554 | 6490±125 | 7394 | bulk sediment | Lake Elmenteita core | n.a. | Lake Elmenteita was moderately alkaline, its level at the time above the modern lake level, but no overflow. Marks beginning of rapidly declining lake levels. | Closed basin | 0.4424 S, 36.2439 E | 31 |
| 75 | GX-4317 | 7005±175 | 7839 | charcoal | Nderit Drift, Fm. 3, Mbr. 8 | n.a. (~1830-1850) | Incision and filling of Nderit River Gorge into L. Nakuru basin floor asscoiated with intermediate level of Lake Nakuru. | Closed basin | 0.5191 S, 36.1038 E | 99, 100 |
| 76 | GX-4215 | 7105±180 | 7932 | charcoal | Nderit Drift, Fm. 3, Mbr. 8 | n.a. (~1830-1850) | Incision and filling of Nderit River Gorge into L. Nakuru basin floor asscoiated with intermediate level of Lake Nakuru. | Closed basin | 0.5191 S, 36.1038 E | 99, 100 |
| 77 | UCLA-1757 | 7410±160 | 8216 | buried hominin | Bromhead's Site | 1867 | 104 m above present level of Lake Nakuru. Post overflow highstand. | Closed basin | 0.4910 S, 36.0827 E | 101, 102 |

| | | | | | | | | | | |
|---|---|---|---|---|---|---|---|---|---|---|
| 78 | GX-0290 | 8095±190 | 9005 | charcoal | Gamble's Cave, Layer 4 | ~1934 | Excavation directly above beach deposits from last phase of overflow of L. Nakuru, indicate stable lake level. (Comment: otherwise the charcoal should have been deposited at lower elevation) | Overflow (merged lakes) | 0.5513 S, 36.0915 E | 99 |
| 79 | GX-0289 | 8245±175 | 9198 | charcoal | Gamble's Cave, Layer 4 | ~1934 | Excavation directly above beach deposits from last phase of overflow of L. Nakuru, indicate stable lake level. (Comment: otherwise the charcoal should have been deposited at lower elevation) | Overflow (merged lakes) | 0.5513 S, 36.0915 E | 99 |
| 80 | KIA13109 | 8440±40 | 9474 | snail shells | Lemulug Volcano | 1932 | Highest outcrops of early Holocene lacustrine sediments corresponding to maximum highstand shoreline at Katerit Volcano. | Overflow (merged lakes) | 0.5005 S, 36.2436 E | 103 |
| 81 | GX-0288 | 8510±180 | 9508 | charcoal | Gamble's Cave, Layer 4 | ~1934 | Excavation directly above beach deposits from last phase of overflow of L. Nakuru, indicate stable lake level. (Comment: otherwise the charcoal should have been deposited at lower elevation) | Overflow (merged lakes) | 0.5513 S, 36.0915 E | 99 |
| 82 | n.a. | 8640±215 | 9703 | organic mud | Lake Nakuru core | n.a. | Open, merged freshwater lake. Dominance of *Stephanodiscus rotula*, indicating deep, (seasonally) stratified lake. Probably surface outlet to the north. | Overflow (merged lakes) | 0.3701 S, 36.0895 E | 31, 32 |
| 83 | I-5179 | 8740±190 | 9815 | organic mud | Lake Elmenteita core | n.a. | Enlarged, very fresh lake. Dominance of *Stephanodiscus rotula*, indicating a deep, (seasonally) stratified lake. Probably surface outlet to the north. Open merged freshwater lake. | Overflow (merged lakes) | 0.4424 S, 36.2439 E | 31, 32, 94 |

| | | | | | | | | | | |
|---|---|---|---|---|---|---|---|---|---|---|
| 84 | L-1201 | 9650±250 | 10997 | mixed shells | Katerit Volcano | 1888 | Radiocarbon date of shell layer at 1888 m associated with maximum highstand shoreline at 1943 m present on the same volcano (180 m above present lake level (= overflow level). | Overflow (merged lakes) | 0.5074 S, 36.2700 E | 94, 104 |
| 85 | n.a. | 10340±150 | 12154 | organic mud | Lake Nakuru core | n.a. | Beginning of Lake Nakuru's major freshening phase and merging of Lakes Nakuru and Elmenteita. Very fresh, start of Gamble's Cave shoreline. Open, merged freshwater lake. | Overflow (merged lakes) | 0.3701 S, 36.0895 E | 31, 32 |
| 86 | KIA13108 | 10390±45 | 12267 | snail shells | E of Lake Elmenteita | 1894 | Near shoreline deposit (beach gravel) dated at 2.5 m below surface. | Closed basin(s) | 0.4270 S, 36.2629 E | 103 |
| 87 | GX-4214 | 10685±270 | 12523 | n.a. | Nderit drift, Fm 3, Member 7 | n.a. (~1830-1850) | Incising of the of Nderit River Gorge into the Lake Nakuru basin floor, accompanied by deposition of alluvial sediments. Preceding L. Nakuru overflow phase. | Closed basin(s) | 0.5191 S, 36.1038 E | 99 |
| 88 | N-822-3 | 12000±215 | 13893 | charcoal | Nderit drift Unit b | n.a. (~1830-1850) | Ponded valley and deltaic deposits, indicating a transgressive phase of ca. 60 m above modern level of Lake Nakuru. | Closed basin(s) | 0.5191 S, 36.1038 E | 36, 94, 105 |
| 89 | GX-4215 | 12065±365 | 14089 | n.a. | Nderit drift, Fm 3, Member 7 | n.a. (~1830-1850) | Incising of the of Nderit River Gorge into the Lake Nakuru basin floor, accompanied by deposition of alluvial sediments. Preceding L. Nakuru overflow phase. | Closed basin(s) | 0.5191 S, 36.1038 E | 99 |
| 90 | I-5062 | 12160±170 | 14091 | charcoal | Nderit drift Unit b | n.a. (~1830-1850) | Ponded valley and deltaic deposits, indicating a transgressive phase of ca. 60 m above modern level of Lake Nakuru. | Closed basin(s) | 0.5191 S, 36.1038 E | 36, 94 |
| 91 | I-5178 | 12200±180 | 14176 | organic mud | Lake Elmenteita core | n.a. | Early weak phase of freshening of Lake Elmenteita, before receding again. Lakes remains closed. | Closed basin(s) | 0.4424 S, 36.2439 E | 31 |

| | | | | | | | | | | |
|---|---|---|---|---|---|---|---|---|---|---|
| 92 | N-822-2 | 12200±220 | 14210 | charcoal | Nderit drift Unit b | n.a. (~1830-1850) | Ponded valley and deltaic deposits, indicating a transgressive phase of ca. 60 m above modern level of Lake Nakuru. | Closed basin(s) | 0.5191 S, 36.1038 E | 36, 94, 105 |
| 93 | N-822-1 | 12300±220 | 14391 | charcoal | Nderit drift Unit b | n.a. (~1830-1850) | Ponded valley and deltaic deposits, indicating a transgressive phase of ca. 60 m above modern level of Lake Nakuru. | Closed basin(s) | 0.5191 S, 36.1038 E | 36, 94, 105 |
| 94 | n.a. | 12850±190 | 15333 | bulk sediment | Nakuru core | n.a. | Diatom assemblages indicate brief shift from low lake level and high alkalinity to more dilute conditions and higher lake level followed by return to lower levels until ~10500 $^{14}$C BP. | Closed basin(s) | 0.3701 S, 36.0895 E | 31, 32 |
| ***Marula Rockshelter*** | | | | | | | | | | |
| 95 | GX-6763-A | 7195±260 | 8026 | n.a. | Marula Rock Shelter | ~2000 | Dated deposit from which a rich fauna, including one lower 2$^{nd}$ molar from *Hylochoerus meinertzhageni* was recovered. | Date of fossil fauna | 0.6505 S, 36.3388 E | 66, 106, 107 |
| ***Naivasha*** | | | | | | | | | | |
| 96 | n.a. | 1740±95 | 1660 | bulk sediment | Lake Naivasha main basin core | n.a. | Lowstand. Beginning of modern lake character. | Closed basin | 0.7757 S, 36.3538 E | 32 |
| 97 | Y-1436 | 3000±60 | 3183 | bulk sediment | Crescent Island Crater core | n.a. | Date from above dessication layer. Lake probably dried out completely for a short time. | Closed basin | 0.7643 S, 36.4097 E | 34, 94, 108 |
| 98 | Y-1769 | 3040±60 | 3240 | bulk sediment | Crescent Island Crater core | n.a. | Date from above dessication layer. Lake probably dried out completely for a short time. | Closed basin | 0.7643 S, 36.4097 E | 34, 94 |
| 99 | n.a. | 4145±100 | 4668 | bulk sediment | Lake Naivasha main basin core | n.a. | Shallow conditions after rapid lake level decline. | Closed basin | 0.7757 S, 36.3538 E | 32 |
| 100 | Y-1339 | 5650±120 | 6452 | bulk sediment | Crescent Island Crater core | n.a. | The date marks the termination of enlarged, deep lake phase with an active surface outlet. Deep-lake diatom indicators decline/dissapaer after this time, while organic matter content increases. | Overflow | 0.7643 S, 36.4097 E | 34, 94, 108 |

| | | | | | | | | | | | |
|---|---|---|---|---|---|---|---|---|---|---|---|
| 101 | I-1340 | 9200±160 | | 10399 | bulk sediment | Crescent Island Crater core | n.a. | Fairly stable, enlarged and deep freshwater lake which was overflowing through its surface outlet the Ol Njorowa gorge. Occurrence of *Stephanodiscus* in the diatom assemblage indicates a deep, possibly stratified lake . | Overflow | 0.7643 S, 36.4097 E | 34, 94, 108 |
| 102 | n.a. | 9670±160 | | 10996 | bulk sediment | Lake Naivasha main basin core | n.a. | Begin highstand phase with continuously high water levels. Diatom assemblages dominated by *Melosira agassizii*. | Overflow | 0.7757 S, 36.3538 E | 32 |
| 103 | UCLA-1741 | 10850±330 | | 12748 | hominin bones | Prehistoric site close to Naivasha town. | n.a. | Drowned hominin found in bottom of lacustrine silt layer close to (former) lake shore. Skeleton located 10 m below highest Naivasha lake level. | Closed basin | 0.7333 S, 36.4333 E | 33, 102 |
| 104 | n.a. | 12270±180 | | 14318 | bulk sediment | Lake Naivasha main basin core | n.a. | Diatom assemblages indicate low lake level (below and close to modern level) and relatively fresh to more alkaline conditions. | Closed basin | 0.7757 S, 36.3538 E | 32 |
| ***Magadi-Natron*** | | | | | | | | | | | |
| 105 | UQ938 | 7000±100 | 4950±120 | 5703 | stromatolite | Magadi-Natron basin | n.a. | Regressive shoreline | Two separate lakes | n.a. | 35 |
| 106 | UQ982 | 7880±450 | 5830±455 | 6662 | stromatolite | Magadi-Natron basin | n.a. | Regressive shoreline | Two separate lakes | n.a. | 35 |
| 107 | UQ959 | 8100±200 | 6050±210 | 6914 | stromatolite | Magadi-Natron basin | n.a. | Regressive shoreline | Two separate lakes | n.a. | 35 |
| 108 | UQ920 | 8500±100 | 6450±120 | 7360 | stromatolite | Magadi-Natron basin | n.a. | Regressive shoreline | Two separate lakes | n.a. | 35 |
| 109 | UQ936 | 9650±100 | 7600±120 | 8406 | stromatolite | NW of L. Magadi | 650 | Highstand shoreline | Single merged lake | 1.770 S, 36.245 E | 35 |
| 110 | UQ907 | 9650±200 | 7600±210 | 8420 | stromatolite | Magadi-Natron basin | 645-656 | Highstand shoreline | Single merged lake | n.a. | 35 |
| 111 | UQ904 | 9700±200 | 7650±210 | 8481 | stromatolite | NE of L. Natron | 656 | Highstand shoreline | Single merged lake | 2.215 S, 36.153 E | 35 |
| 112 | UQ953 | 9710±100 | 7660±120 | 8469 | stromatolite | Magadi-Natron basin | 645-656 | Highstand shoreline. Companion sample to UQ951: top of stromatolite | Single merged lake | n.a. | 35 |
| 113 | UQ910 | 9800±100 | 7750±120 | 8557 | stromatolite | W of L. Magadi | 656 | Highstand shoreline | Single merged lake | 1.804 S, 36.261 E | 35 |

| | | | | | | | | | | | |
|---|---|---|---|---|---|---|---|---|---|---|---|
| 114 | UQ932 | 9800±200 | 7750±210 | 8599 | stromatolite | N of L. Natron | 656 | Highstand shoreline | Single merged lake | 1.997 S, 36.049 E | 35 |
| 115 | UQ894 | 9850±100 | 7800±120 | 8621 | stromatolite | NE of L. Natron | 656 | Highstand shoreline | Single merged lake | 2.172 S, 36.103 E | 35 |
| 116 | UQ951 | 9860±150 | 7810±165 | 8660 | stromatolite | Magadi-Natron basin | 645-656 | Highstand shoreline. Companion sample to UQ953: bottom of stromatolite | Single merged lake | n.a. | 35 |
| 117 | UQ673 | 9900±150 | 7850±165 | 8709 | stromatolite | SW of L. Natron | 645 | Highstand shoreline | Single merged lake | 2.543 S, 35.891 E | 35 |
| 118 | UQ943 | 10090±140 | 8040±155 | 8919 | stromatolite | Magadi-Natron basin | 645-656 | Highstand shoreline | Single merged lake | n.a. | 35 |
| 119 | UQ614 | 10250±110 | 8200±125 | 9171 | stromatolite | Magadi-Natron basin | 645-656 | Highstand shoreline | Single merged lake | n.a. | 35 |
| 120 | UQ659 | 10300±105 | 8250±120 | 9227 | stromatolite | Magadi-Natron basin | 645-656 | Highstand shoreline | Single merged lake | n.a. | 35 |
| 121 | UQ627 | 10300±115 | 8250±130 | 9224 | stromatolite | Magadi-Natron basin | 645-656 | Highstand shoreline | Single merged lake | n.a. | 35 |
| 122 | UQ652 | 10300±130 | 8250±145 | 9218 | stromatolite | NW of L. Natron | 656 | Highstand shoreline | Single merged lake | 2.221 S, 35.968 E | 35 |
| 123 | UQ899 | 10300±200 | 8250±210 | 9190 | stromatolite | NE of L. Magadi | 656 | Highstand shoreline | Single merged lake | 1.806 S, 36.322 E | 35 |
| 124 | UQ564 | 10360±160 | 8310±170 | 9270 | stromatolite | W of L. Natron | 645 | Highstand shoreline | Single merged lake | 2.308 S, 35.956 E | 35 |
| 125 | UQ877 | 10460±120 | 8410±135 | 9381 | stromatolite | NE of L. Natron | 656 | Highstand shoreline | Single merged lake | 2.138 S, 36.060 E | 35 |
| 126 | UQ661 | 10560±125 | 8510±140 | 9502 | stromatolite | Magadi-Natron basin | 645-656 | Highstand shoreline | Single merged lake | n.a. | 35 |
| 127 | UQ933 | 10650±100 | 8600±120 | 9617 | stromatolite | Magadi-Natron basin | 645-656 | Highstand shoreline | Single merged lake | n.a. | 35 |
| 128 | UQ669 | 10660±115 | 8610±130 | 9636 | stromatolite | Magadi-Natron basin | 645-656 | Highstand shoreline | Single merged lake | n.a. | 35 |
| 129 | UQ911 | 10750±200 | 8700±210 | 9775 | stromatolite | W of L. Magadi | 650 | Highstand shoreline | Single merged lake | 1.926 S, 36.183 E | 35 |
| 130 | UQ886 | 10850±300 | 8800±305 | 9887 | stromatolite | Magadi-Natron basin | 645-656 | Highstand shoreline | Single merged lake | n.a. | 35 |
| 131 | UQ888 | 10950±200 | 8900±210 | 9975 | stromatolite | Magadi-Natron basin | 645-656 | Highstand shoreline | Single merged lake | n.a. | 35 |
| 132 | UQ909 | 11350±200 | 9300±210 | 10536 | stromatolite | W of L. Magadi | 656 | Highstand shoreline | Single merged lake | 1.804 S, 36.261 E | 35 |
| 133 | UQ618 | 11375±140 | 9325±155 | 10551 | stromatolite | NW of L. Natron | 645-656 | Highstand shoreline | Single merged lake | 2.157 S, 35.980 E | 35 |

| | | | | | | | | | | | |
|---|---|---|---|---|---|---|---|---|---|---|---|
| 134 | UQ917 | 11500±100 | 9450±120 | 10732 | stromatolite | N of L. Natron | 656 | Highstand shoreline | Single merged lake | 1.997 S, 36.049 E | 35 |
| 135 | UQ587 | 11640±100 | 9590±120 | 10928 | stromatolite | SW of L. Natron | 650 | Highstand shoreline | Single merged lake | 2.518 S, 35.903 E | 35 |
| 136 | UQ636 | 11950±115 | 9900±130 | 11399 | stromatolite | Magadi-Natron basin | 645-656 | Highstand shoreline | Single merged lake | n.a. | 35 |
| 137 | UQ767 | 12025±175 | 9975±185 | 11540 | stromatolite | Magadi-Natron basin | 645-656 | Highstand shoreline | single merged lake | n.a. | 35 |
| 138 | UQ930 | 12450±100 | 10400±120 | 12262 | stromatolite | S of L. Magadi | 645 | Highstand shoreline | Single merged lake | 1.995 S, 36.266 E | 35 |
| 139 | UQ927 | 4560±400 | 2510±405 | 2581 | *Alcolapia* fish bone | SE of Lake Magadi | 605 | High Magadi beds | Age of fish fossils, two separate lakes | 1.94 S, 36.29 E | 35 |
| 140 | N-862 | 9120±170 | 7070±180 | 7897 | Organic fraction of carbonaceous fish fossil lamina (*Alcolapia*) | Dry Lagoon SE of Lake Magadi | n.a. | High Magadi Beds: a few meters above 604 m lake level | Age of fish fossils, two separate lakes | 1.9833 S, 36.2666 E | 36, 94, 105 |
| 141 | n.a. | 10000±200[2] | 7950±210 | 8830 | *Alcolapia* fish bone | SE and NW side of Lake Magadi | ca. 624 and 629 | 20 and 25 m above 1996 Magadi lake level | Age of fish fossils, single merged lake | 1.9883 S, 36.2686 E; 1.8087 S, 36.2594 E | 3, H. Tichy pers. communication |

[1] ? under geographic coordinates denotes uncertain location for Lake Turkana region as given in ref. 8.
[2] sigma range assumed (not reported in original paper).

**Table S3.** Radiocarbon dates from study sites in the South Kenya Rift.

| Site | Lab ID | Outcrop ID | Depth below top of out-crop (cm) | Deposi-tional environ-ment | Dated material | $^{14}C$ date (yr BP) | Reservoir effect (yr±1σ)[1] | Reservoir corrected $^{14}C$ date (yr BP)[1, 2] | Calibrated median age (cal. yr BP) | Calibrated age 2σ error bounds (cal. yr BP) | Geographic coordi-nates (°) | Eleva-tion (m) |
|---|---|---|---|---|---|---|---|---|---|---|---|---|
| Siriata | Poz-78488 | KOO15-1A[3] | 0 | lacustrine | *Corbicula* sp. | 11080 ± 60 | | 6985 ± 190 | 7824 | 7495 - 8179 | 1.99588 S 36.35392 E | 809.1 |
| Siriata | Poz-78489 | KOO15-1A | 100 | lacustrine | *Corbicula* sp. | 12210 ± 60 | | 8155 ± 190 | 9086 | 8598 - 9493 | 1.99588 S 36.35392 E | 808.1 |
| Siriata | Poz-75380 | KOO15-1A | 180 | lacustrine | *Melanoides tuberculata* | 12890 ± 60 | | 8795 ± 190 | 9868 | 9453 - 10291 | 1.99588 S 36.35392 E | 807.3 |
| Siriata | Poz-78491 | KOO15-1A | 270 | lacustrine | *Corbicula* sp. | 13580 ± 110 | | 9485 ± 210 | 10788 | 10233 - 11259 | 1.99588 S 36.35392 E | 806.4 |
| Siriata | Poz-89468 | KOO15-3E[4] | 430 | lacustrine | charcoal | 8680 ± 250 | | - | 9753 | 9034 - 10370 | 1.96413 S 36.36753 E | 806.7 |
| Siriata | Poz-78492 | KOO15-3E | 430 | lacustrine | *Corbicula* sp. | 12660 ± 70 | 3980 ± 260 | 8565 ± 195 | 9596 | 9128 - 10169 | 1.96413 S 36.36753 E | 806.7 |
| Siriata | Poz-78493 | KOO15-3E | 490 | lacustrine | *Corbicula* sp. | 12900 ± 90 | | 8805 ± 200 | 9879 | 9456 - 10381 | 1.96413 S 36.36753 E | 806.1 |
| Siriata | Poz-78495 | KOO15-3E | 570 | lacustrine | *Melanoides tuberculata* | 13040 ± 70 | | 8945 ± 195 | 10019 | 9544 - 10501 | 1.96413 S 36.36753 E | 805.3 |
| Siriata | Poz-78423 | KOO15-3E | 666 | lacustrine | *Melanoides* fragments | 14060 ± 180 | | 9965 ± 255 | 11541 | 10735 - 12396 | 1.96413 S 36.36753 E | 804.3 |
| Siriata | Poz-78442 | KOO15-3E | 700 | tephra | Charcoal | 7540 ± 50 | | - | 8361 | 8205 - 8421 | 1.96413 S 36.36753 E | 804.0 |
| Siriata | Poz-89664 | KOO15-3E | 713 | lacustrine | Charcoal | 10030 ± 150 | | - | 11595 | 11194 - 12112 | 1.96413 S 36.36753 E | 803.9 |
| Siriata | Poz-78390 | KOO15-3E | 750 | silicious paleosol | Charcoal | 10440 ± 100 | | - | 12320 | 12005 - 12628 | 1.96413 S 36.36753 E | 803.5 |
| Siriata | Poz-78496 | KOO15-3D | 22-23.5 | lacustrine | *Corbicula* sp. | 12770 ± 70 | | 8675 ± 195 | 9745 | 9302 - 10225 | 1.96375 S 36.36785 E | 812.9 |
| Siriata | Poz-78497 | KOO15-3D | 40-44 | lacustrine | *Corbicula* sp. | 12670 ± 60 | | 8575 ± 190 | 9610 | 9136 - 10166 | 1.96375 S 36.36785 E | 812.7 |
| Siriata | Poz-78498 | KOO15-6B[5] | 575 | lacustrine | *Corbicula* sp. | 13710 ± 70 | | 9615 ± 195 | 10939 | 10300 - 11600 | 2.03814 S 36.36776 E | 809.6 |
| Siriata | Poz-78499 | KOO15-6B | 630 | lacustrine | *Corbicula* sp. | 13600 ± 70 | 4380 ± 120 | 9505 ± 195 | 10815 | 10267 - 11239 | 2.03814 S 36.36776 E | 809.0 |
| Siriata | Poz-78392 | KOO15-6B | 630 | lacustrine | Charcoal | 9220 ± 100 | | - | 10403 | 10217 - 10655 | 2.03814 S 36.36776 E | 809.0 |
| Siriata | Poz-78500 | KOO15-6B | 650 | lacustrine | *Corbicula* sp. | 13240 ± 70 | 3930 ± 120 | 9145 ± 195 | 10327 | 9682 - 11065 | 2.03814 S 36.36776 E | 808.8 |
| Siriata | Poz-78391 | KOO15-6B | 650 | lacustrine | Charcoal | 9310 ± 100 | | - | 10505 | 10244 - 10745 | 2.03814 S 36.36776 E | 808.8 |
| Kwenia | Poz-89660 | KWE16-1C[6] | ca. 15 | lacustrine | Fish bone | 18030 ± 100 | | 13935 ± 205 | 16889 | 16289 - 17478 | 1.83197 S 36.49121 E | 1093.1 |
| Ol Keju Nyiro River | Poz-89658 | RI[7] | 580 | fluvial / alluvial | Bulk sediment | 6130 ± 40 | | - | 7025 | 6912 - 7160 | 1.59300 S 36.40646 E | 921.3 |
| Ol Keju Nyiro River | Poz-89659 | RII[7] | 180 | fluvial / alluvial | Bulk sediment | 3770 ± 40 | | - | 4139 | 3986 - 4282 | 1.59297 S 36.40684 E | 925.3 |

[1] sigma range calculated by root mean square and rounded to nearest 5.
[2] reservoir correction to all carbonate-derived $^{14}C$ dates by subtraction of 4095±180 $^{14}C$ years.
[3] see photo 6 in Fig. S4.
[4] see photo 7 in Fig. S4.
[5] see photo 8 in Fig. S4.
[6] see photo 9 in Fig. S4.
[7] see photo 10 in Fig. S4.

**Table S4.** Single-crystal incremental heating ('SCIH') analytical data.

| | | Relative Isotopic Abundances | | | | | | | | | | | | Derived Results | | | | | | | | | | Inverse Isochron Data | | | | |
|---|---|---|---|---|---|---|---|---|---|---|---|---|---|---|---|---|---|---|---|---|---|---|---|---|---|---|---|---|
| Lab ID# | Watts | J ( X $10^{-3}$) ± 1s | | $^{40}$Ar ±1s | | $^{39}$Ar ±1s | | $^{38}$Ar ±1s | | $^{37}$Ar ±1s | | $^{36}$Ar ±1s | | $^{39}$Ar Mol X $10^{-16}$ | $^{39}$Ar % of total | Ca/K ±1s | | $^{40}$Ar*/$^{39}$Ar ±1s | | %$^{40}$Ar* | Age (ka) ±1s | | w/±J ±1s | $^{36}$Ar/$^{40}$Ar ±%1s | | $^{39}$Ar/$^{40}$Ar ±%1s | | $^{36}$Ar/$^{39}$Ar Er. Corr. |
| OLOR16/SKG-1pB1 | | | | | | | | | | | | | | | | | | | | | | | | | | | | |
| 27878-01A | 1.0 | 0.02240 | 0.00004 | 5383.2 | 60.7 | 130.8 | 3.3 | 19.1 | 1.8 | -2.9 | 4.6 | 20.2 | 0.9 | 0.06 | 1.1 | -0.157 | 0.247 | -5.065 | 2.106 | -12.3 | -207.2 | 86.1 | 86.1 | 0.00376 | 4.58 | 0.02430 | 2.79 | 0.0996 |
| 27878-01B | 1.3 | 0.02240 | 0.00004 | 7747.9 | 60.7 | 521.5 | 4.9 | 17.1 | 1.5 | -3.7 | 4.6 | 26.6 | 1.0 | 0.24 | 4.4 | -0.051 | 0.062 | -0.394 | 0.579 | -2.7 | -16.1 | 23.7 | 23.7 | 0.00344 | 3.80 | 0.06735 | 1.22 | 0.1319 |
| 27878-01C | 1.9 | 0.02240 | 0.00004 | 13631.4 | 61.9 | 1427.3 | 7.7 | 34.0 | 2.2 | -4.8 | 4.6 | 41.2 | 1.0 | 0.66 | 12.0 | -0.024 | 0.023 | 0.937 | 0.218 | 9.8 | 38.3 | 8.9 | 8.9 | 0.00302 | 2.52 | 0.10476 | 0.71 | 0.1159 |
| 27878-01D | 2.6 | 0.02240 | 0.00004 | 8022.0 | 60.1 | 2978.2 | 7.5 | 43.6 | 1.9 | 5.9 | 4.6 | 15.0 | 0.8 | 1.38 | 25.1 | 0.014 | 0.011 | 1.190 | 0.084 | 44.2 | 48.7 | 3.4 | 3.4 | 0.00187 | 5.47 | 0.37146 | 0.79 | 0.1299 |
| 27878-01E | 3.7 | 0.02240 | 0.00004 | 7755.7 | 60.5 | 5946.0 | 13.6 | 88.5 | 2.4 | -5.8 | 4.7 | 18.1 | 0.9 | 2.75 | 50.2 | -0.007 | 0.006 | 0.393 | 0.046 | 30.1 | 16.1 | 1.9 | 1.9 | 0.00234 | 5.00 | 0.76716 | 0.81 | 0.1497 |
| 27878-01F | 5.1 | 0.02240 | 0.00004 | 1433.0 | 57.3 | 841.6 | 5.2 | 7.5 | 1.5 | 9.3 | 4.8 | 3.6 | 0.8 | 0.39 | 7.1 | 0.078 | 0.040 | 0.443 | 0.288 | 26.0 | 18.1 | 11.8 | 11.8 | 0.00248 | 22.61 | 0.58765 | 4.04 | 0.1747 |
| OLOR16/SKG-1pB1 - Aliquot #2 | | | | | | | | | | | | | | | | | | | | | | | | | | | | |
| ***27878-02A*** | 1.0 | 0.02240 | 0.00004 | 40873.7 | 69.2 | 176.2 | 3.3 | 34.8 | 1.8 | -3.3 | 4.8 | 135.8 | 1.3 | 0.08 | 2.7 | -0.132 | 0.195 | 1.862 | 2.296 | 0.8 | 76.2 | 93.9 | 93.9 | 0.00332 | 1.00 | 0.00431 | 1.89 | 0.0152 |
| ***27878-02B*** | 1.4 | 0.02240 | 0.00004 | 4105.1 | 58.0 | 795.7 | 5.0 | 17.8 | 2.0 | 25.2 | 4.8 | 13.3 | 0.9 | 0.37 | 12.0 | 0.225 | 0.043 | 0.182 | 0.336 | 3.5 | 7.5 | 13.8 | 13.8 | 0.00323 | 6.75 | 0.19391 | 1.55 | 0.1911 |
| ***27878-02C*** | 2.0 | 0.02240 | 0.00004 | 3919.8 | 58.4 | 2888.1 | 12.0 | 43.2 | 2.0 | 14.5 | 4.6 | 6.9 | 0.8 | 1.34 | 43.7 | 0.035 | 0.011 | 0.646 | 0.088 | 47.6 | 26.4 | 3.6 | 3.6 | 0.00175 | 12.18 | 0.73726 | 1.55 | 0.1177 |
| ***27878-02D*** | 2.7 | 0.02240 | 0.00004 | 1764.7 | 56.9 | 2475.2 | 8.2 | 35.3 | 1.6 | 0.9 | 4.6 | 2.3 | 0.8 | 1.14 | 37.5 | 0.002 | 0.013 | 0.437 | 0.097 | 61.4 | 17.9 | 4.0 | 4.0 | 0.00129 | 34.43 | 1.40381 | 3.24 | 0.0931 |
| ***27878-02E*** | 3.8 | 0.02240 | 0.00004 | 522.5 | 56.6 | 274.0 | 3.2 | -3.1 | 1.5 | 1.8 | 4.6 | 0.8 | 0.8 | 0.13 | 4.1 | 0.047 | 0.120 | 1.038 | 0.876 | 54.5 | 42.5 | 35.8 | 35.8 | 0.00153 | 98.64 | 0.52466 | 10.89 | 0.1091 |
| OLOR16/SKG-1pB1 - Aliquot #3 | | | | | | | | | | | | | | | | | | | | | | | | | | | | |
| 27878-03A | 1.0 | 0.02240 | 0.00004 | 10811.3 | 58.4 | 100.5 | 2.6 | 6.4 | 1.5 | 1.9 | 4.6 | 33.2 | 1.0 | 0.05 | 0.7 | 0.135 | 0.323 | 8.837 | 2.980 | 8.2 | 361.5 | 121.9 | 121.9 | 0.00307 | 3.00 | 0.00930 | 2.64 | 0.0367 |
| 27878-03B | 1.4 | 0.02240 | 0.00004 | 9415.5 | 59.7 | 1294.2 | 6.5 | 18.8 | 1.6 | 7.9 | 4.6 | 24.2 | 0.9 | 0.60 | 8.8 | 0.043 | 0.025 | 1.697 | 0.213 | 23.3 | 69.4 | 8.7 | 8.7 | 0.00257 | 3.78 | 0.13752 | 0.81 | 0.1319 |
| 27878-03C | 2.0 | 0.02240 | 0.00004 | 13200.9 | 60.2 | 4503.4 | 14.9 | 72.5 | 2.0 | 1.2 | 4.6 | 33.4 | 1.0 | 2.08 | 30.5 | 0.001 | 0.007 | 0.715 | 0.068 | 24.4 | 29.3 | 2.8 | 2.8 | 0.00253 | 3.04 | 0.34133 | 0.56 | 0.1218 |
| ***27878-03D*** | 2.7 | 0.02240 | 0.00004 | 17589.3 | 60.7 | 7279.3 | 18.6 | 125.8 | 3.1 | -3.4 | 4.6 | 49.2 | 1.1 | 3.36 | 49.2 | -0.004 | 0.004 | 0.400 | 0.045 | 16.6 | 16.4 | 1.8 | 1.8 | 0.00279 | 2.21 | 0.41408 | 0.43 | 0.1256 |
| ***27878-03E*** | 3.8 | 0.02240 | 0.00004 | 2242.4 | 57.5 | 1359.8 | 7.0 | 15.8 | 1.8 | -9.0 | 4.6 | 4.3 | 0.8 | 0.63 | 9.2 | -0.048 | 0.024 | 0.693 | 0.184 | 42.0 | 28.3 | 7.5 | 7.5 | 0.00194 | 18.88 | 0.60680 | 2.62 | 0.1332 |
| ***27878-03F*** | 5.4 | 0.02240 | 0.00004 | 448.8 | 56.8 | 247.4 | 3.1 | 2.7 | 1.6 | -1.3 | 4.5 | 0.5 | 0.8 | 0.11 | 1.7 | -0.039 | 0.130 | 1.186 | 0.963 | 65.4 | 48.5 | 39.4 | 39.4 | 0.00116 | 149.74 | 0.55164 | 12.72 | 0.0842 |
| OLOR16/SKG-1pB1 - Aliquot #4 | | | | | | | | | | | | | | | | | | | | | | | | | | | | |
| ***27878-04A*** | 1.0 | 0.02240 | 0.00004 | 1728.3 | 57.9 | 35.9 | 2.2 | -6.4 | 1.7 | -3.4 | 4.5 | 5.4 | 0.9 | 0.02 | 0.7 | -0.676 | 0.899 | 3.063 | 7.243 | 6.4 | 125.3 | 296.3 | 296.3 | 0.00314 | 16.05 | 0.02081 | 6.96 | 0.1005 |
| ***27878-04B*** | 1.4 | 0.02240 | 0.00004 | 3651.1 | 59.4 | 324.3 | 3.5 | 14.0 | 1.7 | 1.2 | 4.4 | 12.1 | 0.9 | 0.15 | 6.6 | 0.026 | 0.096 | 0.082 | 0.869 | 0.7 | 3.4 | 35.6 | 35.6 | 0.00333 | 7.78 | 0.08887 | 1.95 | 0.1748 |
| ***27878-04C*** | 2.0 | 0.02240 | 0.00004 | 7710.4 | 60.6 | 2532.3 | 9.1 | 46.2 | 1.7 | 12.2 | 4.4 | 22.0 | 0.9 | 1.17 | 51.2 | 0.034 | 0.012 | 0.449 | 0.109 | 14.8 | 18.4 | 4.5 | 4.5 | 0.00286 | 4.18 | 0.32861 | 0.86 | 0.1711 |
| ***27878-04D*** | 2.7 | 0.02240 | 0.00004 | 1258.4 | 58.8 | 1825.0 | 9.8 | 24.0 | 1.9 | -2.6 | 4.3 | 2.4 | 0.8 | 0.85 | 36.9 | -0.010 | 0.017 | 0.300 | 0.140 | 43.5 | 12.3 | 5.7 | 5.7 | 0.00189 | 35.35 | 1.45150 | 4.71 | 0.1314 |
| ***27878-04E*** | 3.8 | 0.02240 | 0.00004 | 155.5 | 58.5 | 226.5 | 3.2 | 1.3 | 1.3 | 1.9 | 4.3 | 1.4 | 0.8 | 0.10 | 4.6 | 0.059 | 0.136 | -1.112 | 1.088 | -162.1 | -45.5 | 44.5 | 44.5 | 0.00878 | 69.79 | 1.45814 | 37.67 | 0.5390 |
| OLOR16/SKG-1pB1 - Aliquot #5 | | | | | | | | | | | | | | | | | | | | | | | | | | | | |
| 27878-05A | 1.0 | 0.02240 | 0.00004 | 43842.4 | 69.1 | 53.9 | 2.5 | 39.8 | 2.1 | -0.5 | 4.3 | 150.3 | 1.6 | 0.02 | 0.3 | -0.070 | 0.566 | -18.877 | 8.817 | -2.3 | -772.4 | 360.8 | 360.8 | 0.00343 | 1.06 | 0.00123 | 4.55 | 0.0052 |
| 27878-05B | 1.4 | 0.02240 | 0.00004 | 396189.6 | 159.4 | 361.0 | 4.2 | 391.9 | 7.5 | 1.9 | 4.3 | 1349.6 | 6.1 | 0.17 | 2.1 | 0.037 | 0.085 | -18.667 | 5.050 | -1.7 | -763.8 | 206.7 | 206.7 | 0.00341 | 0.45 | 0.00091 | 1.17 | 0.0031 |
| ***27878-05C*** | 2.0 | 0.02240 | 0.00004 | 17122.6 | 64.7 | 2437.3 | 9.3 | 91.1 | 2.4 | 12.9 | 4.3 | 54.7 | 1.2 | 1.13 | 14.0 | 0.037 | 0.013 | 0.327 | 0.147 | 4.7 | 13.4 | 6.0 | 6.0 | 0.00319 | 2.19 | 0.14241 | 0.54 | 0.1214 |
| ***27878-05D*** | 2.7 | 0.02240 | 0.00004 | 45360.1 | 68.9 | 5124.5 | 12.3 | 131.5 | 3.0 | 16.1 | 4.3 | 142.6 | 1.6 | 2.37 | 29.4 | 0.022 | 0.006 | 0.547 | 0.096 | 6.2 | 22.4 | 3.9 | 3.9 | 0.00314 | 1.16 | 0.11303 | 0.28 | 0.0703 |
| ***27878-05E*** | 3.8 | 0.02240 | 0.00004 | 10030.5 | 62.5 | 6031.2 | 14.9 | 102.8 | 2.6 | 20.6 | 4.4 | 25.0 | 1.0 | 2.79 | 34.6 | 0.024 | 0.005 | 0.427 | 0.049 | 25.7 | 17.5 | 2.0 | 2.0 | 0.00249 | 3.91 | 0.60165 | 0.67 | 0.1479 |
| ***27878-05F*** | 5.4 | 0.02240 | 0.00004 | 7444.7 | 62.0 | 3153.8 | 11.5 | 53.9 | 1.8 | 7.1 | 4.3 | 21.6 | 0.9 | 1.46 | 18.1 | 0.016 | 0.010 | 0.312 | 0.092 | 13.2 | 12.7 | 3.8 | 3.8 | 0.00291 | 4.47 | 0.42386 | 0.91 | 0.1708 |
| ***27878-05G*** | 7.5 | 0.02240 | 0.00004 | 1366.0 | 59.7 | 245.0 | 3.2 | 2.8 | 1.4 | -1.7 | 4.3 | 3.8 | 0.8 | 0.11 | 1.4 | -0.051 | 0.126 | 0.973 | 1.049 | 17.5 | 39.8 | 42.9 | 42.9 | 0.00276 | 22.61 | 0.17948 | 4.57 | 0.1853 |
| OLOR16/SKG-1pB1 - Aliquot #6 | | | | | | | | | | | | | | | | | | | | | | | | | | | | |
| ***27878-06A*** | 1.0 | 0.02240 | 0.00004 | 6746.7 | 59.5 | 98.0 | 2.3 | 1.6 | 1.6 | -1.4 | 4.3 | 21.5 | 0.9 | 0.05 | 2.0 | -0.104 | 0.312 | 3.241 | 2.940 | 4.7 | 132.6 | 120.3 | 120.3 | 0.00319 | 4.47 | 0.01453 | 2.49 | 0.0700 |
| ***27878-06B*** | 1.4 | 0.02240 | 0.00004 | 3199.3 | 58.8 | 450.0 | 4.3 | -1.0 | 1.7 | 0.8 | 4.3 | 9.5 | 0.8 | 0.21 | 9.3 | 0.012 | 0.069 | 0.774 | 0.557 | 10.9 | 31.7 | 22.8 | 22.8 | 0.00298 | 8.75 | 0.14072 | 2.07 | 0.1864 |
| ***27878-06C*** | 2.0 | 0.02240 | 0.00004 | 3671.2 | 59.1 | 1092.4 | 6.5 | 10.2 | 1.4 | -7.7 | 4.3 | 10.0 | 0.8 | 0.51 | 22.5 | -0.051 | 0.028 | 0.621 | 0.237 | 18.5 | 25.4 | 9.7 | 9.7 | 0.00273 | 8.57 | 0.29772 | 1.72 | 0.1764 |
| ***27878-06D*** | 2.7 | 0.02240 | 0.00004 | 1077.8 | 59.9 | 610.3 | 4.8 | 3.2 | 1.5 | 1.3 | 4.3 | 2.2 | 0.8 | 0.28 | 12.6 | 0.015 | 0.050 | 0.705 | 0.408 | 39.9 | 28.8 | 16.7 | 16.7 | 0.00201 | 37.76 | 0.56660 | 5.62 | 0.1458 |
| ***27878-06E*** | 3.8 | 0.02240 | 0.00004 | 933.8 | 59.6 | 1010.4 | 5.5 | 8.7 | 1.3 | 6.0 | 4.4 | 2.3 | 0.8 | 0.47 | 20.8 | 0.042 | 0.031 | 0.252 | 0.235 | 27.3 | 10.3 | 9.6 | 9.6 | 0.00243 | 34.48 | 1.08283 | 6.41 | 0.1846 |
| ***27878-06F*** | 5.4 | 0.02240 | 0.00004 | 928.7 | 59.9 | 1430.8 | 5.2 | 13.9 | 1.5 | 14.5 | 4.4 | 2.0 | 0.8 | 0.66 | 29.5 | 0.072 | 0.022 | 0.240 | 0.169 | 37.1 | 9.8 | 6.9 | 6.9 | 0.00211 | 40.68 | 1.54191 | 6.47 | 0.1584 |
| ***27878-06G*** | 7.5 | 0.02240 | 0.00004 | 119.9 | 56.3 | 161.0 | 2.9 | -5.1 | 1.4 | -7.0 | 4.3 | 1.1 | 0.8 | 0.07 | 3.3 | -0.313 | 0.193 | -1.287 | 1.513 | -172.9 | -52.7 | 61.9 | 61.9 | 0.00914 | 86.36 | 1.34325 | 47.01 | 0.5435 |

| | | | | | | | | | | | | | | | | | | | | | | | | | | | | |
|---|---|---|---|---|---|---|---|---|---|---|---|---|---|---|---|---|---|---|---|---|---|---|---|---|---|---|---|---|
| OLOR16/SKG-1pB1 - Aliquot #7 | | | | | | | | | | | | | | | | | | | | | | | | | | | | |
| ***27878-07A*** | 1.0 | 0.02240 | 0.00004 | 227216.1 | 114.0 | 222.2 | 2.8 | 230.3 | 4.8 | 9.4 | 4.5 | 764.4 | 4.2 | 0.10 | 3.0 | 0.302 | 0.144 | -4.469 | 5.692 | -0.4 | -182.8 | 232.9 | 232.9 | 0.00336 | 0.55 | 0.00098 | 1.25 | 0.0036 |
| ***27878-07B*** | 1.4 | 0.02240 | 0.00004 | 12131.0 | 62.7 | 1576.9 | 8.0 | 61.4 | 1.9 | 17.8 | 4.6 | 40.8 | 1.0 | 0.73 | 21.4 | 0.080 | 0.021 | -0.027 | 0.199 | -0.4 | -1.1 | 8.1 | 8.1 | 0.00336 | 2.57 | 0.13005 | 0.72 | 0.1436 |
| ***27878-07C*** | 2.0 | 0.02240 | 0.00004 | 17370.3 | 62.2 | 4724.5 | 11.9 | 92.5 | 2.3 | 18.8 | 4.5 | 52.4 | 1.0 | 2.19 | 64.0 | 0.028 | 0.007 | 0.364 | 0.063 | 9.9 | 14.9 | 2.6 | 2.6 | 0.00302 | 1.89 | 0.27213 | 0.44 | 0.1550 |
| ***27878-07D*** | 2.7 | 0.02240 | 0.00004 | 2309.6 | 59.8 | 716.7 | 6.3 | 12.6 | 1.5 | -8.4 | 4.5 | 8.4 | 0.8 | 0.33 | 9.7 | -0.084 | 0.045 | -0.267 | 0.331 | -8.3 | -10.9 | 13.6 | 13.6 | 0.00363 | 9.55 | 0.31048 | 2.73 | 0.2570 |
| ***27878-07E*** | 3.8 | 0.02240 | 0.00004 | 225.1 | 59.5 | 137.4 | 2.7 | 1.4 | 1.9 | -17.0 | 4.5 | 1.0 | 0.8 | 0.06 | 1.9 | -0.888 | 0.234 | -0.634 | 1.687 | -38.7 | -25.9 | 69.0 | 69.0 | 0.00465 | 76.53 | 0.61106 | 26.50 | 0.3444 |
| OLOR16/SKG-1pB1 - Aliquot #8 | | | | | | | | | | | | | | | | | | | | | | | | | | | | |
| 27878-08A | 1.0 | 0.02240 | 0.00004 | 12190.3 | 59.7 | 103.3 | 2.7 | 10.3 | 1.6 | 2.9 | 4.6 | 38.0 | 1.0 | 0.05 | 1.9 | 0.199 | 0.318 | 8.325 | 2.951 | 7.1 | 340.5 | 120.7 | 120.7 | 0.00311 | 2.68 | 0.00848 | 2.64 | 0.0340 |
| ***27878-08B*** | 1.4 | 0.02240 | 0.00004 | 10117.7 | 61.1 | 851.4 | 4.4 | 19.0 | 1.7 | 2.1 | 4.5 | 33.5 | 1.0 | 0.40 | 16.0 | 0.017 | 0.038 | 0.153 | 0.360 | 1.3 | 6.3 | 14.7 | 14.7 | 0.00331 | 3.06 | 0.08419 | 0.80 | 0.1493 |
| ***27878-08C*** | 2.0 | 0.02240 | 0.00004 | 29744.5 | 64.3 | 4084.8 | 10.1 | 78.8 | 2.4 | 0.2 | 4.5 | 94.8 | 1.3 | 1.90 | 76.9 | 0.000 | 0.008 | 0.349 | 0.095 | 4.8 | 14.3 | 3.9 | 3.9 | 0.00319 | 1.38 | 0.13740 | 0.33 | 0.1036 |
| ***27878-08D*** | 2.7 | 0.02240 | 0.00004 | 1072.3 | 56.8 | 271.6 | 3.8 | 1.1 | 1.6 | 15.0 | 4.5 | 4.5 | 0.8 | 0.13 | 5.1 | 0.398 | 0.118 | -0.985 | 0.922 | -25.0 | -40.3 | 37.7 | 37.7 | 0.00419 | 18.95 | 0.25335 | 5.48 | 0.2699 |
| OLOR16/SKG-1pB1 - Aliquot #9 | | | | | | | | | | | | | | | | | | | | | | | | | | | | |
| ***27878-09A*** | 1.0 | 0.02240 | 0.00004 | 7931.3 | 58.6 | 121.0 | 2.7 | 3.5 | 1.6 | -5.2 | 4.5 | 26.7 | 0.9 | 0.06 | 1.8 | -0.308 | 0.269 | -0.447 | 2.300 | -0.7 | -18.3 | 94.1 | 94.1 | 0.00337 | 3.49 | 0.01526 | 2.38 | 0.0658 |
| ***27878-09B*** | 1.4 | 0.02240 | 0.00004 | 12713.0 | 59.9 | 887.8 | 5.0 | 20.6 | 1.4 | 3.7 | 4.6 | 41.5 | 1.0 | 0.41 | 13.5 | 0.030 | 0.037 | 0.372 | 0.358 | 2.6 | 15.2 | 14.7 | 14.7 | 0.00326 | 2.57 | 0.06987 | 0.74 | 0.1173 |
| ***27878-09C*** | 2.0 | 0.02240 | 0.00004 | 21860.8 | 62.5 | 3913.7 | 13.6 | 69.7 | 1.7 | 7.6 | 4.6 | 66.8 | 1.1 | 1.82 | 59.5 | 0.014 | 0.009 | 0.489 | 0.085 | 8.8 | 20.0 | 3.5 | 3.5 | 0.00306 | 1.66 | 0.17912 | 0.45 | 0.1090 |
| ***27878-09D*** | 2.7 | 0.02240 | 0.00004 | 2689.7 | 58.3 | 1387.5 | 6.4 | 21.9 | 1.6 | 28.5 | 4.7 | 5.7 | 0.8 | 0.64 | 21.1 | 0.147 | 0.024 | 0.723 | 0.177 | 37.3 | 29.6 | 7.3 | 7.3 | 0.00210 | 14.35 | 0.51613 | 2.22 | 0.1478 |
| ***27878-09E*** | 3.8 | 0.02240 | 0.00004 | 428.7 | 57.6 | 262.5 | 3.1 | 3.1 | 1.6 | 9.8 | 4.6 | 1.7 | 0.8 | 0.12 | 4.0 | 0.269 | 0.125 | -0.254 | 0.909 | -15.6 | -10.4 | 37.2 | 37.2 | 0.00387 | 48.66 | 0.61249 | 13.49 | 0.2752 |
| OLOR16/SKG-1pB1 - Aliquot #10 | | | | | | | | | | | | | | | | | | | | | | | | | | | | |
| ***27878-10A*** | 1.0 | 0.02240 | 0.00004 | 26005.8 | 60.5 | 94.3 | 2.3 | 20.1 | 1.7 | 3.8 | 4.8 | 87.3 | 1.3 | 0.04 | 1.3 | 0.292 | 0.370 | -0.714 | 4.152 | -0.3 | -29.2 | 169.8 | 169.8 | 0.00336 | 1.50 | 0.00363 | 2.47 | 0.0146 |
| ***27878-10B*** | 1.4 | 0.02240 | 0.00004 | 4073.2 | 57.7 | 551.6 | 5.5 | 7.7 | 1.4 | 4.8 | 4.7 | 12.6 | 0.9 | 0.26 | 7.8 | 0.063 | 0.061 | 0.545 | 0.488 | 7.4 | 22.3 | 20.0 | 20.0 | 0.00310 | 7.12 | 0.13548 | 1.73 | 0.1626 |
| ***27878-10C*** | 2.0 | 0.02240 | 0.00004 | 12232.8 | 59.5 | 1948.4 | 8.1 | 32.7 | 1.9 | -1.8 | 4.6 | 36.6 | 1.0 | 0.91 | 27.4 | -0.007 | 0.017 | 0.669 | 0.150 | 10.7 | 27.4 | 6.2 | 6.2 | 0.00299 | 2.67 | 0.15936 | 0.64 | 0.1383 |
| ***27878-10D*** | 2.7 | 0.02240 | 0.00004 | 3511.5 | 56.7 | 3736.9 | 10.6 | 53.2 | 1.8 | -9.2 | 4.7 | 7.1 | 0.8 | 1.74 | 52.6 | -0.018 | 0.009 | 0.369 | 0.065 | 39.2 | 15.1 | 2.7 | 2.7 | 0.00203 | 11.25 | 1.06496 | 1.64 | 0.1413 |
| ***27878-10E*** | 3.8 | 0.02240 | 0.00004 | 823.7 | 57.3 | 657.0 | 4.3 | 19.2 | 1.5 | 13.9 | 4.8 | 2.5 | 0.7 | 0.31 | 9.3 | 0.152 | 0.052 | 0.111 | 0.345 | 8.9 | 4.6 | 14.1 | 14.1 | 0.00305 | 30.10 | 0.79812 | 6.99 | 0.2301 |
| ***27878-10F*** | 5.4 | 0.02240 | 0.00004 | 253.0 | 57.3 | 112.8 | 2.6 | 1.8 | 1.4 | -16.5 | 4.6 | -1.2 | 0.8 | 0.05 | 1.6 | -1.054 | 0.295 | 5.324 | 2.092 | 237.6 | 217.8 | 85.6 | 85.6 | - 0.00461 | 69.50 | 0.44634 | 22.77 | 0.3242 |
| OLOR16/SKG-1pB1 - Aliquot #11 | | | | | | | | | | | | | | | | | | | | | | | | | | | | |
| 27878-11A | 1.0 | 0.02240 | 0.00004 | 5436.5 | 56.2 | 24.1 | 1.8 | 4.2 | 1.5 | -2.2 | 4.6 | 19.2 | 0.9 | 0.01 | 0.9 | -0.647 | 1.381 | -11.923 | 11.259 | -5.3 | -487.8 | 460.7 | 460.7 | 0.00353 | 4.73 | 0.00443 | 7.66 | 0.0294 |
| 27878-11B | 1.4 | 0.02240 | 0.00004 | 4532.1 | 55.8 | 236.0 | 4.0 | 3.1 | 1.3 | 7.0 | 4.5 | 10.1 | 0.8 | 0.11 | 9.2 | 0.213 | 0.139 | 6.467 | 1.100 | 33.7 | 264.5 | 45.0 | 45.0 | 0.00222 | 8.49 | 0.05210 | 2.11 | 0.0848 |
| 27878-11C | 2.0 | 0.02240 | 0.00004 | 7209.0 | 56.6 | 1101.1 | 5.8 | 21.2 | 1.5 | 13.4 | 4.5 | 12.5 | 0.9 | 0.51 | 42.9 | 0.087 | 0.029 | 3.172 | 0.249 | 48.5 | 129.8 | 10.2 | 10.2 | 0.00173 | 7.26 | 0.15282 | 0.94 | 0.0901 |
| 27878-11D | 2.7 | 0.02240 | 0.00004 | 3368.6 | 55.8 | 670.6 | 5.3 | 11.5 | 1.5 | 30.8 | 4.5 | 11.0 | 0.9 | 0.31 | 26.1 | 0.332 | 0.049 | 0.133 | 0.409 | 2.6 | 5.4 | 16.7 | 16.7 | 0.00326 | 8.35 | 0.19915 | 1.83 | 0.1792 |
| 27878-11E | 3.8 | 0.02240 | 0.00004 | 2014.4 | 55.4 | 358.0 | 4.4 | 5.4 | 1.6 | 5.4 | 4.4 | 6.0 | 0.8 | 0.17 | 14.0 | 0.108 | 0.089 | 0.617 | 0.665 | 11.0 | 25.2 | 27.2 | 27.2 | 0.00298 | 13.20 | 0.17782 | 3.01 | 0.1903 |
| 27878-11F | 5.4 | 0.02240 | 0.00004 | 3571.1 | 55.8 | 175.4 | 3.0 | 3.3 | 1.4 | 6.5 | 4.4 | 13.2 | 0.9 | 0.08 | 6.8 | 0.267 | 0.181 | -2.048 | 1.516 | -10.1 | -83.8 | 62.0 | 62.0 | 0.00369 | 6.80 | 0.04913 | 2.32 | 0.1551 |
| OLOR16/SKG-2p1 | | | | | | | | | | | | | | | | | | | | | | | | | | | | |
| 27877-01A | 1.0 | 0.02240 | 0.00004 | 3127.9 | 63.1 | 181.6 | 3.4 | 13.7 | 1.5 | -6.4 | 4.5 | 12.6 | 0.8 | 0.08 | 1.6 | -0.243 | 0.172 | -3.487 | 1.370 | -20.3 | -142.7 | 56.0 | 56.0 | 0.00403 | 6.70 | 0.05808 | 2.74 | 0.2215 |
| ***27877-01B*** | 1.4 | 0.02240 | 0.00004 | 3309.6 | 62.3 | 931.7 | 6.5 | 22.0 | 2.2 | 5.6 | 4.8 | 11.8 | 0.9 | 0.43 | 8.3 | 0.041 | 0.035 | -0.225 | 0.293 | -6.3 | -9.2 | 12.0 | 12.0 | 0.00356 | 7.78 | 0.28165 | 2.01 | 0.2268 |
| ***27877-01C*** | 2.0 | 0.02240 | 0.00004 | 3162.6 | 61.0 | 2767.3 | 9.0 | 47.5 | 1.6 | 6.2 | 4.7 | 7.9 | 0.8 | 1.27 | 24.5 | 0.015 | 0.012 | 0.290 | 0.084 | 25.4 | 11.9 | 3.5 | 3.5 | 0.00250 | 9.76 | 0.87561 | 1.96 | 0.1947 |
| ***27877-01D*** | 2.7 | 0.02240 | 0.00004 | 2890.1 | 61.0 | 6140.8 | 14.9 | 100.5 | 2.6 | 9.1 | 4.6 | 4.9 | 0.8 | 2.81 | 54.5 | 0.010 | 0.005 | 0.232 | 0.039 | 49.4 | 9.5 | 1.6 | 1.6 | 0.00170 | 16.00 | 2.12687 | 2.12 | 0.1311 |
| ***27877-01E*** | 3.8 | 0.02240 | 0.00004 | 554.0 | 60.3 | 1073.8 | 6.3 | 17.3 | 1.8 | -4.1 | 4.6 | 2.1 | 0.8 | 0.49 | 9.5 | -0.027 | 0.030 | -0.077 | 0.236 | -15.0 | -3.2 | 9.6 | 9.6 | 0.00385 | 40.11 | 1.94001 | 10.91 | 0.2711 |
| ***27877-01F*** | 5.4 | 0.02240 | 0.00004 | 218.8 | 57.8 | 180.4 | 2.8 | 3.5 | 1.6 | -6.6 | 4.5 | 1.2 | 0.8 | 0.08 | 1.6 | -0.253 | 0.174 | -0.765 | 1.297 | -63.1 | -31.3 | 53.1 | 53.1 | 0.00546 | 68.85 | 0.82487 | 26.47 | 0.3833 |
| OLOR16/SKG-2p1 - Aliquot #2 | | | | | | | | | | | | | | | | | | | | | | | | | | | | |
| ***27877-02A*** | 1.0 | 0.02240 | 0.00004 | 8120.4 | 59.3 | 299.1 | 3.2 | 9.3 | 1.7 | 2.8 | 4.5 | 27.1 | 0.9 | 0.14 | 4.3 | 0.065 | 0.105 | 0.111 | 0.914 | 0.4 | 4.5 | 37.4 | 37.4 | 0.00334 | 3.38 | 0.03685 | 1.29 | 0.1222 |
| ***27877-02B*** | 1.4 | 0.02240 | 0.00004 | 11464.3 | 60.6 | 1987.6 | 10.4 | 37.5 | 1.9 | 12.0 | 4.6 | 37.0 | 0.9 | 0.91 | 28.4 | 0.042 | 0.016 | 0.209 | 0.141 | 3.6 | 8.5 | 5.8 | 5.8 | 0.00323 | 2.54 | 0.17346 | 0.74 | 0.1480 |
| ***27877-02C*** | 2.0 | 0.02240 | 0.00004 | 10560.2 | 59.6 | 3754.9 | 9.9 | 61.0 | 2.3 | 8.7 | 4.6 | 30.5 | 1.0 | 1.72 | 53.6 | 0.016 | 0.008 | 0.390 | 0.078 | 13.9 | 16.0 | 3.2 | 3.2 | 0.00288 | 3.19 | 0.35576 | 0.62 | 0.1601 |
| ***27877-02D*** | 2.7 | 0.02240 | 0.00004 | 738.6 | 57.4 | 778.5 | 5.5 | 9.4 | 1.6 | 5.7 | 4.5 | 1.1 | 0.8 | 0.36 | 11.1 | 0.050 | 0.040 | 0.516 | 0.314 | 54.4 | 21.1 | 12.9 | 12.9 | 0.00153 | 71.18 | 1.05472 | 7.81 | 0.1088 |
| ***27877-02E*** | 3.8 | 0.02240 | 0.00004 | 152.5 | 57.7 | 186.1 | 3.3 | -4.6 | 1.2 | 13.1 | 4.5 | -1.2 | 0.8 | 0.09 | 2.7 | 0.490 | 0.169 | 2.741 | 1.244 | 334.5 | 112.1 | 50.9 | 50.9 | - 0.00785 | 73.19 | 1.22061 | 37.90 | 0.5167 |
| OLOR16/SKG-2p1 - Aliquot #3 | | | | | | | | | | | | | | | | | | | | | | | | | | | | |
| ***27877-03A*** | 1.0 | 0.02240 | 0.00004 | 6084.8 | 59.5 | 340.8 | 3.5 | 7.8 | 1.4 | -2.8 | 4.5 | 20.7 | 0.9 | 0.16 | 5.2 | -0.058 | 0.091 | -0.295 | 0.823 | -1.7 | -12.1 | 33.7 | 33.7 | 0.00340 | 4.54 | 0.05603 | 1.41 | 0.1491 |
| ***27877-03B*** | 1.4 | 0.02240 | 0.00004 | 9381.5 | 59.5 | 2414.6 | 9.8 | 41.8 | 1.8 | 17.4 | 4.6 | 28.9 | 1.0 | 1.11 | 37.0 | 0.050 | 0.013 | 0.309 | 0.124 | 8.0 | 12.7 | 5.1 | 5.1 | 0.00308 | 3.45 | 0.25751 | 0.75 | 0.1548 |
| ***27877-03C*** | 2.0 | 0.02240 | 0.00004 | 6271.7 | 58.6 | 3148.5 | 9.8 | 49.4 | 1.9 | -5.3 | 4.4 | 19.3 | 0.8 | 1.44 | 48.3 | -0.012 | 0.010 | 0.164 | 0.081 | 8.2 | 6.7 | 3.3 | 3.3 | 0.00307 | 4.40 | 0.50232 | 0.99 | 0.2015 |
| ***27877-03D*** | 2.7 | 0.02240 | 0.00004 | 396.8 | 57.5 | 533.1 | 4.4 | 6.2 | 1.7 | 17.5 | 4.5 | 0.5 | 0.8 | 0.24 | 8.2 | 0.228 | 0.059 | 0.485 | 0.444 | 65.2 | 19.8 | 18.2 | 18.2 | 0.00117 | 166.96 | 1.34449 | 14.51 | 0.0866 |
| ***27877-03E*** | 3.8 | 0.02240 | 0.00004 | 58.3 | 58.2 | 81.5 | 2.5 | 11.9 | 1.8 | 1.9 | 4.4 | -0.6 | 0.8 | 0.04 | 1.3 | 0.163 | 0.377 | 2.909 | 2.982 | 407.5 | 119.0 | 122.0 | 122.0 | - 0.01030 | 165.44 | 1.40072 | 99.99 | 0.6039 |

| | | | | | | | | | | | | | | | | | | | | | | | | | | | | |
|---|---|---|---|---|---|---|---|---|---|---|---|---|---|---|---|---|---|---|---|---|---|---|---|---|---|---|---|---|
| OLOR16/SKG-2p1 - Aliquot #4 | | | | | | | | | | | | | | | | | | | | | | | | | | | | |
| ***27877-04A*** | 1.0 | 0.02240 | 0.00004 | 3310.5 | 58.9 | 138.0 | 2.4 | 9.0 | 1.5 | -5.8 | 4.4 | 11.1 | 0.9 | 0.06 | 2.1 | -0.290 | 0.223 | -0.002 | 2.051 | 0.0 | -0.1 | 83.9 | 83.9 | 0.00335 | 8.55 | 0.04171 | 2.46 | 0.1502 |
| ***27877-04B*** | 1.4 | 0.02240 | 0.00004 | 659.2 | 57.1 | 868.6 | 6.0 | 11.8 | 1.7 | -9.5 | 4.4 | 0.3 | 0.8 | 0.40 | 13.3 | -0.076 | 0.035 | 0.642 | 0.278 | 84.6 | 26.2 | 11.4 | 11.4 | 0.00052 | 231.21 | 1.31872 | 8.69 | 0.0373 |
| ***27877-04C*** | 2.0 | 0.02240 | 0.00004 | 964.2 | 57.1 | 3209.3 | 11.4 | 49.3 | 2.2 | 10.1 | 4.4 | -0.5 | 0.8 | 1.47 | 49.3 | 0.022 | 0.010 | 0.348 | 0.078 | 116.1 | 14.3 | 3.2 | 3.2 | - 0.00054 | 156.96 | 3.33261 | 5.93 | 0.0377 |
| ***27877-04D*** | 2.7 | 0.02240 | 0.00004 | 994.4 | 57.1 | 2166.1 | 8.7 | 31.3 | 2.0 | -7.0 | 4.3 | 0.8 | 0.8 | 0.99 | 33.3 | -0.023 | 0.014 | 0.348 | 0.120 | 76.0 | 14.3 | 4.9 | 4.9 | 0.00081 | 106.04 | 2.18048 | 5.76 | 0.0540 |
| ***27877-04E*** | 3.8 | 0.02240 | 0.00004 | 57.3 | 57.8 | 129.1 | 3.3 | -2.7 | 1.3 | -4.5 | 4.3 | -0.2 | 0.8 | 0.06 | 2.0 | -0.243 | 0.235 | 0.865 | 1.912 | 195.3 | 35.4 | 78.2 | 78.2 | - 0.00319 | 451.64 | 2.25670 | 100.95 | 0.2234 |
| | | | | | | | | | | | | | | | | | | | | | | | | | | | | |
| OLOR16/SKG-2p1 - Aliquot #5 | | | | | | | | | | | | | | | | | | | | | | | | | | | | |
| ***27877-05A*** | 1.0 | 0.02240 | 0.00004 | 2588.1 | 58.4 | 32.5 | 1.9 | -0.5 | 1.4 | 2.3 | 4.3 | 9.1 | 0.9 | 0.01 | 0.6 | 0.494 | 0.926 | -3.703 | 8.087 | -4.6 | -151.5 | 330.9 | 330.9 | 0.00351 | 9.72 | 0.01255 | 6.14 | 0.0854 |
| ***27877-05B*** | 1.4 | 0.02240 | 0.00004 | 9063.1 | 60.4 | 282.7 | 3.3 | 10.6 | 1.6 | -4.9 | 4.4 | 30.5 | 1.0 | 0.13 | 4.9 | -0.122 | 0.109 | -0.129 | 1.036 | -0.4 | -5.3 | 42.4 | 42.4 | 0.00336 | 3.22 | 0.03121 | 1.33 | 0.1035 |
| ***27877-05C*** | 2.0 | 0.02240 | 0.00004 | 3817.2 | 75.8 | 868.6 | 5.4 | 17.7 | 1.6 | -2.0 | 4.3 | 13.1 | 0.9 | 0.40 | 15.0 | -0.016 | 0.035 | -0.094 | 0.318 | -2.1 | -3.9 | 13.0 | 13.0 | 0.00342 | 7.10 | 0.22766 | 2.08 | 0.2667 |
| ***27877-05D*** | 2.7 | 0.02240 | 0.00004 | 4489.0 | 75.8 | 4401.1 | 13.6 | 67.7 | 2.2 | -3.1 | 4.3 | 12.2 | 0.9 | 2.02 | 76.3 | -0.005 | 0.007 | 0.190 | 0.062 | 18.6 | 7.8 | 2.5 | 2.5 | 0.00273 | 7.40 | 0.98111 | 1.72 | 0.2244 |
| ***27877-05E*** | 3.8 | 0.02240 | 0.00004 | -520.5 | 75.1 | 186.8 | 2.8 | 1.4 | 1.4 | -6.9 | 4.3 | -1.1 | 0.8 | 0.09 | 3.2 | -0.259 | 0.161 | -1.055 | 1.273 | 37.9 | -43.2 | 52.1 | 52.1 | 0.00208 | 71.28 | - 0.35899 | 14.50 | 0.2012 |
| | | | | | | | | | | | | | | | | | | | | | | | | | | | | |
| OLOR16/SKG-2p1 - Aliquot #6 | | | | | | | | | | | | | | | | | | | | | | | | | | | | |
| ***27877-06A*** | 1.0 | 0.02240 | 0.00004 | 28059.5 | 62.3 | 200.6 | 3.0 | 24.6 | 1.9 | -7.8 | 4.4 | 93.2 | 1.2 | 0.09 | 2.8 | -0.273 | 0.152 | 1.091 | 1.800 | 0.8 | 44.6 | 73.6 | 73.6 | 0.00332 | 1.30 | 0.00715 | 1.49 | 0.0255 |
| ***27877-06B*** | 1.4 | 0.02240 | 0.00004 | 878.1 | 57.4 | 1019.6 | 5.5 | 12.9 | 1.7 | -13.2 | 4.4 | 1.1 | 0.8 | 0.47 | 14.4 | -0.091 | 0.030 | 0.542 | 0.239 | 62.9 | 22.2 | 9.8 | 9.8 | 0.00124 | 73.11 | 1.16198 | 6.56 | 0.0891 |
| ***27877-06C*** | 2.0 | 0.02240 | 0.00004 | 1372.4 | 57.4 | 4552.3 | 11.8 | 64.6 | 2.3 | -5.5 | 4.4 | 0.9 | 0.7 | 2.09 | 64.2 | -0.009 | 0.007 | 0.239 | 0.049 | 79.5 | 9.8 | 2.0 | 2.0 | 0.00069 | 77.04 | 3.32129 | 4.19 | 0.0542 |
| ***27877-06D*** | 2.7 | 0.02240 | 0.00004 | 444.3 | 55.3 | 1251.4 | 6.6 | 13.1 | 1.7 | -10.6 | 4.4 | 0.4 | 0.8 | 0.57 | 17.6 | -0.060 | 0.025 | 0.248 | 0.194 | 70.0 | 10.2 | 7.9 | 7.9 | 0.00101 | 177.70 | 2.81972 | 12.46 | 0.0700 |
| ***27877-06E*** | 3.8 | 0.02240 | 0.00004 | 85.5 | 55.3 | 71.1 | 2.5 | -2.8 | 1.5 | -12.2 | 4.5 | 0.5 | 0.8 | 0.03 | 1.0 | -1.201 | 0.443 | -0.867 | 3.351 | -72.3 | -35.5 | 137.1 | 137.1 | 0.00577 | 170.44 | 0.83327 | 64.76 | 0.3789 |
| | | | | | | | | | | | | | | | | | | | | | | | | | | | | |
| OLOR16/SKG-2p1 - Aliquot #7 | | | | | | | | | | | | | | | | | | | | | | | | | | | | |
| ***27877-07A*** | 1.0 | 0.02240 | 0.00004 | 4434.3 | 57.5 | 102.4 | 2.6 | -0.2 | 1.7 | -9.6 | 4.5 | 14.0 | 0.9 | 0.05 | 0.5 | -0.658 | 0.307 | 2.513 | 2.706 | 5.8 | 102.8 | 110.7 | 110.7 | 0.00315 | 6.62 | 0.02311 | 2.87 | 0.0885 |
| ***27877-07B*** | 1.4 | 0.02240 | 0.00004 | 3643.3 | 59.7 | 931.8 | 5.5 | 10.3 | 1.5 | -15.6 | 4.6 | 11.5 | 0.9 | 0.43 | 5.0 | -0.118 | 0.034 | 0.227 | 0.288 | 5.8 | 9.3 | 11.8 | 11.8 | 0.00315 | 7.79 | 0.25590 | 1.74 | 0.1978 |
| ***27877-07C*** | 2.0 | 0.02240 | 0.00004 | 9775.9 | 60.7 | 6407.8 | 18.6 | 114.8 | 2.2 | 14.1 | 4.8 | 25.2 | 1.0 | 2.95 | 34.3 | 0.015 | 0.005 | 0.352 | 0.045 | 23.1 | 14.4 | 1.9 | 1.9 | 0.00258 | 3.83 | 0.65587 | 0.69 | 0.1471 |
| ***27877-07D*** | 2.7 | 0.02240 | 0.00004 | 3075.4 | 59.1 | 8658.8 | 21.1 | 142.2 | 2.8 | -3.7 | 4.9 | 2.0 | 0.8 | 3.98 | 46.4 | -0.003 | 0.004 | 0.285 | 0.028 | 80.5 | 11.7 | 1.1 | 1.1 | 0.00065 | 39.31 | 2.81880 | 1.94 | 0.0485 |
| ***27877-07E*** | 3.8 | 0.02240 | 0.00004 | 772.7 | 58.3 | 2315.5 | 6.5 | 36.2 | 2.2 | 1.3 | 4.7 | 1.2 | 0.8 | 1.07 | 12.4 | 0.004 | 0.014 | 0.183 | 0.104 | 54.8 | 7.5 | 4.2 | 4.2 | 0.00151 | 67.28 | 3.00004 | 7.56 | 0.1122 |
| ***27877-07F*** | 5.4 | 0.02240 | 0.00004 | 139.5 | 58.0 | 240.2 | 3.3 | 12.7 | 2.0 | 6.9 | 4.6 | -0.9 | 0.8 | 0.11 | 1.3 | 0.201 | 0.135 | 1.692 | 1.028 | 291.5 | 69.2 | 42.0 | 42.0 | - 0.00641 | 99.01 | 1.72286 | 41.64 | 0.4201 |
| | | | | | | | | | | | | | | | | | | | | | | | | | | | | |
| OLOR16/SKG-2p1 - Aliquot #8 | | | | | | | | | | | | | | | | | | | | | | | | | | | | |
| 27877-08A | 1.0 | 0.02240 | 0.00004 | 7629.3 | 60.7 | 372.1 | 4.7 | 12.7 | 1.6 | -7.4 | 4.7 | 23.1 | 1.0 | 0.17 | 4.3 | -0.139 | 0.089 | 1.930 | 0.791 | 9.4 | 78.9 | 32.3 | 32.3 | 0.00303 | 4.24 | 0.04880 | 1.50 | 0.0992 |
| ***27877-08B*** | 1.4 | 0.02240 | 0.00004 | 8026.5 | 58.3 | 2086.3 | 9.6 | 40.3 | 1.9 | -4.9 | 4.7 | 25.2 | 0.9 | 0.96 | 24.1 | -0.017 | 0.016 | 0.245 | 0.135 | 6.4 | 10.0 | 5.5 | 5.5 | 0.00314 | 3.73 | 0.26006 | 0.86 | 0.1645 |
| ***27877-08C*** | 2.0 | 0.02240 | 0.00004 | 9887.6 | 59.6 | 5404.4 | 14.9 | 95.5 | 2.3 | -17.6 | 4.7 | 27.9 | 1.0 | 2.49 | 62.6 | -0.023 | 0.006 | 0.288 | 0.054 | 15.7 | 11.8 | 2.2 | 2.2 | 0.00282 | 3.48 | 0.54691 | 0.66 | 0.1575 |
| ***27877-08D*** | 2.7 | 0.02240 | 0.00004 | 371.5 | 56.9 | 719.1 | 4.9 | 9.2 | 1.4 | -14.6 | 4.7 | 0.6 | 0.8 | 0.33 | 8.3 | -0.143 | 0.046 | 0.279 | 0.333 | 54.1 | 11.4 | 13.6 | 13.6 | 0.00154 | 137.66 | 1.93782 | 15.34 | 0.1112 |
| ***27877-08E*** | 3.8 | 0.02240 | 0.00004 | 38.2 | 57.9 | 58.2 | 1.9 | -6.0 | 1.5 | -1.3 | 4.7 | -1.2 | 0.8 | 0.03 | 0.7 | -0.154 | 0.561 | 6.635 | 4.193 | 1011.2 | 271.5 | 171.5 | 171.6 | - 0.03052 | 166.05 | 1.52413 | 151.51 | 0.9120 |
| | | | | | | | | | | | | | | | | | | | | | | | | | | | | |
| OLOR16/SKG-2p1 - Aliquot #9 | | | | | | | | | | | | | | | | | | | | | | | | | | | | |
| ***27877-09A*** | 1.0 | 0.02240 | 0.00004 | 7455.7 | 59.4 | 234.6 | 3.3 | 6.2 | 1.5 | 48.9 | 4.8 | 26.0 | 1.0 | 0.11 | 2.9 | 1.463 | 0.144 | -1.284 | 1.249 | -4.0 | -52.5 | 51.1 | 51.1 | 0.00348 | 3.78 | 0.03147 | 1.62 | 0.1034 |
| ***27877-09B*** | 1.4 | 0.02240 | 0.00004 | 4636.4 | 58.6 | 970.7 | 5.4 | 16.4 | 1.8 | 175.7 | 4.8 | 14.8 | 0.9 | 0.45 | 12.0 | 1.271 | 0.036 | 0.283 | 0.271 | 5.9 | 11.6 | 11.1 | 11.1 | 0.00315 | 6.02 | 0.20938 | 1.38 | 0.1922 |
| ***27877-09C*** | 2.0 | 0.02240 | 0.00004 | 8241.1 | 60.1 | 4731.0 | 11.7 | 75.4 | 2.4 | 13.7 | 4.8 | 23.0 | 1.0 | 2.18 | 58.5 | 0.020 | 0.007 | 0.293 | 0.062 | 16.8 | 12.0 | 2.6 | 2.6 | 0.00279 | 4.28 | 0.57441 | 0.77 | 0.1615 |
| ***27877-09D*** | 2.7 | 0.02240 | 0.00004 | 1132.9 | 57.3 | 1566.3 | 7.6 | 20.9 | 1.9 | 0.7 | 4.7 | 2.5 | 0.8 | 0.72 | 19.4 | 0.003 | 0.021 | 0.240 | 0.155 | 33.1 | 9.8 | 6.4 | 6.4 | 0.00224 | 31.67 | 1.38359 | 5.08 | 0.1589 |
| ***27877-09E*** | 3.8 | 0.02240 | 0.00004 | 241.0 | 57.0 | 580.9 | 4.2 | 4.7 | 1.5 | 2.6 | 4.6 | -0.9 | 0.8 | 0.27 | 7.2 | 0.031 | 0.056 | 0.879 | 0.408 | 212.0 | 35.9 | 16.7 | 16.7 | - 0.00375 | 88.51 | 2.41330 | 23.66 | 0.2671 |
| | | | | | | | | | | | | | | | | | | | | | | | | | | | | |
| OLOR16/SKG-2p1 - Aliquot #10 | | | | | | | | | | | | | | | | | | | | | | | | | | | | |
| 27877-10A | 1.0 | 0.02240 | 0.00004 | 24323.0 | 60.3 | 182.1 | 2.6 | 22.6 | 1.5 | 7.5 | 4.7 | 82.7 | 1.2 | 0.08 | 1.4 | 0.287 | 0.181 | -2.011 | 1.966 | -1.5 | -82.3 | 80.4 | 80.4 | 0.00340 | 1.45 | 0.00749 | 1.43 | 0.0296 |
| 27877-10B | 1.4 | 0.02240 | 0.00004 | 2356.8 | 57.1 | 1025.3 | 6.4 | 14.8 | 1.4 | 6.1 | 4.6 | 4.0 | 0.8 | 0.47 | 7.7 | 0.041 | 0.032 | 1.133 | 0.240 | 49.3 | 46.4 | 9.8 | 9.8 | 0.00170 | 20.21 | 0.43526 | 2.50 | 0.1161 |
| 27877-10C | 2.0 | 0.02240 | 0.00004 | 2336.8 | 56.4 | 3278.8 | 9.0 | 51.2 | 2.1 | 15.0 | 4.6 | 2.7 | 0.8 | 1.51 | 24.6 | 0.032 | 0.010 | 0.469 | 0.076 | 65.9 | 19.2 | 3.1 | 3.1 | 0.00114 | 30.35 | 1.40427 | 2.43 | 0.0790 |
| ***27877-10D*** | 2.7 | 0.02240 | 0.00004 | 1210.5 | 57.4 | 4741.0 | 12.2 | 66.3 | 2.0 | 3.9 | 4.7 | 0.7 | 0.8 | 2.18 | 35.6 | 0.005 | 0.007 | 0.211 | 0.051 | 82.8 | 8.6 | 2.1 | 2.1 | 0.00058 | 112.36 | 3.92222 | 4.75 | 0.0422 |
| ***27877-10E*** | 3.8 | 0.02240 | 0.00004 | 1214.0 | 56.5 | 3771.5 | 10.7 | 66.1 | 2.3 | 0.6 | 4.7 | 1.4 | 0.8 | 1.74 | 28.3 | 0.001 | 0.009 | 0.211 | 0.063 | 65.5 | 8.6 | 2.6 | 2.6 | 0.00116 | 55.72 | 3.11063 | 4.67 | 0.0834 |
| ***27877-10F*** | 5.4 | 0.02240 | 0.00004 | 64.4 | 55.8 | 305.2 | 4.2 | 9.2 | 1.6 | -7.5 | 4.6 | 0.9 | 0.8 | 0.14 | 2.3 | -0.173 | 0.106 | -0.711 | 0.766 | -337.3 | -29.1 | 31.3 | 31.3 | 0.01465 | 118.46 | 4.74414 | 86.77 | 0.7324 |
| | | | | | | | | | | | | | | | | | | | | | | | | | | | | |
| OLOR16/SKG-2p1 - Aliquot #11 | | | | | | | | | | | | | | | | | | | | | | | | | | | | |
| ***27877-11A*** | 1.0 | 0.02240 | 0.00004 | 7245.5 | 56.5 | 241.8 | 2.9 | 15.3 | 1.6 | 1.9 | 4.6 | 24.2 | 0.9 | 0.11 | 2.3 | 0.054 | 0.134 | 0.026 | 1.150 | 0.1 | 1.0 | 47.0 | 47.0 | 0.00335 | 3.84 | 0.03338 | 1.44 | 0.1098 |
| ***27877-11B*** | 1.4 | 0.02240 | 0.00004 | 3088.6 | 55.2 | 821.6 | 5.6 | 14.9 | 1.7 | 59.1 | 4.5 | 8.3 | 0.8 | 0.38 | 7.7 | 0.507 | 0.039 | 0.781 | 0.313 | 20.8 | 31.9 | 12.8 | 12.8 | 0.00265 | 10.42 | 0.26611 | 1.92 | 0.1602 |
| ***27877-11C*** | 2.0 | 0.02240 | 0.00004 | 2173.4 | 54.5 | 3836.5 | 12.4 | 61.9 | 2.2 | 10.0 | 4.5 | 2.1 | 0.8 | 1.77 | 36.1 | 0.018 | 0.008 | 0.406 | 0.065 | 71.7 | 16.6 | 2.7 | 2.7 | 0.00095 | 40.04 | 1.76680 | 2.53 | 0.0621 |
| ***27877-11D*** | 2.7 | 0.02240 | 0.00004 | 1350.8 | 56.1 | 3231.3 | 9.4 | 45.4 | 1.5 | 3.8 | 4.4 | 1.0 | 0.7 | 1.49 | 30.4 | 0.008 | 0.010 | 0.326 | 0.071 | 78.0 | 13.3 | 2.9 | 2.9 | 0.00074 | 75.44 | 2.39473 | 4.17 | 0.0549 |
| ***27877-11E*** | 3.8 | 0.02240 | 0.00004 | 511.9 | 57.1 | 1755.7 | 7.2 | 27.5 | 1.5 | 1.0 | 4.4 | 0.7 | 0.8 | 0.81 | 16.5 | 0.004 | 0.018 | 0.170 | 0.134 | 58.3 | 6.9 | 5.5 | 5.5 | 0.00140 | 107.61 | 3.43458 | 11.17 | 0.1037 |
| ***27877-11F*** | 5.4 | 0.02240 | 0.00004 | 164.1 | 56.1 | 727.4 | 6.2 | 8.5 | 1.6 | -0.6 | 4.4 | -0.3 | 0.7 | 0.34 | 6.9 | -0.006 | 0.043 | 0.366 | 0.314 | 162.2 | 15.0 | 12.8 | 12.8 | - 0.00209 | 219.37 | 4.43854 | 34.22 | 0.1559 |

| | | | | | | | | | | | | | | | | | | | | | | | | | | | | |
|---|---|---|---|---|---|---|---|---|---|---|---|---|---|---|---|---|---|---|---|---|---|---|---|---|---|---|---|---|
| OLOR16/SKG-2p1 - Aliquot #12 | | | | | | | | | | | | | | | | | | | | | | | | | | | | |
| ***27877-12A*** | 1.0 | 0.02240 | 0.00004 | 2218.8 | 57.0 | 160.5 | 3.2 | 2.7 | 1.7 | 6.7 | 4.5 | 8.7 | 0.8 | 0.07 | 2.4 | 0.293 | 0.199 | -2.281 | 1.557 | -16.5 | -93.3 | 63.7 | 63.7 | 0.00390 | 9.76 | 0.07238 | 3.26 | 0.2078 |
| ***27877-12B*** | 1.4 | 0.02240 | 0.00004 | 1588.0 | 56.3 | 766.5 | 4.5 | 9.7 | 1.7 | 83.6 | 4.5 | 4.1 | 0.9 | 0.35 | 11.5 | 0.770 | 0.042 | 0.497 | 0.341 | 24.0 | 20.3 | 14.0 | 14.0 | 0.00255 | 21.46 | 0.48280 | 3.59 | 0.1629 |
| ***27877-12C*** | 2.0 | 0.02240 | 0.00004 | 1505.0 | 55.9 | 3566.5 | 12.4 | 54.7 | 2.1 | 10.5 | 4.4 | 2.3 | 0.8 | 1.65 | 53.5 | 0.020 | 0.009 | 0.228 | 0.067 | 54.0 | 9.3 | 2.7 | 2.7 | 0.00154 | 33.89 | 2.37222 | 3.73 | 0.1092 |
| ***27877-12D*** | 2.7 | 0.02240 | 0.00004 | 538.9 | 57.1 | 1857.8 | 7.5 | 24.3 | 1.6 | 0.3 | 4.4 | 0.4 | 0.7 | 0.86 | 27.9 | 0.001 | 0.017 | 0.219 | 0.121 | 75.6 | 9.0 | 4.9 | 4.9 | 0.00082 | 165.60 | 3.45157 | 10.61 | 0.0640 |
| ***27877-12E*** | 3.8 | 0.02240 | 0.00004 | 64.2 | 57.4 | 318.5 | 4.0 | 3.9 | 1.3 | 7.6 | 4.4 | 0.9 | 0.7 | 0.15 | 4.8 | 0.169 | 0.098 | -0.640 | 0.717 | -317.9 | -26.2 | 29.4 | 29.4 | 0.01400 | 121.80 | 4.96933 | 89.55 | 0.7351 |
| | | | | | | | | | | | | | | | | | | | | | | | | | | | | |
| OLOR16/SKG-2p1 - Aliquot #13 | | | | | | | | | | | | | | | | | | | | | | | | | | | | |
| 27877-13A | 1.0 | 0.02240 | 0.00004 | 2581.8 | 58.1 | 99.2 | 2.2 | -1.5 | 1.4 | 19.1 | 4.5 | 6.4 | 0.8 | 0.05 | 1.5 | 1.364 | 0.319 | 6.671 | 2.478 | 25.6 | 272.9 | 101.4 | 101.4 | 0.00249 | 12.61 | 0.03841 | 3.19 | 0.1260 |
| 27877-13B | 1.4 | 0.02240 | 0.00004 | 1730.5 | 58.1 | 508.0 | 5.2 | 7.0 | 1.7 | -2.0 | 4.4 | 3.7 | 0.8 | 0.23 | 7.5 | -0.029 | 0.061 | 1.242 | 0.464 | 36.5 | 50.8 | 19.0 | 19.0 | 0.00213 | 21.04 | 0.29373 | 3.51 | 0.1524 |
| ***27877-13C*** | 2.0 | 0.02240 | 0.00004 | 1510.3 | 57.1 | 2994.2 | 7.7 | 39.8 | 1.7 | -5.2 | 4.4 | 3.4 | 0.7 | 1.38 | 44.0 | -0.013 | 0.010 | 0.168 | 0.075 | 33.4 | 6.9 | 3.1 | 3.1 | 0.00223 | 21.90 | 1.98439 | 3.79 | 0.1722 |
| ***27877-13D*** | 2.7 | 0.02240 | 0.00004 | 1234.5 | 57.4 | 3106.4 | 10.3 | 42.2 | 1.7 | 8.9 | 4.6 | 2.1 | 0.8 | 1.43 | 45.7 | 0.020 | 0.010 | 0.200 | 0.080 | 50.3 | 8.2 | 3.3 | 3.3 | 0.00166 | 39.83 | 2.51902 | 4.66 | 0.1164 |
| ***27877-13E*** | 3.8 | 0.02240 | 0.00004 | -7.4 | 56.8 | 93.5 | 2.7 | -1.2 | 1.5 | 22.8 | 4.5 | 0.7 | 0.8 | 0.04 | 1.4 | 1.723 | 0.346 | -2.194 | 2.613 | 2754.6 | -89.8 | 106.9 | 106.9 | - 0.08891 | 772.38 | -12.55544 | 762.98 | 0.9878 |
| | | | | | | | | | | | | | | | | | | | | | | | | | | | | |
| OLOR16/SKG-2p1 - Aliquot #14 | | | | | | | | | | | | | | | | | | | | | | | | | | | | |
| 27881-01A | 1.0 | 0.02220 | 0.00005 | 4875.9 | 56.8 | 135.6 | 2.7 | 7.1 | 1.5 | 21.1 | 4.5 | 14.1 | 0.8 | 0.06 | 1.7 | 1.157 | 0.248 | 4.972 | 1.866 | 13.8 | 202.2 | 75.9 | 75.9 | 0.00289 | 5.98 | 0.02781 | 2.33 | 0.0976 |
| ***27881-01B*** | 1.4 | 0.02220 | 0.00005 | 6158.8 | 57.2 | 1128.6 | 7.0 | 21.5 | 1.8 | 20.0 | 4.5 | 19.1 | 0.9 | 0.53 | 14.3 | 0.131 | 0.030 | 0.422 | 0.249 | 7.7 | 17.2 | 10.1 | 10.1 | 0.00309 | 4.93 | 0.18334 | 1.12 | 0.1571 |
| ***27881-01C*** | 2.0 | 0.02220 | 0.00005 | 13911.0 | 58.5 | 4986.8 | 14.9 | 82.8 | 2.7 | -1.8 | 4.5 | 40.1 | 1.0 | 2.33 | 63.3 | -0.003 | 0.007 | 0.388 | 0.064 | 13.9 | 15.8 | 2.6 | 2.6 | 0.00288 | 2.65 | 0.35868 | 0.52 | 0.1298 |
| ***27881-01D*** | 2.7 | 0.02220 | 0.00005 | 2059.6 | 54.4 | 1475.8 | 6.7 | 18.3 | 1.6 | -6.6 | 4.5 | 5.3 | 0.8 | 0.69 | 18.7 | -0.034 | 0.023 | 0.320 | 0.158 | 22.9 | 13.0 | 6.4 | 6.4 | 0.00258 | 14.53 | 0.71706 | 2.68 | 0.1793 |
| ***27881-01E*** | 3.8 | 0.02220 | 0.00005 | 125.4 | 55.4 | 153.7 | 2.9 | 13.9 | 1.6 | -8.2 | 4.5 | 0.2 | 0.9 | 0.07 | 2.0 | -0.397 | 0.218 | 0.498 | 1.692 | 61.1 | 20.2 | 68.8 | 68.8 | 0.00130 | 522.59 | 1.22671 | 44.19 | 0.0844 |
| | | | | | | | | | | | | | | | | | | | | | | | | | | | | |
| OLOR16/SKG-2p1 - Aliquot #15 | | | | | | | | | | | | | | | | | | | | | | | | | | | | |
| ***27881-02A*** | 1.0 | 0.02220 | 0.00005 | 3214.2 | 56.9 | 164.5 | 3.0 | 12.0 | 1.9 | -1.6 | 4.6 | 10.2 | 0.9 | 0.08 | 2.1 | -0.072 | 0.206 | 0.983 | 1.608 | 5.0 | 40.0 | 65.4 | 65.4 | 0.00318 | 8.65 | 0.05120 | 2.53 | 0.1430 |
| ***27881-02B*** | 1.4 | 0.02220 | 0.00005 | 2117.0 | 55.8 | 810.9 | 5.0 | 12.5 | 1.7 | -2.6 | 4.7 | 6.2 | 0.8 | 0.38 | 10.4 | -0.024 | 0.043 | 0.333 | 0.312 | 12.8 | 13.6 | 12.7 | 12.7 | 0.00292 | 13.64 | 0.38329 | 2.71 | 0.1881 |
| ***27881-02C*** | 2.0 | 0.02220 | 0.00005 | 2608.2 | 56.1 | 2095.4 | 7.6 | 34.8 | 1.4 | 10.5 | 4.6 | 6.7 | 0.8 | 0.98 | 27.0 | 0.037 | 0.016 | 0.293 | 0.120 | 23.5 | 11.9 | 4.9 | 4.9 | 0.00256 | 12.44 | 0.80396 | 2.18 | 0.1707 |
| ***27881-02D*** | 2.7 | 0.02220 | 0.00005 | 2080.7 | 55.8 | 3013.0 | 9.2 | 38.8 | 2.1 | -0.2 | 4.7 | 3.5 | 0.8 | 1.41 | 38.8 | -0.001 | 0.012 | 0.343 | 0.080 | 49.7 | 13.9 | 3.3 | 3.3 | 0.00168 | 22.73 | 1.44927 | 2.70 | 0.1173 |
| ***27881-02E*** | 3.8 | 0.02220 | 0.00005 | 874.4 | 54.5 | 1500.1 | 7.3 | 20.5 | 2.0 | -2.6 | 4.5 | 1.7 | 0.8 | 0.70 | 19.3 | -0.013 | 0.022 | 0.250 | 0.161 | 43.0 | 10.2 | 6.6 | 6.6 | 0.00191 | 47.80 | 1.71714 | 6.25 | 0.1300 |
| ***27881-02F*** | 5.4 | 0.02220 | 0.00005 | 30.1 | 54.5 | 184.1 | 2.8 | 4.3 | 1.4 | 14.1 | 4.5 | -0.6 | 0.7 | 0.09 | 2.4 | 0.569 | 0.183 | 1.169 | 1.234 | 717.6 | 47.6 | 50.2 | 50.2 | - 0.02069 | 217.08 | 6.13684 | 181.56 | 0.8363 |
| | | | | | | | | | | | | | | | | | | | | | | | | | | | | |
| OLOR16/SKG-2p1 - Aliquot #16 | | | | | | | | | | | | | | | | | | | | | | | | | | | | |
| ***27881-03A*** | 1.0 | 0.02220 | 0.00005 | 12733.9 | 57.3 | 199.0 | 3.3 | 16.1 | 1.8 | -2.2 | 4.5 | 43.9 | 1.0 | 0.09 | 2.5 | -0.081 | 0.170 | -1.838 | 1.488 | -2.9 | -74.8 | 60.5 | 60.5 | 0.00345 | 2.26 | 0.01564 | 1.72 | 0.0519 |
| ***27881-03B*** | 1.4 | 0.02220 | 0.00005 | 7106.4 | 55.9 | 1603.4 | 9.3 | 27.5 | 1.5 | 4.8 | 4.5 | 21.8 | 0.9 | 0.75 | 20.2 | 0.022 | 0.021 | 0.378 | 0.176 | 8.5 | 15.4 | 7.2 | 7.2 | 0.00306 | 4.34 | 0.22575 | 0.98 | 0.1459 |
| ***27881-03C*** | 2.0 | 0.02220 | 0.00005 | 6589.9 | 57.1 | 5188.3 | 13.6 | 76.4 | 2.2 | 4.1 | 4.5 | 16.8 | 0.8 | 2.43 | 65.5 | 0.005 | 0.007 | 0.303 | 0.049 | 23.9 | 12.3 | 2.0 | 2.0 | 0.00255 | 5.04 | 0.78784 | 0.91 | 0.1645 |
| ***27881-03D*** | 2.7 | 0.02220 | 0.00005 | 798.7 | 56.3 | 777.9 | 5.4 | 11.1 | 1.5 | 0.6 | 4.5 | 1.3 | 0.8 | 0.36 | 9.8 | 0.006 | 0.044 | 0.532 | 0.301 | 51.8 | 21.6 | 12.2 | 12.2 | 0.00161 | 59.48 | 0.97466 | 7.08 | 0.1179 |
| ***27881-03E*** | 3.8 | 0.02220 | 0.00005 | 104.9 | 54.5 | 152.4 | 2.7 | -1.2 | 1.4 | 2.4 | 4.5 | -0.5 | 0.8 | 0.07 | 1.9 | 0.116 | 0.220 | 1.682 | 1.549 | 244.6 | 68.4 | 63.0 | 63.0 | - 0.00484 | 160.15 | 1.45442 | 51.98 | 0.3242 |
| | | | | | | | | | | | | | | | | | | | | | | | | | | | | |
| OLOR16/SKG-2p1 - Aliquot #17 | | | | | | | | | | | | | | | | | | | | | | | | | | | | |
| 27881-04A | 1.0 | 0.02220 | 0.00005 | 6126.0 | 55.2 | 137.6 | 2.5 | 8.3 | 1.6 | -3.8 | 4.6 | 19.0 | 0.9 | 0.06 | 0.7 | -0.206 | 0.247 | 3.255 | 1.961 | 7.3 | 132.4 | 79.8 | 79.8 | 0.00310 | 4.74 | 0.02247 | 2.01 | 0.0854 |
| 27881-04B | 1.4 | 0.02220 | 0.00005 | 3120.1 | 55.2 | 870.6 | 5.9 | 12.3 | 1.7 | 2.1 | 4.6 | 5.3 | 0.8 | 0.41 | 4.7 | 0.017 | 0.039 | 1.767 | 0.282 | 49.3 | 71.9 | 11.5 | 11.5 | 0.00170 | 15.22 | 0.27917 | 1.89 | 0.1086 |
| ***27881-04C*** | 2.0 | 0.02220 | 0.00005 | 3364.9 | 55.7 | 6195.0 | 10.2 | 85.4 | 2.3 | -8.5 | 4.6 | 4.9 | 0.8 | 2.90 | 33.8 | -0.011 | 0.006 | 0.307 | 0.040 | 56.6 | 12.5 | 1.6 | 1.6 | 0.00145 | 16.66 | 1.84278 | 1.66 | 0.0989 |
| ***27881-04D*** | 2.7 | 0.02220 | 0.00005 | 2926.3 | 55.7 | 6755.8 | 17.3 | 96.4 | 2.4 | -2.9 | 4.6 | 4.0 | 0.8 | 3.16 | 36.8 | -0.004 | 0.005 | 0.256 | 0.035 | 59.1 | 10.4 | 1.4 | 1.4 | 0.00137 | 19.59 | 2.31108 | 1.92 | 0.0964 |
| ***27881-04E*** | 3.8 | 0.02220 | 0.00005 | 1321.3 | 55.4 | 4063.2 | 13.6 | 51.5 | 1.5 | -10.6 | 4.6 | 1.1 | 0.8 | 1.90 | 22.1 | -0.020 | 0.008 | 0.243 | 0.060 | 75.0 | 9.9 | 2.4 | 2.4 | 0.00084 | 71.82 | 3.07894 | 4.21 | 0.0582 |
| ***27881-04F*** | 5.4 | 0.02220 | 0.00005 | 237.3 | 54.3 | 330.9 | 3.1 | 2.7 | 1.4 | 5.0 | 4.6 | 0.4 | 0.8 | 0.15 | 1.8 | 0.112 | 0.104 | 0.322 | 0.722 | 44.9 | 13.1 | 29.4 | 29.4 | 0.00185 | 179.52 | 1.39571 | 22.89 | 0.1273 |
| | | | | | | | | | | | | | | | | | | | | | | | | | | | | |
| OLOR16/SKG-2p1 - Aliquot #18 | | | | | | | | | | | | | | | | | | | | | | | | | | | | |
| 27881-05A | 1.0 | 0.02220 | 0.00005 | 9753.5 | 58.9 | 306.2 | 3.5 | 24.7 | 1.8 | -20.9 | 5.0 | 24.9 | 0.9 | 0.14 | 3.1 | -0.510 | 0.122 | 7.523 | 0.940 | 23.6 | 306.0 | 38.2 | 38.2 | 0.00256 | 3.82 | 0.03141 | 1.28 | 0.0747 |
| ***27881-05B*** | 1.4 | 0.02220 | 0.00005 | 3034.9 | 56.9 | 1581.2 | 6.1 | 30.6 | 1.7 | 12.0 | 4.7 | 7.3 | 0.9 | 0.74 | 15.9 | 0.056 | 0.022 | 0.534 | 0.167 | 27.9 | 21.7 | 6.8 | 6.8 | 0.00242 | 11.90 | 0.52132 | 1.91 | 0.1543 |
| ***27881-05C*** | 2.0 | 0.02220 | 0.00005 | 2841.7 | 55.9 | 5017.5 | 10.6 | 70.5 | 2.2 | 10.3 | 4.7 | 2.2 | 0.8 | 2.35 | 50.5 | 0.015 | 0.007 | 0.438 | 0.049 | 77.4 | 17.8 | 2.0 | 2.0 | 0.00076 | 37.48 | 1.76727 | 1.98 | 0.0522 |
| ***27881-05D*** | 2.7 | 0.02220 | 0.00005 | 1011.8 | 54.9 | 2739.4 | 9.3 | 36.6 | 2.0 | -12.6 | 4.6 | 1.3 | 0.8 | 1.28 | 27.6 | -0.035 | 0.013 | 0.230 | 0.087 | 62.3 | 9.3 | 3.6 | 3.6 | 0.00126 | 61.39 | 2.71049 | 5.44 | 0.0882 |
| ***27881-05E*** | 3.8 | 0.02220 | 0.00005 | 155.7 | 54.6 | 286.1 | 3.6 | -1.7 | 1.5 | -3.4 | 4.7 | 0.7 | 0.8 | 0.13 | 2.9 | -0.090 | 0.122 | -0.204 | 0.861 | -37.5 | -8.3 | 35.0 | 35.0 | 0.00461 | 117.64 | 1.83910 | 35.08 | 0.2978 |
| | | | | | | | | | | | | | | | | | | | | | | | | | | | | |
| Notes: | | | | | | | | | | | | | | | | | | | | | | | | | | | | |
| Lab IDs in bold-italic font are part of the incremental heating plateau. | | | | | | | | | | | | | | | | | | | | | | | | | | | | |

**Table S5.** Single-crystal incremental heating ('SCIH') plateau and isochron results.

| | | Integrated Step-heating (Total-gas) Results | | | | | | | | | Plateau Results | | | | | | | Isochron Results | | | | | |
|---|---|---|---|---|---|---|---|---|---|---|---|---|---|---|---|---|---|---|---|---|---|---|---|
| Sample | Lab ID | *n* | Mol $^{39}Ar$ | Ca/K | | $^{40}Ar^*/^{39}K$ | | % $^{40}Ar^*$ | Age | | *n* | % Gas | MSWD | Prob. | Steps | Age | | $^{40}Ar/^{36}Ar$ trapped | | Age | | MSWD | Prob |
| | | | | ± 1σ se | | ± 1σ mse | | | (Ma ± 1σ mse) | | | | | | | (Ma ± 1σ mse) | | ± 1σ mse | | (Ma ± 1σ mse) | | | |
| OLOR16/SKG-1pB1 | 27878-01 | 6 | 5.48E-16 | -0.0183 | 0.0183 | 0.5674 | 0.0573 | 29.0 | 23.200 | 2.300 | No plateaus | | | | | | | | | | | | |
| OLOR16/SKG-1pB1 | 27878-02 | 5 | 3.06E-16 | 0.0055 | 0.0303 | 0.5608 | 0.0975 | 46.5 | 22.900 | 4.000 | 5 | 100.0 | 1.1 | 0.36 | A-E | 22.2 | 2.7 | 300.1 | 3.0 | 22.1 | 2.6 | 1.4 | 0.26 |
| OLOR16/SKG-1pB1 | 27878-03 | 6 | 6.83E-16 | 0.0138 | 0.1898 | 0.7070 | 0.0471 | 22.6 | 28.900 | 1.900 | 3 | 60.1 | 1.5 | 0.22 | D-F | 17.1 | 2.2 | 212.6 | 37.9 | 40.1 | 10.0 | 0.3 | 0.57 |
| OLOR16/SKG-1pB1 | 27878-04 | 5 | 2.29E-16 | -0.0595 | 0.2034 | 0.3174 | 0.1196 | 16.3 | 13.000 | 4.900 | 5 | 100.0 | 0.7 | 0.58 | A-E | 15.6 | 3.5 | 308.7 | 17.7 | 13.2 | 4.9 | 0.6 | 0.60 |
| OLOR16/SKG-1pB1 | 27878-05 | 7 | 8.07E-16 | 0.0230 | 0.0047 | 0.0206 | 0.1172 | 14.0 | -0.800 | 4.800 | 5 | 97.6 | 1.0 | 0.43 | C-G | 17.2 | 1.6 | 302.5 | 4.2 | 15.7 | 2.2 | 1.0 | 0.40 |
| OLOR16/SKG-1pB1 | 27878-06 | 7 | 2.25E-16 | 0.0491 | 0.1280 | 0.4464 | 0.1381 | 21.2 | 18.300 | 5.600 | 7 | 100.0 | 0.9 | 0.50 | A-G | 15.5 | 4.6 | 317.9 | 12.6 | 12.2 | 4.5 | 0.5 | 0.81 |
| OLOR16/SKG-1pB1 | 27878-07 | 5 | 3.42E-16 | 0.0411 | 0.0096 | 0.0551 | 0.1866 | 4.7 | 2.300 | 7.600 | 5 | 100.0 | 1.9 | 0.10 | A-E | 12.6 | 3.3 | 296.9 | 1.6 | 13.5 | 2.3 | 2.2 | 0.09 |
| OLOR16/SKG-1pB1 | 27878-08 | 4 | 2.46E-16 | -0.0001 | 0.0102 | 0.4045 | 0.1193 | 2.8 | 16.500 | 4.900 | 3 | 98.1 | 1.2 | 0.31 | B-D | 13.2 | 4.0 | 296.1 | 22.7 | 15.7 | 9.2 | 2.2 | 0.14 |
| OLOR16/SKG-1pB1 | 27878-09 | 5 | 3.05E-16 | 0.0200 | 0.0113 | 0.4757 | 0.0971 | 12.8 | 19.500 | 4.000 | 5 | 100.0 | 0.6 | 0.64 | A-E | 21.2 | 3.1 | 291.5 | 6.9 | 26.0 | 5.3 | 0.5 | 0.69 |
| OLOR16/SKG-1pB1 | 27878-10 | 6 | 3.30E-16 | -0.0151 | 0.0221 | 0.5053 | 0.0974 | 28.8 | 20.700 | 4.000 | 6 | 100.0 | 2.0 | 0.08 | A-F | 16.9 | 3.4 | 301.1 | 4.1 | 16.4 | 2.3 | 2.2 | 0.06 |
| OLOR16/SKG-1pB1 | 27878-11 | 6 | 1.19E-16 | 0.1304 | 0.0340 | 1.8255 | 0.2523 | 25.4 | 75.000 | 10.000 | No plateaus | | | | | | | | | | | | |
| OLOR16/SKG-2p1 | 27877-01 | 6 | 5.16E-16 | 0.0143 | 0.0065 | 0.1033 | 0.0537 | 29.8 | 4.200 | 2.200 | 5 | 98.4 | 1.3 | 0.27 | B-F | 9.3 | 1.6 | 278.2 | 21.1 | 10.5 | 1.6 | 1.4 | 0.24 |
| OLOR16/SKG-2p1 | 27877-02 | 5 | 3.21E-16 | 0.0227 | 0.0097 | 0.4030 | 0.0847 | 23.4 | 16.500 | 3.500 | 5 | 100.0 | 1.3 | 0.26 | A-E | 14.8 | 3.1 | 288.9 | 8.5 | 18.9 | 4.2 | 1.0 | 0.39 |
| OLOR16/SKG-2p1 | 27877-03 | 5 | 2.99E-16 | -0.0307 | 0.0299 | 0.2543 | 0.0905 | 17.3 | 10.400 | 3.700 | 5 | 100.0 | 0.6 | 0.64 | A-E | 8.7 | 2.7 | 297.4 | 12.9 | 9.1 | 3.8 | 0.8 | 0.52 |
| OLOR16/SKG-2p1 | 27877-04 | 5 | 2.99E-16 | 0.1539 | 0.3161 | 0.3903 | 0.0881 | 97.6 | 16.000 | 3.600 | 5 | 100.0 | 0.3 | 0.89 | A-E | 14.9 | 2.6 | 297.3 | 25.4 | 15.0 | 2.6 | 0.4 | 0.76 |
| OLOR16/SKG-2p1 | 27877-05 | 5 | 2.65E-16 | -0.0066 | 0.0079 | 0.0691 | 0.1043 | 15.0 | 2.800 | 4.300 | 5 | 100.0 | 0.5 | 0.73 | A-E | 7.2 | 2.5 | 293.4 | 8.7 | 7.9 | 2.5 | 0.6 | 0.63 |
| OLOR16/SKG-2p1 | 27877-06 | 5 | 3.26E-16 | -0.0130 | 0.0094 | 0.2973 | 0.0840 | 71.7 | 12.200 | 3.400 | 5 | 100.0 | 0.5 | 0.76 | A-E | 10.3 | 1.9 | 300.5 | 3.9 | 10.3 | 1.9 | 0.5 | 0.65 |
| OLOR16/SKG-2p1 | 27877-07 | 6 | 8.58E-16 | -0.0128 | 0.0336 | 0.3229 | 0.0343 | 56.2 | 13.200 | 1.400 | 6 | 100.0 | 1.1 | 0.37 | A-F | 12.2 | 1.0 | 313.3 | 11.1 | 11.4 | 1.1 | 0.8 | 0.55 |
| OLOR16/SKG-2p1 | 27877-08 | 5 | 3.98E-16 | -0.0237 | 0.0085 | 0.3902 | 0.0702 | 23.1 | 16.000 | 2.900 | 4 | 95.7 | 0.8 | 0.50 | B-E | 11.6 | 2.0 | 292.8 | 19.6 | 13.0 | 5.0 | 0.2 | 0.83 |
| OLOR16/SKG-2p1 | 27877-09 | 5 | 3.72E-16 | 0.0101 | 0.0499 | 0.2776 | 0.0740 | 32.1 | 11.400 | 3.000 | 5 | 100.0 | 0.9 | 0.44 | A-E | 12.0 | 2.3 | 286.0 | 10.0 | 14.6 | 2.9 | 0.7 | 0.56 |
| OLOR16/SKG-2p1 | 27877-10 | 6 | 6.13E-16 | 0.0022 | 0.0213 | 0.2939 | 0.0487 | 60.3 | 12.000 | 2.000 | 3 | 66.3 | 0.7 | 0.49 | D-F | 8.5 | 1.6 | 277.3 | 314.8 | 8.8 | 2.6 | 0.6 | 0.46 |
| OLOR16/SKG-2p1 | 27877-11 | 6 | 4.90E-16 | 0.0105 | 0.0271 | 0.3600 | 0.0571 | 72.0 | 14.700 | 2.300 | 6 | 100.0 | 0.9 | 0.46 | A-F | 14.5 | 1.8 | 302.4 | 11.1 | 14.6 | 1.8 | 1.1 | 0.36 |
| OLOR16/SKG-2p1 | 27877-12 | 5 | 3.08E-16 | 0.0019 | 0.0540 | 0.1545 | 0.0808 | 37.1 | 6.300 | 3.300 | 5 | 100.0 | 1.2 | 0.32 | A-E | 9.2 | 2.5 | 271.4 | 25.1 | 10.2 | 2.1 | 0.9 | 0.43 |
| OLOR16/SKG-2p1 | 27877-13 | 5 | 3.14E-16 | -0.0697 | 0.1541 | 0.3253 | 0.0789 | 78.7 | 13.300 | 3.200 | 3 | 91.1 | 0.5 | 0.63 | C-E | 7.4 | 2.2 | 230.9 | 186.6 | 10.0 | 7.0 | 0.0 | 0.89 |
| OLOR16/SKG-2p1 | 27881-01 | 5 | 3.69E-16 | -0.0048 | 0.0101 | 0.4610 | 0.0768 | 15.6 | 18.800 | 3.100 | 4 | 98.3 | 0.1 | 0.98 | B-E | 15.5 | 2.3 | 306.7 | 22.6 | 12.9 | 7.6 | 0.0 | 0.97 |
| OLOR16/SKG-2p1 | 27881-02 | 6 | 3.63E-16 | -0.0023 | 0.0282 | 0.3436 | 0.0779 | 52.4 | 14.000 | 3.200 | 6 | 100.0 | 0.2 | 0.97 | A-F | 13.0 | 2.5 | 305.7 | 23.1 | 12.4 | 3.1 | 0.1 | 0.98 |
| OLOR16/SKG-2p1 | 27881-03 | 5 | 3.71E-16 | 0.0068 | 0.0092 | 0.3137 | 0.0740 | 27.1 | 12.800 | 3.000 | 5 | 100.0 | 0.9 | 0.47 | A-E | 12.7 | 1.9 | 290.8 | 6.2 | 14.1 | 2.1 | 0.7 | 0.57 |
| OLOR16/SKG-2p1 | 27881-04 | 6 | 8.59E-16 | -0.0071 | 0.0071 | 0.3657 | 0.0331 | 60.7 | 14.900 | 1.300 | 4 | 94.5 | 0.4 | 0.75 | C-F | 11.1 | 1.0 | 441.7 | 203.2 | 7.5 | 4.8 | 0.1 | 0.93 |
| OLOR16/SKG-2p1 | 27881-05 | 5 | 4.64E-16 | 0.0352 | 0.0202 | 0.5959 | 0.0579 | 60.4 | 24.200 | 2.400 | 4 | 96.9 | 1.8 | 0.14 | B-E | 16.1 | 2.3 | 351.7 | 49.3 | 14.7 | 2.2 | 1.8 | 0.16 |

Notes:

mse: Modified standard error; the standard error expanded by root MSWD if MSWD>1.

MSWD: Mean square weighted deviation

Prob.: Probability that the MSWD is fully explained by analytical scatter; if below 5%, plateau or isochron is not considered valid.

$^{40}Ar/^{36}Ar$ trapped: 'Trapped' non-radiogenic argon ratio calculated from the isochron fit.

**Table S6.** Cumulative probability distributions for the time and duration of overflow and closed basin conditions of Kenya Rift lakes.

| River | Lake Basin | Age ranges as Cumulative Probability Distributions (CPD) in years cal. BP (probability)[1] | | |
|---|---|---|---|---|
| | | **mid-late Holocene closed-lake basin level** | **early Holocene overflow-lake level** | **late Pleistocene closed-lake basin level** |
| | Nakuru-Elmenteita | *One Sigma Ranges*:<br>1181 – 1380 (0.085651)<br>3279 – 3280 (0.000346)<br>3345 – 3404 (0.021324)<br>3428 – 3442 (0.004892)<br>3634 – 4085 (0.212959)<br>4298 – 4328 (0.010201)<br>4353 – 4370 (0.005998)<br>4384 – 4585 (0.080385)<br>4597 – 4611 (0.00493)<br>4767 – 4782 (0.005428)<br>7271 – 7516 (0.122416)<br>7537 – 7562 (0.009146)<br>7664 – 8369 (0.436325)<br>*Two Sigma Ranges*:<br>1058 – 1524 (0.103493)<br>3070 – 4836 (0.448508)<br>7180 – 7196 (0.0018)<br>7243 – 8418 (0.446199) | *One Sigma Ranges*:<br>8784 – 8831 (0.016846)<br>8863 – 8888 (0.009005)<br>8892 – 8918 (0.008841)<br>8954 – 8962 (0.002627)<br>8969 – 10160 (0.962681)<br>*Two Sigma Ranges*:<br>8592 – 10237 (0.775591)<br>10572 – 11356 (0.095099)<br>11378 – 11387 (0.000668)<br>11623 – 11677 (0.004142)<br>11692 – 12548 (0.124501) | *One Sigma Ranges*:<br>12121 – 12406 (0.173455)<br>13608 – 13684 (0.031648)<br>13696 – 14756 (0.794897)<br>*Two Sigma Ranges*:<br>12014 – 12869 (0.206384)<br>13436 – 15728 (0.793616) |
| | Menengai | *no lake/no dates* | *One Sigma Ranges:*<br>4965 – 5469 (0.578404)<br>5564 – 5565 (0.000722)<br>12073 – 12564 (0.420873)<br>*Two Sigma Ranges:*<br>4840 – 5593 (0.511361)<br>11620 – 11683 (0.009513)<br>11686 – 12719 (0.479127) | *no lake/no dates* |
| Northern River | Baringo-Bogoria | *One Sigma Ranges*:<br>3851 – 4085 (0.090095)<br>4974 – 5018 (0.016382)<br>5030 – 5298 (0.102728)<br>6790 – 7843 (0.790795)<br>*Two Sigma Ranges*:<br>3575 – 4417 (0.161979)<br>4727 – 4751 (0.001705)<br>4819 – 5486 (0.149733)<br>5508 – 5581 (0.006504)<br>6322 – 6371 (0.003697)<br>6392 – 8051 (0.674339)<br>8095 – 8105 (0.0008)<br>8122 – 8130 (0.000582)<br>8142 – 8151 (0.000661) | *One Sigma Ranges*:<br>7510 – 7544 (0.009181)<br>7555 – 7966 (0.146254)<br>8460 – 8468 (0.002357)<br>8475 – 8497 (0.005584)<br>8511 – 9911 (0.603144)<br>10708 – 11407 (0.217683)<br>11450 – 11472 (0.005516)<br>11557 – 11597 (0.010282)<br>*Two Sigma Ranges*:<br>7343 – 7346 (0.000255)<br>7417 – 12089 (0.999745) | *no dates* |
| | Suguta | *One Sigma Ranges*:<br>4871 – 5045 (0.098026)<br>8163 – 8334 (0.115458)<br>8455 – 8503 (0.489169)<br>9439 – 9554 (0.134765)<br>*Two Sigma Ranges*:<br>4847 – 5289 (0.127685)<br>8022 – 8380 (0.127339)<br>8416 – 9028 (0.383551)<br>9333 – 9338 (0.000563)<br>9405 – 9631 (0.116378)<br>9647 – 9655 (0.000896) | *One Sigma Ranges*:<br>6634 – 6790 (0.134967)<br>7326 – 7399 (0.051313)<br>7410 – 7525 (0.119973)<br>7527 – 7565 (0.029519)<br>8654 – 8671 (0.010704)<br>8699 – 9034 (0.365062)<br>9050 – 9085 (0.022831)<br>9433 – 9552 (0.185197)<br>11249 – 11358 (0.070677)<br>11375 – 11390 (0.009757)<br>*Two Sigma Ranges*:<br>6492 – 6895 (0.164983)<br>7266 – 7588 (0.168263)<br>8600 – 9144 (0.331329)<br>9169 – 9250 (0.010714)<br>9311 – 9359 (0.007005)<br>9397 – 9631 (0.155636)<br>9647 – 9655 (0.000781)<br>11199 – 11719 (0.160296)<br>11737 – 11747 (0.000992) | *One Sigma Ranges*:<br>13983 – 14221 (0.13715)<br>*Two Sigma Ranges*:<br>13776 – 14687 (0.243588) |
| | Turkana | *One Sigma Ranges*:<br>3639 – 3837 (0.034644)<br>4158 – 4174 (0.002662)<br>4176 – 4203 (0.005202)<br>4224 – 5754 (0.866892)<br>5826 – 5878 (0.009566) | *One Sigma Ranges*:<br>6802 – 6814 (0.003189)<br>6847 – 6863 (0.004349)<br>6869 – 6882 (0.003766)<br>8607 – 9604 (0.785724)<br>10875 – 10942 (0.024026) | *One Sigma Ranges*:<br>12431 – 2471 (0.083021)<br>12522 – 12763 (0.916979)<br>*Two Sigma Ranges*:<br>12161 – 12207 (0.014779)<br>12231 – 12353 (0.045286) |

| | | | | |
|---|---|---|---|---|
| | | 9968 – 9982 (0.002543)<br>10155 – 10518 (0.078491)<br>*Two Sigma Ranges*:<br>3450 – 6324 (0.741544)<br>6329 – 6348 (0.000817)<br>6368 – 6394 (0.001123)<br>6793 – 7290 (0.040704)<br>7621 – 8011 (0.034587)<br>8443 – 8994 (0.034554)<br>9707 – 9718 (0.000485)<br>9733 – 10721 (0.109791)<br>11320 – 11723 (0.035663)<br>11733 – 11749 (0.000731) | 11077 – 11252 (0.178945)<br>*Two Sigma Ranges*:<br>6535 – 7179 (0.093926)<br>7213 – 7240 (0.001631)<br>8465 – 8465 (0.000055)<br>8478 – 8495 (0.001018)<br>8513 – 9916 (0.611416)<br>10098 – 10111 (0.000738)<br>10564 – 12015 (0.291216) | 12371 – 12975 (0.939935) |
| Southern River | Naivasha | *One Sigma Ranges*:<br>1558 – 1722 (0.197652)<br>3067 – 3360 (0.664182)<br>4589 – 4591 (0.001961)<br>4614 – 4709 (0.095148)<br>4754 – 4766 (0.010991)<br>4784 – 4814 (0.030065)<br>*Two Sigma Ranges*:<br>1417 – 1459 (0.010186)<br>1517 – 1868 (0.230346)<br>2996 – 3381 (0.513014)<br>4429 – 4855 (0.246455) | *One Sigma Ranges*:<br>6297 – 6633 (0.409336)<br>10236 – 10569 (0.321667)<br>10793 – 10964 (0.140759)<br>11005 – 11023 (0.014298)<br>11065 – 11197 (0.113939)<br>*Two Sigma Ranges*:<br>6207 – 6737 (0.337962)<br>9939 – 9992 (0.007035)<br>10008 – 10030 (0.002562)<br>10035 – 10062 (0.003387)<br>10127 – 11356 (0.647936)<br>11378 – 11388 (0.001118) | *One Sigma Ranges*:<br>12418 – 13085 (0.467764)<br>13946 – 14715 (0.532236)<br>*Two Sigma Ranges*:<br>11831 – 11884 (0.004877)<br>11937 – 13362 (0.483944)<br>13742 – 15091 (0.511179) |
| | Siriata | *no lake/no dates* | *One Sigma Ranges*:<br>8325 – 8407 (0.058948)<br>9426 – 10874 (0.89379)<br>10945 – 11075 (0.047262)<br>*Two Sigma Ranges*:<br>7610 – 8010 (0.044789)<br>8204 – 8264 (0.007156)<br>8285 – 8421 (0.049488)<br>8815 – 8823 (0.000544)<br>8875 – 8875 (0.000067)<br>8979 – 11997 (0.897957) | *One Sigma Ranges*:<br>12155 – 12438 (0.810331)<br>12461 – 12527 (0.189669)<br>*Two Sigma Ranges:*<br>12005 – 12628 (1.0) |
| | Magadi-Natron | *One Sigma Ranges*:<br>5589 – 5762 (0.194649)<br>5809 – 5888 (0.070305)<br>6749 – 6769 (0.015671)<br>6771 – 7480 (0.719375)<br>*Two Sigma Ranges*:<br>5340 – 5340 (0.000133)<br>5467 – 5991 (0.270677)<br>6209 – 6254 (0.006523)<br>6259 – 7578 (0.722667) | *One Sigma Ranges*:<br>8219 – 8238 (0.005786)<br>8307 – 9888 (0.994214)<br>*Two Sigma Ranges*:<br>8166 – 11709 (0.990765)<br>12174 – 12209 (0.00231)<br>12236 – 12239 (0.000271)<br>12271 – 12354 (0.005635)<br>12369 – 12383 (0.001019) | *no shoreline dates* |

[1] CPDs based on $^{14}C$ dates listed in SI Tables 2 and 3.

**Table S7.** Oxygen isotope measurements on mollusk shells from Siriata lake sediment outcrops.

| # | Outcrop ID | Depth below top (cm) | Analyzed material | δ$^{18}$O (‰) | Uncorrected shell $^{14}$C-date (yr BP) | Median age (yr cal BP)[1] |
|---|---|---|---|---|---|---|
| 1 | KOO15-1A | 0 | *Corbicula* | -2.334 | 11080 ± 60 | 7844 |
| 2 | KOO15-1A | 0 | *Corbicula* | -0.275 | 11080 ± 60 | 7844 |
| 3 | KOO15-1A | 0 | *Corbicula* | -0.370 | 11080 ± 60 | 7844 |
| 4 | KOO15-1A | 0 | *Corbicula* | -0.476 | 11080 ± 60 | 7844 |
| 5 | KOO15-1A | 10 | *Corbicula* | -0.130 | | 7944 |
| 6 | KOO15-1A | 10 | *Corbicula* | -0.962 | | 7944 |
| 7 | KOO15-1A | 10 | *Corbicula* | 0.214 | | 7944 |
| 8 | KOO15-1A | 10 | *Corbicula* | 0.318 | | 7944 |
| 9 | KOO15-1A | 30 | *Corbicula* | -0.326 | | 8151 |
| 10 | KOO15-1A | 30 | *Corbicula* | -0.786 | | 8151 |
| 11 | KOO15-1A | 40 | *Corbicula* | -1.527 | | 8252 |
| 12 | KOO15-1A | 40 | *Corbicula* | -0.034 | | 8252 |
| 13 | KOO15-1A | 40 | *Corbicula* | -0.891 | | 8252 |
| 14 | KOO15-1A | 40 | *Corbicula* | -1.030 | | 8252 |
| 15 | KOO15-1A | 50 | *Corbicula* | 0.367 | | 8355 |
| 16 | KOO15-1A | 50 | *Corbicula* | -2.174 | | 8355 |
| 17 | KOO15-1A | 50 | *Corbicula* | 1.406 | | 8355 |
| 18 | KOO15-1A | 50 | *Corbicula* | 1.943 | | 8355 |
| 19 | KOO15-1A | 60 | *Corbicula* | -0.347 | | 8454 |
| 20 | KOO15-1A | 60 | *Corbicula* | -0.667 | | 8454 |
| 21 | KOO15-1A | 60 | *Corbicula* | -2.816 | | 8454 |
| 22 | KOO15-1A | 60 | *Corbicula* | -1.335 | | 8454 |
| 23 | KOO15-1A | 70 | *Corbicula* | 1.443 | | 8557 |
| 24 | KOO15-1A | 70 | *Corbicula* | -0.506 | | 8557 |
| 25 | KOO15-1A | 100 | *Corbicula* | -1.302 | 12210 ± 60 | 8862 |
| 26 | KOO15-1A | 100 | *Corbicula* | -1.075 | 12210 ± 60 | 8862 |
| 27 | KOO15-1A | 100 | *Corbicula* | -0.592 | 12210 ± 60 | 8862 |
| 28 | KOO15-1A | 100 | *Corbicula* | -0.376 | 12210 ± 60 | 8862 |
| 29 | KOO15-1A | 110 | *Corbicula* | -0.654 | | 8966 |
| 30 | KOO15-1A | 110 | *Corbicula* | -0.644 | | 8966 |
| 31 | KOO15-1A | 110 | *Melanoides* | -0.830 | | 8966 |
| 32 | KOO15-1A | 110 | *Melanoides* | -0.240 | | 8966 |
| 33 | KOO15-1A | 180 | *Corbicula* | -0.458 | 12890 ± 60 | 9661 |
| 34 | KOO15-1A | 180 | *Corbicula* | 0.245 | 12890 ± 60 | 9661 |
| 35 | KOO15-1A | 180 | *Melanoides* | 0.062 | 12890 ± 60 | 9661 |
| 36 | KOO15-1A | 180 | *Melanoides* | 0.696 | 12890 ± 60 | 9661 |
| 37 | KOO15-1A | 190 | *Corbicula* | -0.204 | | 9762 |
| 38 | KOO15-1A | 190 | *Corbicula* | -0.414 | | 9762 |
| 39 | KOO15-1A | 190 | *Melanoides* | 0.109 | | 9762 |
| 40 | KOO15-1A | 200 | *Corbicula* | -0.273 | | 9862 |
| 41 | KOO15-1A | 200 | *Corbicula* | 0.775 | | 9862 |
| 42 | KOO15-1A | 200 | *Melanoides* | 0.289 | | 9862 |
| 43 | KOO15-1A | 200 | *Melanoides* | 0.691 | | 9862 |
| 44 | KOO15-1A | 230 | *Corbicula* | 0.682 | | 10158 |
| 45 | KOO15-1A | 230 | *Corbicula* | -1.185 | | 10158 |
| 46 | KOO15-1A | 230 | *Corbicula* | 0.770 | | 10158 |
| 47 | KOO15-1A | 230 | *Corbicula* | 0.877 | | 10158 |
| 48 | KOO15-1A | 270 | *Corbicula* | -0.824 | 13580 ± 110 | 10567 |
| 49 | KOO15-1A | 270 | *Corbicula* | -0.530 | 13580 ± 110 | 10567 |
| 50 | KOO15-1A | 270 | *Corbicula* | 0.016 | 123580 ± 110 | 10567 |

| | | | | | | |
|---|---|---|---|---|---|---|
| 51 | KOO15-1A | 270 | *Corbicula* | 0.326 | 123580 ± 110 | 10567 |
| 52 | KOO15-3E | 430 | *Corbicula* | -0.987 | 12660 ± 70 | 9379 |
| 53 | KOO15-3E | 430 | *Corbicula* | -1.052 | 12660 ± 70 | 9379 |
| 54 | KOO15-3E | 430 | *Melanoides* | -0.360 | 12660 ± 70 | 9379 |
| 55 | KOO15-3E | 430 | *Melanoides* | 0.202 | 12660 ± 70 | 9379 |
| 56 | KOO15-3E | 475 | *Corbicula* | -0.832 | | 9763 |
| 57 | KOO15-3E | 475 | *Corbicula* | -0.648 | | 9763 |
| 58 | KOO15-3E | 475 | *Corbicula* | -0.637 | | 9763 |
| 59 | KOO15-3E | 475 | *Corbicula* | -0.468 | | 9763 |
| 60 | KOO15-3E | 490 | *Corbicula* | -1.100 | 12900 ± 90 | 9890 |
| 61 | KOO15-3E | 490 | *Corbicula* | -1.107 | 12900 ± 90 | 9890 |
| 62 | KOO15-3E | 490 | *Melanoides* | -0.949 | 12900 ± 90 | 9890 |
| 63 | KOO15-3E | 490 | *Melanoides* | -1.001 | 12900 ± 90 | 9890 |
| 64 | KOO15-3E | 525 | *Corbicula* | -1.362 | | 10190 |
| 65 | KOO15-3E | 525 | *Melanoides* | -0.338 | | 10190 |
| 66 | KOO15-3E | 525 | *Melanoides* | -0.226 | | 10190 |
| 67 | KOO15-3E | 570 | *Corbicula* | -0.785 | 13040 ± 70 | 10569 |
| 68 | KOO15-3E | 570 | *Corbicula* | 0.400 | 13040 ± 70 | 10569 |
| 69 | KOO15-3E | 570 | *Melanoides* | -0.021 | 13040 ± 70 | 10569 |
| 70 | KOO15-3E | 570 | *Melanoides* | 0.001 | 13040 ± 70 | 10569 |
| 71 | KOO15-3E | 600 | *Corbicula* | 0.336 | | 10863 |
| 72 | KOO15-3E | 600 | *Corbicula* | 0.620 | | 10863 |
| 73 | KOO15-3E | 600 | *Melanoides* | -0.137 | | 10863 |
| 74 | KOO15-3E | 600 | *Melanoides* | 0.239 | | 10863 |
| 75 | KOO15-3E | 630 | *Corbicula* | -0.290 | | 11158 |
| 76 | KOO15-3E | 630 | *Corbicula* | -1.065 | | 11158 |
| 77 | KOO15-3E | 630 | *Melanoides* | -0.501 | | 11158 |
| 78 | KOO15-3E | 630 | *Melanoides* | -0.496 | | 11158 |
| 79 | KOO15-3E | 650 | *Corbicula* | -0.071 | | 11353 |
| 80 | KOO15-3E | 650 | *Corbicula* | -0.135 | | 11353 |
| 81 | KOO15-3E | 650 | *Melanoides* | 0.007 | | 11353 |
| 82 | KOO15-3E | 650 | *Melanoides* | 0.123 | | 11353 |
| 83 | KOO15-3E | 660 | *Corbicula* | -0.810 | | 11452 |
| 84 | KOO15-3E | 660 | *Corbicula* | -0.559 | | 11452 |
| 85 | KOO15-3E | 660 | *Melanoides* | 0.742 | | 11452 |
| 86 | KOO15-3E | 660 | *Melanoides* | 0.729 | | 11452 |
| 87 | KOO15-3E | 666 | *Melanoides* | -0.368 | 14060 ± 180 | 11508 |
| 88 | KOO15-3E | 666 | *Melanoides* | -0.971 | 14060 ± 180 | 11508 |
| 89 | KOO15-3E | 666 | *Melanoides* | -0.610 | 14060 ± 180 | 11508 |
| 90 | KOO15-3E | 666 | *Melanoides* | -0.734 | 14060 ± 180 | 11508 |

[1]Calibrated median ages derived from BACON age models (based on reservoir correction).

**Table S8**. Early Holocene (12-8 ka) pollen records from East African sites.

| # | Site | Latitute (°) | Longitude (°) | Eleva-tion (m) | Time interval with pollen data (mean cal. BP) | # of pollen samples included | Percent Tree Pollen (aver-age) | Notes | Data source | Original references |
|---|---|---|---|---|---|---|---|---|---|---|
| 1 | Ahakagyezi Swamp | -1.116667 | 29.900000 | 1830 | 8356 - 11626 | 6 | 65 | core AH2 | APD/Neotoma | 109 |
| 2 | Kashiru Swamp | -3.450000 | 29.533333 | 2240 | 8004 - 11981 | 35 | 44 | Combined pollen data of core KA1 (11 samples) and core KA3 (24 samples) | APD/Neotoma | 110 |
| 3 | Kiguhu Swamp | -1.483330 | 29.716670 | 1800 | 8317 - 11964 | 17 | 59 | | APD/Neotoma | 111, 112, 113 |
| 4 | Lake Albert | 1.520000 | 30.575000 | 619 | 8154 - 11695 | 6 | 53 | WHOI core 3PC | APD/Neotoma | 114, 115, 116 |
| 5 | Lake Albert | 1.833330 | 31.166670 | 619 | 9461 - 10568 | 3 | 75 | | APD/Neotoma | 117, 118 |
| 6 | Lake Kimili (Mt. Elgon) | 1.100000 | 34.566940 | 4150 | 8072 - 11936 | 22 | 26 | Combined pollen data (of core A (6 samples) and core B (16 samples) | APD/Neotoma | 119, 120 |
| 7 | Lake Tanganyika | -4.080965 | 29.366290 | 773 | 8460 - 11822 | 11 | 50 | Combined pollen data of core SD14TAN (8 samples) and core SD24TAN (3 samples) | APD/Neotoma | 121, 122 |
| 8 | Mubwindi Swamp | -1.081940 | 29.758330 | 2100 | 9365 - 11123 | 4 | 64 | Combined pollen data of core MB3 (2 samples) and core MB6 (2 samples) | APD/Neotoma | 123, 124 |
| 9 | Muchoya Swamp | -1.283330 | 29.800000 | 2260 | 8103 - 8459 | 2 | 56 | core MC2 | APD/Neotoma | 109, 125 |
| 10 | Mukibongo Valley | -3.158330 | 30.583330 | 1540 | 8650 | 1 | 36 | | APD/Neotoma | 126 |
| 11 | Mukukwe Swamp | -2.500000 | 28.550000 | 1470 | 8440 - 11835 | 10 | 41 | | APD/Neotoma | 127 |
| 12 | Musisi-Karashoma | -2.271110 | 28.660280 | 2200 | 8482 - 10412 | 4 | 59 | | APD/Neotoma | 128, 129 |
| 13 | Nyamuswaga Valley | -2.900000 | 29.983330 | 1546 | 8202 - 11732 | 4 | 82 | | APD/Neotoma | 126 |
| 14 | Rugezi Swamp | -1.400000 | 29.833330 | 2050 | 8589 - 11724 | 3 | 45 | | APD/Neotoma | 111, 113, 130 |
| 15 | Rumuiku Swamp | -0.462340 | 37.535985 | 2154 | 8166 - 11939 | 9 | 66 | | APD/Neotoma | 131 |
| 16 | Rusaka Swamp | -3.433330 | 29.616670 | 2070 | 8071 - 11822 | 40 | 59 | | APD/Neotoma | 132 |
| 17 | Lake Kitandara | 0.347950 | 29.886370 | 3990 | 8284 - 8631 | 6 | 44 | | APD/Neotoma | 133 |
| 18 | Lake Mahoma | 0.344245 | 29.967205 | 2960 | 8330 - 11654 | 9 | 31 | | APD/Neotoma | 119, 133, 134 |
| 19 | Sacred Lake (Mt. Kenya) | 0.047930 | 37.528870 | 2345 | 8194 - 11889 | 7 | 62 | | APD/Neotoma | 135, 136 |
| 20 | Lake Magadi | -1.851600 | 36.279400 | 607 | 9058 - 11989 | 13 | 11 | Core MAG14-2A | Raw data reported in SI | 137 |

| | | | | | | | | | | |
|---|---|---|---|---|---|---|---|---|---|---|
| | | | | | | | | | of original paper. Used published age-model. | |
| 21 | Lake Victoria | 0.300000 | 33.333333 | 1135 | c. 8080 - 11500 | 8 | 77 | $^{14}C$ ages calibrated. | Percentage data from Fig. 13 of original paper. | 138 |
| 22 | Lake Naivasha | -0.750000 | 36.333333 | 1890 | c. 8000 - 11500 | 6 | 21 | $^{14}C$ ages calibrated. Core described in ref. 32. | Percentage data from Fig. 3 of original paper. | 139 |
| 23 | Ngorongoro (Empakaai Crater Lake) | -2.916667 | 35.833333 | 2300 | c. 9300 - 11700 | 17 | 43 | | Pollen data from Fig. 5 of original paper. | 140 |
| 24 | Mau Forest (Nyabuiyabui wetland) | -0.436467 | 35.799650 | 2920 | c. 8000 - 11800 | 14 | 36 | | Pollen data from Fig. 5 of original paper (‘Afromontane trees and shrubs’). | 141 |
| 25 | Lake Bogoria | 0.327142 | 36.083184 | 990 | c. 8000 - 12000 | 42 | 13 | Core BOG III. Reservoir correction of 3980 $^{14}C$ years (ref. 5). | Pollen data from Fig. 4 of original paper (tree and shrubs). | 142 |

Tree pollen data plotted in Fig. 3*C*.

## SI References


1. J. A. T. Young, R. W. Renaut, A radiocarbon date from Lake Bogoria, Kenya Rift valley. *Nature* **278**, 243–245 (1979).
2. J. J. Tiercelin, R. W. Renaut, G. Delibrias, J. Le Fournier, S. Bieda, Late Pleistocene and Holocene lake level fluctuations in the Lake Bogoria basin, northern Kenya Rift Valley. *Palaeoecol. Afr.* **13**, 105–120 (1981).
3. H. Tichy, L. Seegers, The *Oreochromis alcalicus* flock (Teleostei Cichlidae) from lakes Natron and Magadi, Tanzania and Kenya: a model for the evolution of new species flocks in historical times. *Ichthyol. Explor. Freshw.* **10**, 147–174 (1999).
4. Y. Garcin *et al.*, Late Pleistocene–Holocene rise and collapse of Lake Suguta, northern Kenya Rift. *Quat. Sci. Rev.* **28**, 911–925 (2009).
5. G. De Cort *et al.*, Late-Holocene and recent hydroclimatic variability in the central Kenya Rift Valley: the sediment record of hypersaline lakes Bogoria, Nakuru and Elementeita. *Palaeogeogr. Palaeoclimatol. Palaeoecol.* **388**, 69–80 (2013).
6. Y. Garcin *et al.*, Short-lived increase in erosion during the African Humid Period: Evidence from the northern Kenya Rift. *Earth Planet. Sci. Lett.* **459**, 58–69 (2017).
7. H. Lee *et al.*, Massive and prolonged deep carbon emissions associated with continental rifting. *Nat. Geosci.* **9**, 145-149 (2016).
8. Y. Garcin, D. Melnick, M. R. Strecker, D. Olago, J. J. Tiercelin, East African mid-Holocene wet–dry transition recorded in palaeo-shorelines of Lake Turkana, northern Kenya Rift. *Earth Planet. Sci. Lett.* **331**, 322–334 (2012).
9. A. Junginger, S. Roller, L. A. Olaka, M. H. Trauth, The effects of solar irradiation changes on the migration of the Congo Air Boundary and water levels of paleo-Lake Suguta, Northern Kenya Rift, during the African Humid Period (15–5 ka BP). *Palaeogeogr. Palaeoclimatol. Palaeoecol.* **396**, 1–16 (2014).
10. P. N. Dunkley, M. Smith, D. J. Allen, W. G. Darling, *The geothermal activity and geology of the northern sector of the Kenya Rift Valley*. (British Geological Survey, Nottingham, 1993), pp. 202.
11. C. Tarits *et al.*, Geochemical evidence of hydrothermal recharge in Lake Baringo, central Kenya Rift Valley. *Hydrol. Process.* **20**, 2027–2055 (2006).
12. R. W. Renaut, R. B. Owen, J. K. Ego, Geothermal activity and hydrothermal mineral deposits at southern Lake Bogoria, Kenya Rift Valley: Impact of lake level changes. *J. Afr. Earth Sci*. **129**, 623–646 (2017).
13. W. G. Darling, E. Griesshaber, J. N. Andrews, H. Armannsson, R. K. O'nions, The origin of hydrothermal and other gases in the Kenya Rift Valley. *Geochim. Cosmochim. Acta* **59**, 2501–2512 (1995).
14. J. J. Tiercelin, A. Vincens, Le demi-graben de Baringo-Bogoria, Rift Gregory, Kenya. 30000 ans d'histoire hydrologique et sédimentaire. *Bull. Cent. Rech. Explor. Prod. Elf-Aquitaine* **11**, 249–540 (1987).
15. M. Taieb, P. Barker, A. Vincens, D. Williamson, R. Bonnefille, Histoire paléohydrologique du lac Magadi (Kenya) au Pleistocène superieur. *C. R. Acad. Sci. II* **313**, 339–346 (1991).
16. N. Roberts *et al.*, Timing of the Younger Dryas event in East Africa from lake-level changes. *Nature* **366**, 146–148 (1993).
17. P. J. Reimer *et al.*, IntCal13 and Marine13 Radiocarbon Age Calibration Curves 0–50,000 years cal BP. *Radiocarbon* **55**, 1869–1887 (2013).
18. D. Melnick *et al.*, Steady rifting in northern Kenya inferred from deformed Holocene lake shorelines of the Suguta and Turkana basins. *Earth Planet. Sci. Lett.* **331**, 335–346 (2012).
19. S. L. Forman, D. K. Wright, C. Bloszies, Variations in water level for Lake Turkana in the past 8500 years near Mt. Porr, Kenya and the transition from the African Humid Period to Holocene aridity. *Quat. Sci. Rev.* **97**, 84–101 (2014).
20. A. Morrissey, C. A. Scholz, Paleohydrology of Lake Turkana and its influence on the Nile River system. *Palaeogeogr. Palaeoclimatol. Palaeoecol.* **403**, 88–100 (2014).

21. C. Bloszies, S. L. Forman, D. K. Wright, Water level history for Lake Turkana, Kenya in the past 15,000 years and a variable transition from the African Humid Period to Holocene aridity. *Glob. Planet. Change* **132**, 64–76 (2015).
22. C. C. Beck, C. S. Feibel, J. D. Wright, R. A. Mortlock, Onset of the African humid period by 13.9 kyr BP at Kabua Gorge, Turkana Basin, Kenya. *Holocene* 29, 1011–1019 (2019).
23. C. C. Beck *et al.*, Living in a swampy paradise: Paleoenvironmental reconstruction of an African Humid Period lacustrine margin, West Turkana, Kenya. *J. Afr. Earth Sci*. **154**, 20–34 (2019).
24. W. W. Bishop, "Geological reconnaissance of the lower Suguta Valley and its eastern flank, north Kenya rift Valley" in *Cambridge Meeting on Desertification*, A. T. Grove, Ed. (1975) pp. 62–63.
25. P. H. Truckle, Geology and late Cainozoic lake sediments of the Suguta Trough, Kenya. *Nature* **263**, 380-383 (1976).
26. J. Casanova, C. Hillaire-Marcel, N. Page, M. Taieb, A. Vincens, Stratigraphie et paléohydrologie des épisodes lacustres du Quaternaire récent du rift Suguta (Kenya). *C. R. Acad. Sci. II* **307**, 1251–1258 (1988).
27. R. W. Renaut, *Late Quaternary Geology of the Lake Bogoria Fault-trough, Kenya Rift Valley*. Ph.D. thesis. (University of London, London, 1982).
28. A. Vincens, J. Casanova, J. J. Tiercelin, "Palaeolimnology of Lake Bogoria (Kenya) during the 4500 BP high lacustrine phase" in *Sedimentation in the African Rifts, 25. Geological Society of London Special Publication*, L. E. Frostick, R. W. Renaut, I. Reid, J. J. Tiercelin, Eds. (Blackwell Scientific Publications, Oxford, 1986), pp. 323–330.
29. P. T. Leat, Geological evolution of the trachytic caldera volcano Menengai, Kenya Rift Valley. *J. Geol. Soc. London* **141**, 1057–1069 (1984).
30. S. Riedl, D. Melnick, G. K. Mibei, L. Njue, M. R. Strecker, Continental rifting at magmatic centres: structural implications from the Late Quaternary Menengai Caldera, central Kenya Rift. *J. Geol. Soc. London* **177**, 153–169 (2020).
31. J. L. Richardson, Palaeolimnological records from Rift Lakes in Central Kenya. *Palaeoecol. Afr.* **6**, 131–136 (1972).
32. J. L. Richardson, R. A. Dussinger, Paleolimnology of mid-elevation lakes in the Kenya Rift Valley. *Hydrobiologia* **143**, 167–174 (1986).
33. R. Protsch, The Naivasha hominid and its confirmed late Upper Pleistocene age. *Anthropol. Anz.* **35**, 97–102 (1976).
34. J. L. Richardson, A. E. Richardson, History of an African rift lake and its climatic implications. *Ecol. Monogr.* **42**, 499–534 (1972).
35. C. Hillaire-Marcel, O. Carro, J. Casanova, $^{14}$C and Th/U Dating of Pleistocene and Holocene Stromatolites from East African Paleolakes. *Quat. Res.* **25**, 312–329 (1986).
36. G. L. Isaac, H. V. Merrick, C. M. Nelson, Stratigraphic and archaeological studies in the Lake Nakuru Basin. *Palaeoecol. Afr.* **6**, 225–232 (1972).
37. A. L. Deino *et al.*, Chronostratigraphic model of a high-resolution drill core record of the past million years from the Koora Basin, south Kenya Rift: Overcoming the difficulties of variable sedimentation rate and hiatuses. *Quat. Sci. Rev.* **215**, 213–231 (2019).
38. A. L. Deino *et al.*, Chronostratigraphy of the Baringo-Tugen Hills-Barsemoi (HSPDP-BTB13-1A) core – $^{40}$Ar/$^{39}$Ar dating, magnetostratigraphy, tephrostratigraphy, sequence stratigraphy and Bayesian age modeling. *Palaeogeogr. Palaeoclimatol. Palaeoecol.* **570**, 109519 (2021).
39. E. M. Niespolo, D. Rutte, A. L. Deino, P. R. Renne, Intercalibration and age of the Alder Creek sanidine Ar-40/Ar-39 standard. *Quat. Geochronol*. **39**, 205–213 (2017).
40. I. McDougall, T.M. Harrison, *Geochronology and Thermochronology by the* $^{40}$*Ar/*$^{39}$*Ar Method* (Oxford University Press, Oxford, New York, 1999).
41. J.-Y. Lee *et al.*, A redetermination of the isotopic abundances of atmospheric Ar. *Geochem. Cosmochim. Acta* **70**, 4507–4512 (2006).
42. K. W. Min, R. Mundil, P. R. Renne, K. R. Ludwig, A test for systematic errors in Ar-40/Ar-39 geochronology through comparison with U/Pb analysis of a 1.1-Ga rhyolite. *Geochim. Cosmochim. Acta* **64** 73–98 (2000).
43. R. J. Fleck, J. F. Sutter, D. H. Elliot, Interpretation of discordant $^{40}$Ar/$^{39}$Ar age-spectra of Mesozoic tholeiites from Antarctica. *Geochem. Cosmochim. Acta* **41**, 15–32 (1977).

44. C. B. Keller, B. Schoen, K. M. Samperton, A stochastic sampling approach to zircon eruption age interpretation. *Geochem. Perspect. Lett.* **8** 31–35 (2018).
45. S. E. Nicholson, Climate and climatic variability of rainfall over eastern Africa. *Rev. Geophys.* **55**, 590–635 (2017).
46. A. G. Bergner *et al.*, Tectonic and climatic control on evolution of rift lakes in the Central Kenya Rift, East Africa. *Quat. Sci. Rev.* **28**, 2804–2816 (2009).
47. L. A. Olaka, E. O. Odada, M. H. Trauth, D. O. Olago, The sensitivity of East African rift lakes to climate fluctuations. *J. Paleolimnol*. **44**, 629–644 (2010).
48. J. E. Kutzbach, Monsoon climate of the early Holocene: climate experiment with the earth's orbital parameters for 9000 years ago. *Science* **214**, 59–61 (1981).
49. J. E. Kutzbach, F. A. Street-Perrott, Milankovitch forcing of fluctuations in the level of tropical lakes from 18 to 0 kyr BP. *Nature* **317**, 130–134 (1985).
50. B. L. Otto-Bliesner *et al.*, Coherent changes of southeastern equatorial and northern African rainfall during the last deglaciation. *Science* **346**, 1223–1227 (2014).
51. F. A. Street-Perrott, R. A. Perrott, Abrupt climate fluctuations in the tropics: the influence of Atlantic Ocean circulation. *Nature* **343**, 607–612 (1990).
52. D. Verschuren *et al.*, Half-precessional dynamics of monsoon rainfall near the East African Equator. *Nature* **462**, 637–641 (2009).
53. J. E. Tierney, S. C. Lewis, B. I. Cook, A. N. LeGrande, G. A. Schmidt, Model, proxy and isotopic perspectives on the East African Humid Period. *Earth Planet. Sci. Lett.* **307**, 103–112 (2011).
54. A. G. Bergner, M. H. Trauth, B. Bookhagen, Paleoprecipitation estimates for the Lake Naivasha basin (Kenya) during the last 175 ky using a lake-balance model. *Glob. Planet. Change* **36**, 117–136 (2003).
55. H. Tarras-Wahlberg, M. Everard, D. M. Harper, Geochemical and physical characteristics of river and lake sediments at Naivasha, Kenya. *Hydrobiologia* **488**, 27–41 (2002).
56. L. A. Olaka *et al.*, Groundwater fluoride enrichment in an active rift setting: Central Kenya Rift case study. *Sci. Total Environ.* **545**, 641–653 (2016).
57. TRMM, 2011. The ungridded TRMM 2B31 data (Version 7). Available at https://disc.gsfc.nasa.gov/datasets/TRMM_2B31_7/summary.
58. IUCN, 2020. The International Union for Conservation of Nature's Red List of Threatened Species. Version 2020-3. Available at https://www.iucnredlist.org. Accessed 11 March 2020.
59. J. Kingdon, *East African mammals. Volume I* (Academic Press, London, 1971), pp. 446.
60. J. Kingdon, *East African mammals. Volume III, Part A (Carnivores)* (Academic Press, London, 1977), pp. 476.
61. J. Kingdon, *East African mammals. Volume III, Part B (Large Mammals)* (Academic Press, London, 1979), pp. 436.
62. J. Kingdon, *East African mammals. Volume III, Part C (Bovids)* (Academic Press, London, 1982), pp. 393.
63. R. E. G. Williams, A. S. Johnson, Birmingham University Radiocarbon Dates X. *Radiocarbon* **18**, 249–267 (1976).
64. W. R. Farrand, R. R. Redding, M. H. Wolpoff, H. T. Wright III, *An archaeological investigation on the Loboi Plain, Baringo District, Kenya. Research Reports in Archeology Contribution 1* (Museum of Anthropology, University of Michigan, Ann Arbor, 1976), pp. 79.
65. A. T. Hopwood, "Appendix C. Preliminary report on the fossil Mammalia" in *The Stone Age Cultures of Kenya Colony*, L. S. B. Leakey, Ed. (Cambridge University Press, London, 1931) pp. 271–275.
66. D. P. Gifford-Gonzalez, Faunal assemblages from Masai Gorge rockshelter and Marula rockshelter. *Azania* **20**, 69–88 (1985).
67. C. W. Marean, Hunter to herder: large mammal remains from the hunter-gatherer occupation at Enkapune ya Muto rock-shelter, Central Rift, Kenya. *Afr. Archaeol. Rev.* **10**, 65–127 (1992).
68. L. Seegers, L. De Vos, D. O. Okeyo, Annotated checklist of the freshwater fishes of Kenya (excluding the lacustrine haplochromines from Lake Victoria). *J. East Afr. Nat. Hist.* **92**, 11–47 (2003).
69. S. B. Wandera, J. S., Balirwa, Fish species diversity and relative abundance in Lake Albert—Uganda. *Aquat. Ecosyst. Health Manag*. **13**, 284–293 (2010).

70. C. Lévêque, D. Paugy, G. G. Teugels, Annotated check-list of the freshwater fishes of the Nilo-sudan river basins, in Africa. *Rev. Hydrobiol. Trop.* **24**, 131–154 (1991).
71. E. Decru, N. Vranken, P. H. Bragança, J. Snoeks, M. Van Steenberge, Where ichthyofaunal provinces meet: the fish fauna of the Lake Edward system, East Africa. *J. Fish Biol.* **96**, 1186–1201 (2020).
72. www.fishbase.se (accessed May 5 2021)
73. www.Gbif.org (accessed May 5 2021)
74. L. Seegers, H. Tichy, The *Oreochromis alcalicus* flock (Teleostei: Cichlidae) from Lakes Natron and Magadi, Tanzania and Kenya with descriptions of two new species. *Ichthyol. Explor. Freshw.* **10**, 97–146 (1999).
75. P. H. Greenwood, A revision of the *Haplochromis* and related species (Pisces: Cichlidae) from Lake George, Uganda. *Bull. Br. Mus. Nat. Hist. (Zool.)* **25**, 141–242 (1973).
76. E. Lippitsch, Redescription of *Haplochromis nubilus* (Teleostei: Cichlidae), with description of two new species. *Ichthyol. Explor. Freshw.* **14**, 85–95 (2003).
77. N, Vranken, M. Van Steenberge, J. Snoeks, Similar ecology, different morphology: Three new species of oral-mollusc shellers from Lake Edward. *J. Fish Biol.* **96**, 1202–1217 (2020).
78. P. H. Greenwood, Towards a phyletic classification of the 'genus' *Haplochromis* (Pisces, Cichlidae) and related taxa. Part II; the species from Lakes Victoria, Nabugabo, Edward, George and Kivu. *Bull. Br. Mus. Nat. Hist. (Zool.)* **39**, 1–101 (1980).
79. N. Vranken, M. Van Steenberge, A. Balagizi, J. Snoeks, The synonymy of *Haplochromis pharyngalis* and *Haplochromis petronius* (Cichlidae). *J. Fish Biol.* **97**, 1554–1559 (2020).
80. N. Vranken, M. Van Steenberge, J. Snoeks, Grasping ecological opportunities: not one but five paedophagous species of *Haplochromis* (Teleostei: Cichlidae) in the Lake Edward system. *Hydrobiologia* **832**, 105–134 (2019).
81. N. Vranken, M. Van Steenberge, A. Kayenbergh, J. Snoeks, The lobed-lipped species of *Haplochromis* (Teleostei, Cichlidae) from Lake Edward, two instead of one. *J. Great Lakes Res.* **46**, 1079–1089 (2020).
82. T. C. Ndiwa, D. W. Nyingi, J. F. Agnese, An important natural genetic resource of *Oreochromis niloticus* (Linnaeus, 1758) threatened by aquaculture activities in Loboi drainage, Kenya. *PLoS ONE* **9**, e106972 (2014).
83. A. G. Ford *et al.*, Molecular phylogeny of *Oreochromis* (Cichlidae: Oreochromini) reveals mito-nuclear discordance and multiple colonisation of adverse aquatic environments. *Mol. Phylogenet. Evol.* **136**, 215–226 (2019).
84. G. G. Teugels, A systematic revision of the African species of the genus *Clarias* (Pisces; Clariidae). *Ann. Mus. R. Afr. Centr., Sci. Zool.* **247**, 1–199 (1986).
85. M. Wakjira, A. Getahun, Ichthyofaunal diversity of the Omo-Turkana basin, East Africa, with specific reference to fish diversity within the limits of Ethiopian waters. *Check List* **13**, 2059 (2017).
86. M. Hardman, A new species of *Chrysichthys* (Siluriformes: Claroteidae) from Lake Turkana, Kenya. *Proc. Acad. Nat. Sci. Philadelphia* **157**, 25–36 (2008).
87. P. H. Greenwood, A revision of certain *Barbus* species (Pisces, Cyprinidae) from east, central and South Africa. *Bull. Br. Mus. Nat. Hist. (Zool.)* **8**, 151–208 (1962).
88. H. Maetens, M. Van Steenberge, J. Snoeks, E. Decru, Revalidation of *Enteromius alberti* and presence of *Enteromius* cf. *mimus* (Cypriniformes: Cyprinidae) in the Lake Edward system, East Africa. *Eur. J. Taxon.* **700**, 1–28 (2020).
89. J. Daget, J. P. Gosse, D. F. E. Thys van den Audenaerde, *Check-list of the freshwater fishes of Africa (CLOFFA). Vol. I.* (ORSTOM, Paris, AMRAC, Tervuren, 1984), pp. 410.
90. B., Nagy, B. R. Watters, P. D. van der Merwe, F. P. Cotterill, D. U. Bellstedt, Review of the *Nothobranchius ugandensis* species group from the inland plateau of eastern Africa with descriptions of six new species (Teleostei: Nothobranchiidae). *Ichthyol. Explor. Freshw.* **30**, 21–73 (2020).
91. B. Nagy, B. R. Watters, Distribution and habitat conditions of *Nothobranchius* fishes in Uganda. *J. Am. Killifish Assoc.* **51**, 178–194 (2018).
92. R. B. Owen, J. W. Barthelme, R. W. Renaut, A. Vincens, Palaeolimnology and Archaeology of Holocene deposits north-east of Lake Turkana, Kenya. *Nature* **298**, 523–529 (1982).

93. K. W. Butzer, D. L. Thurber, Some Late Cenozoic Sedimentary Formations of the Lower Omo Basin. *Nature* **222**, 1138–1143 (1969).
94. K. W. Butzer, G. L. Isaac, J. L. Richardson, C. Washbourn-Kamau, Radiocarbon dating of East African lake levels. *Science* **175**, 1069–1076 (1972).
95. L. H. Robbins, Archeology in the Turkana District, Kenya. *Science* **176**, 359–366 (1972).
96. K. S. Thomson, Quaternary Fish Fossils from West of Lake Rudolf, Kenya. *Breviora* **243**, 1–10 (1966).
97. M. Stuiver, Yale Natural Radiocarbon Measurements IX. *Radiocarbon* **11**, 545–658 (1969).
98. F. Yamasaki, C. Hamada, T. Hamada, Riken Natural Radiocarbon Measurements VIII. *Radiocarbon* **16**, 331–357 (1974).
99. J. R. Bower, C. M. Nelson, A. F. Waibel, S. Wandibba, The University of Massachusetts' later stone age/pastoral 'Neolithic'comparative study in Central Kenya: an overview. *Azania* **12**, 119–146 (1977).
100. J. R. Bower, C. M. Nelson, Early pottery and pastoral cultures of the Central Rift Valley, Kenya. *Man* **13**, 554–566 (1978).
101. R. Protsch, The chronological position of Gamble's Cave II and Bromhead's site (Elmenteita) of the Rift Valley, Kenya. *J. Hum. Evol.* **7**, 101–109 (1978).
102. R. Berger, R. Protsch, UCLA radiocarbon dates XI. *Radiocarbon* **31**, 55–67 (1989).
103. M. Dühnforth, A. G. Bergner, M. H. Trauth, Early Holocene water budget of the Nakuru-Elmenteita basin, central Kenya Rift. *J. Paleolimnol.* **36**, 281–294 (2006).
104. C. K. Washbourn-Kamau, Late Quaternary chronology of the Nakuru–Elmenteita Basin, Kenya. *Nature* **226**, 253–254 (1970).
105. F. Yamasaki, C. Hamada, T. Hamada, Riken Natural Radiocarbon Measurements VII. *Radiocarbon* **14**, 223–238 (1972).
106. D. Gifford-Gonzalez, Early pastoralists in East Africa: Ecological and social dimensions. *J. Anthropol. Archaeol.* **17**, 166–200 (1998).
107. S. H. Ambrose, Excavations at Masai Gorge Rockshelter, Naivasha. *Azania* **20**, 29–67 (1985).
108. J. L. Richardson, Changes in level of Lake Naivasha, Kenya, during postglacial times. *Nature* **209**, 290–291 (1966).
109. D. M. Taylor, Late Quaternary pollen records from two Ugandan mires: evidence for environmental change in the Rukiga Highlands of southwest Uganda. *Palaeogeogr. Palaeoclimatol. Palaeoecol.* **80**, 283–300 (1990).
110. R. Bonnefille, F. Chalié, Pollen-inferred precipitation time-series from equatorial mountains, Africa, the last 40 kyr BP. *Glob. Planet. Change* **26,** 25–50 (2000).
111. E. Roche, "Evolution du paléoenvironnement holocène au Rwanda. Implications climatiques déduites de l'analyse palynologique de séquences sédimentaires" in: *Proceedings of the international conference: 'Tropical climatology, meteorology and hydrology', Brussels 22–24/05/1996*, G. Demarée *et al*., Eds. (IRM & ARSOM Editors, Brussels, 1998) pp. 108–127.
112. E. Roche, C. Ntaganda, Analyse palynologique de la séquence sédimentaire Kiguhu II (région des Birunga, Rwanda). Evolution du paléoenvironnement et du paléoclimat dans le domaine afro-montagnard du Rwanda au cours de l'Holocène. *Geo-Eco-Trop* **22**, 71–82 (1999).
113. C. Ntaganda, *Paléoenvironnements et paléoclimats du Quaternaire supérieur au Rwanda par l'analyse palynologique des dépôts superficiels* (Doctoral thesis, University of Liège, Belgium, 1991), pp. 281.
114. I. Nakimera-Ssemmanda, *Histoire des végétations et du climat dans le rift occidental ougandais depuis 13 000 ans B.P. Etude palynologique de séquences sédimentaires des lacs Albert et Edouard* (Mémoire de l'Ecole Pratique des Hautes Etudes, Bordeaux, 1991) pp. 125.
115. K. R. Beuning, M. R. Talbot, K. Kelts, A revised 30,000-year paleoclimatic and paleohydrologic history of Lake Albert, East Africa. *Palaeogeogr. Palaeoclimatol. Palaeoecol.* **136**, 259–279 (1997).
116. I. Ssemmanda, A. Vincens, Végétation et climat dans la Bassin du lac Albert (Ouganda, Zaïre) depuis 13 000 ans B.P.: apport de la palynologie. *C. R. Acad. Sci. II* **316**, 561–567 (1993).
117. T. J. Harvey, *The palaeolimnology of Lake Mobutu Sese Seko, Uganda-Zaire: the last 28,000 years* (Doctoral dissertation, Duke University, Durham, North Carolina, 1976) pp. 113.
118. M. A. Sowunmi, Late Quaternary environments in equatorial Africa: palynological evidence. *Palaeoecol. Afr.* **22**, 213–238 (1991).

119. A. C. Hamilton, “Upper Quaternary pollen diagrams from montane eastern Africa” in: *Environmental history of East Africa: a study of the Quaternary*, A. C. Hamilton (Academic Press, London, 1982) pp. 111–191.
120. A. C. Hamilton, Vegetation and climate of Mt Elgon during the late Pleistocene and Holocene. *Palaeoecol. Afr.* **18**, 283–304 (1987).
121. A. Vincens, Paléoenvironnements du bassin North-Tanganyika (Zaire, Burundi, Tanzanie) au cours des 13 derniers mille ans: apport de la palynologie. *Rev. Palaeobot. Palynol.* **61**, 69–88 (1989).
122. A. Vincens, Nouvelle séquence pollinique du lac Tanganyika: 30,000 ans d'histoire botanique et climatique du Bassin Nord. *Rev. Palaeobot. Palynol.* **78,** 381–394 (1993).
123. R. Marchant, D. Taylor, Dynamics of montane forest in central Africa during the late Holocene: a pollen-based record from western Uganda. *Holocene* **8**, 375–38 (1998).
124. R. Marchant, D. M. Taylor, A. Hamilton, Late Pleistocene and Holocene history at Mubwindi Swamp, Southwest Uganda. *Quat. Res.* **47**, 316–328 (1997).
125. D. M. Taylor, Pollen evidence from Muchoya Swamp, Rukiga Highlands (Uganda), for abrupt changes in vegetation during the last ca. 21,000 years. *Bull. Soc. Géol. Fr.* **163**, 77–82 (1992).
126. D. Jolly, *Evolution et dynamique des écosystèmes du Burundi. Pollen et Statistique* (MS. thesis, Université Aix-Marseille II, 1993) pp. 142.
127. S. Vilimumbalo, “Palaeoenvironmental and palaeoclimatic evolution of the Southern part of the Kivu Basin, Western branch of the African rift, during the Holocene period” in: *2nd Symposium on African Palynology, Tervuren, Belgium*. (Publication Occasionnelle du CIFEG, Orleans, 1995) pp. 145–157.
128. M. C. Moscol-Olivera, E. Roche, Analyse palynologique d'une séquence sédimentaire Holocène a Musisi-Karashoma (Kivu, R.D. Congo). Influences climatiques et anthropiques sur l'environnement. *Geo-Eco-Trop* **1-4**, 1–26 (1997).
129. M. C. Moscol-Olivera, *Analyse palynologique d'une séquence sédimentaire Holocène à Musisi-Karashoma (Kivu, R. D. Congo) - Influences climatiques et anthropiques sur l'environnement.* (Mémoire de DEA, Université de Liege, 1998) pp. 51.
130. E. Roche, M. C. Van Grunderbeek, “Apports de la palynologie à l’étude du Quaternaire supérieur au Rwanda” in: *Palynologie et milieux tropicaux, Montpellier, IXe symposium de l’A.P.L.F., Montpellier 1987* (Ecole Pratique des Hautes Etudes, Mémoires et Travaux de l’Institut de Montpellier 17, Montpellier, 1987) pp. 111–127.
131. S. M. Rucina, V.M. Muiruri, R. N. Kinyanjui, K. McGuiness, R. Marchant, Late Quaternary Vegetation and Fire Dynamics on Mt. Kenya. *Palaeogeogr. Palaeoclimatol. Palaeoecol.* **283,** 1–14 (2009).
132. R. Bonnefille *et al.*, Glacial/Interglacial record from intertropical Africa, high resolution pollen and carbon data at Rusaka, Burundi. *Quat. Sci. Rev.* **14**, 917–936 (1995).
133. D.A. Livingstone, Postglacial vegetation of the Ruwenzori Mountains in Equatorial Africa. *Ecol. Monogr.* **37**, 25–52 (1967).
134. D.A. Livingstone, Age of deglaciation in the Ruwenzori Range, Uganda. *Nature* **194,** 859–860 (1962).
135. J. Coetzee, Evidence for a considerable depression of the vegetation belts during the Upper Pleistocene on the East African Mountains. *Nature* **204**, 564–566 (1964).
136. J. Coetzee, Pollen analytical studies in East and Southern Africa. *Palaeoecol. Afr.* **3**, 1–146 (1967).
137. R. B. Owen *et al*., Progressive aridification in East Africa over the last half million years and implications for human evolution. *Proc. Natl. Acad. Sci. U.S.A.* **115**, 11174–11179 (2018).
138. R. L. Kendall, An ecological history of the Lake Victoria basin. *Ecol. Monogr.* **39**, 121–176 (1969).
139. J. M. Maitima, Vegetation response to climatic change in Central Rift Valley, Kenya. *Quat. Res.* **35**, 234–245 (1991).
140. M. A. Ryner, R. Bonnefille, K. Holmgren, A. Muzuka, Vegetation changes in Empakaai Crater, northern Tanzania, at 14,800-9300 cal yr BP. *Rev. Palaeobot. Palynol.* **140**, 163–174 (2006).
141. E. N. Githumbi, C. J. Courtney Mustaphi, R. Marchant, Late Pleistocene and Holocene Afromontane vegetation and headwater wetland dynamics within the Eastern Mau Forest, Kenya. *J. Quat. Sci.* **36**, 239–254 (2021).

142. A. Vincens, Diagramme pollinique d'un sondage Pleistocene superieur-Holocene du Lac Bogoria (Kenya). *Rev. Palaeobot. Palynol.* **47**, 169–192 (1986).
